# Supplementary material for: ‘When will this end? Will it end?’ The impact of the March–June 2020 UK COVID-19 lockdown response on mental health: a longitudinal survey of mothers in the Born in Bradford study
Source: BMJ Open. 2022 Jan 11;12(1):e047748. doi: 10.1136/bmjopen-2020-047748 (PMC8753090; doi:10.1136/bmjopen-2020-047748)
Supplement: Supplementary data [file bmjopen-2020-047748supp001.pdf]

Supplementary Table 1: The sample characteristics of those invited to complete the Covid-19 survey by survey completion status.

	Returned Survey (n=2144)			
**Age**	Complete survey (n=1860)	Incomplete / not linked (n=284)	Not returned survey (n=4722)	Total eligible (n=6866)
Under 30 yrs.	224 (12%)	4 (2%)	751 (16%)	963 (14%)
30 to 34 yrs.	396 (21%)	22 (10%)	1182 (25%)	1600 (24%)
35 to 39 yrs.	516 (28%)	66 (29%)	1367 (29%)	1939 (29%)
40 to 44 yrs.	423 (23%)	73 (32%)	892 (19%)	1395 (21%)
45 yrs. plus	301 (16%)	61 (27%)	495 (11%)	887 (13%)
Missing	-	58	35	82
**Ethnicity***				
White British	613 (34%)	102 (47%)	821 (18%)	1527 (23%)
Pakistani Heritage	877 (48%)	80 (37%)	2921 (64%)	3843 (59%)
Other	320 (18%)	36 (17%)	803 (18%)	1148 (18%)
Missing	50	66	177	348
**Depression (PHQ8)**				
None	1187 (66%)	-	2594 (64%)	3774 (64%)
Mild	414 (23%)	-	945 (23%)	1345 (23%)
Moderate	135 (7%)	-	346 (8%)	475 (8%)
Moderately severe	58 (3%)	-	134 (3%)	193 (3%)
Severe	19 (1%)	-	64 (2%)	83 (1%)
Missing	47	284	639	996
**Anxiety (GAD7)**				
None	1280 (75%)	-	2720 (72%)	3991 (73%)
Mild	270 (16%)	-	646 (17%)	908 (17%)
Moderate	100 (6%)	-	224 (6%)	322 (6%)
Severe	67 (4%)	-	166 (4%)	234 (4%)
Missing	143	284	966	1,411

**Note: The ethnicity representativeness is skewed by the BiBBS participants who are ~70% South Asian and had a comparatively low response rate to this survey – see reference 15 for explanation*
