# Supplementary figures and images for: ‘When will this end? Will it end?’ The impact of the March–June 2020 UK COVID-19 lockdown response on mental health: a longitudinal survey of mothers in the Born in Bradford study
Source: BMJ Open. 2022 Jan 11;12(1):e047748. doi: 10.1136/bmjopen-2020-047748 (PMC8753090; doi:10.1136/bmjopen-2020-047748)

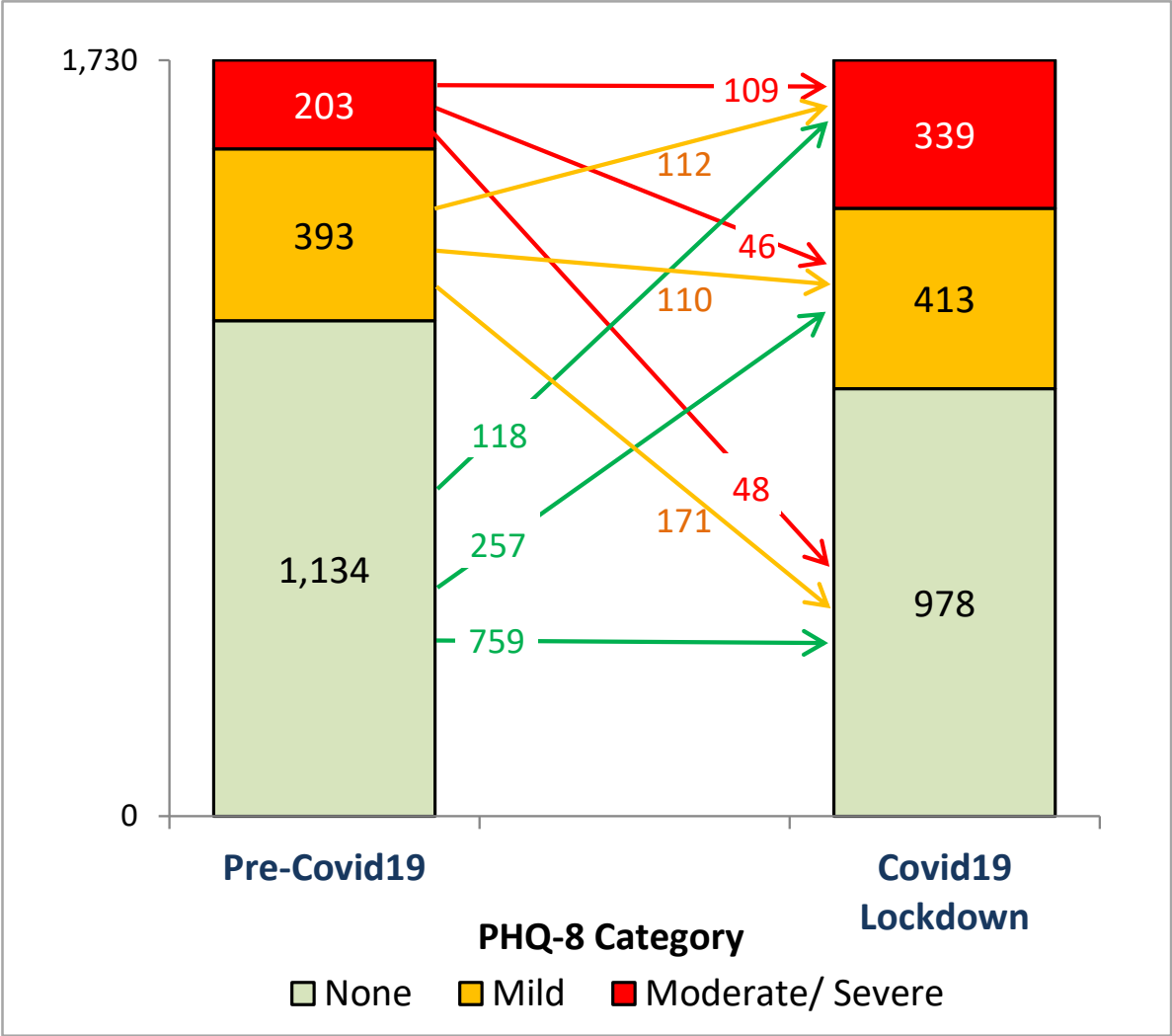

Supplement: Supplementary data [file bmjopen-2020-047748supp002.pdf]

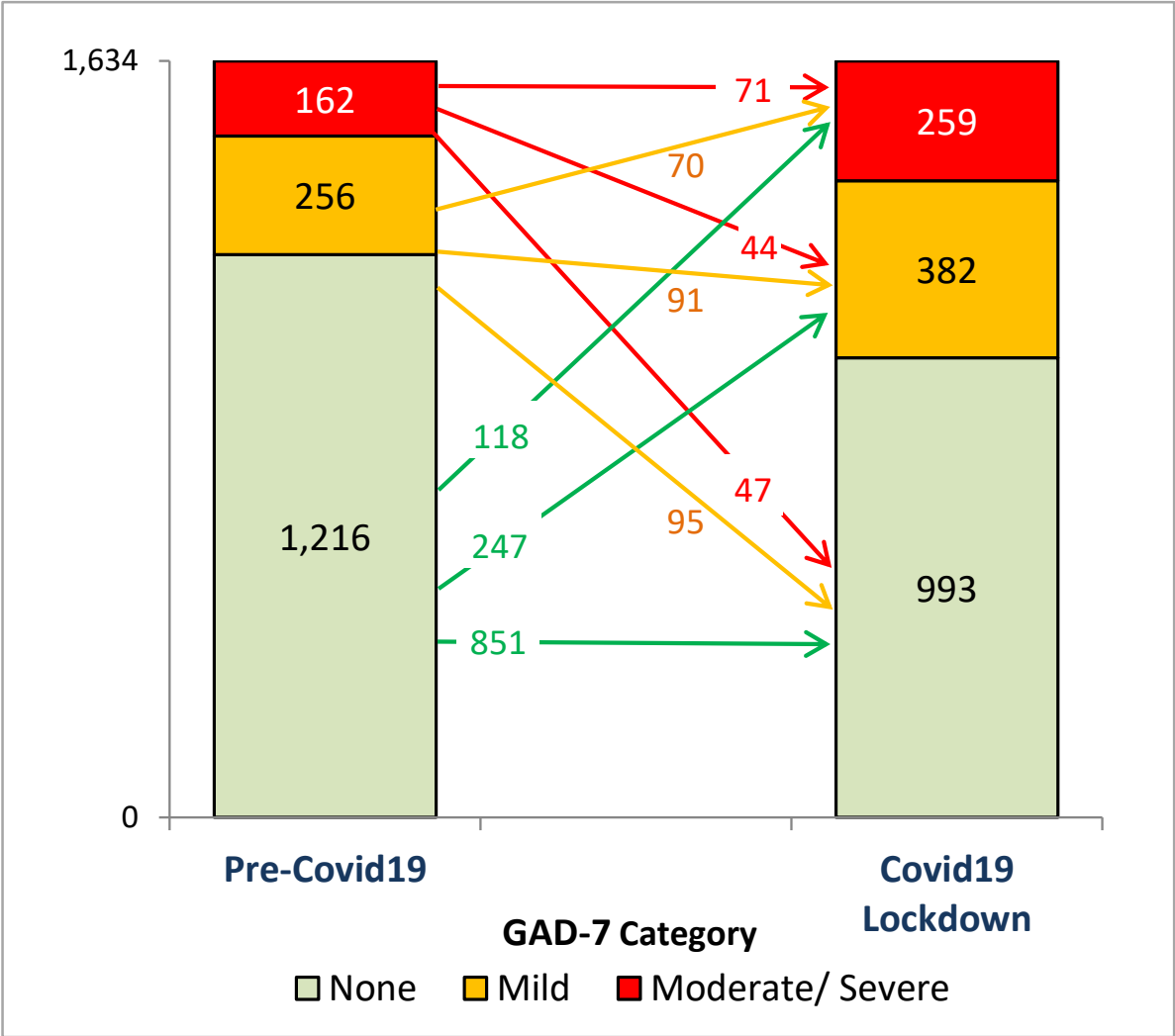

Supplement: Supplementary data [file bmjopen-2020-047748supp003.pdf]
