# Supplementary material for: ‘When will this end? Will it end?’ The impact of the March–June 2020 UK COVID-19 lockdown response on mental health: a longitudinal survey of mothers in the Born in Bradford study
Source: BMJ Open. 2022 Jan 11;12(1):e047748. doi: 10.1136/bmjopen-2020-047748 (PMC8753090; doi:10.1136/bmjopen-2020-047748)
Supplement: Author's manuscript [file bmjopen-2020-047748.draft_revisions.pdf]

BMJ Open

BMJ Open is committed to open peer review. As part of this commitment we make the peer review history of every article we publish publicly available.

When an article is published we post the peer reviewers' comments and the authors' responses online. We also post the versions of the paper that were used during peer review. These are the versions that the peer review comments apply to.

The versions of the paper that follow are the versions that were submitted during the peer review process. They are not the versions of record or the final published versions. They should not be cited or distributed as the published version of this manuscript.

BMJ Open is an open access journal and the full, final, typeset and author-corrected version of record of the manuscript is available on our site with no access controls, subscription charges or pay-per-view fees (http://bmjopen.bmj.com).

If you have any questions on BMJ Open's open peer review process please email info.bmjopen@bmj.com

"When will this end? Will it end?" The impact of the March-June 2020 UK Covid-19 lockdown response on mental health: a longitudinal survey of mothers in the Born in Bradford study.

Journal:	*BMJ Open*
Manuscript ID	bmjopen-2020-047748
Article Type:	Original research
Date Submitted by the Author:	10-Dec-2020
Complete List of Authors:	Dickerson, Josie; Bradford Institute for Health Research<br>Kelly, Brian; Bradford Institute for Health Research<br>Lockyer, Bridget; Bradford Institute for Health Research<br>Bridges, Sally; Bradford Institute for Health Research<br>Cartwright, Christopher; Bradford Institute for Health Research<br>Willan, Kathryn ; Bradford Institute for Health Research<br>Shire, Katy; Bradford Institute for Health Research<br>Crossley, Kirsty; Bradford Institute for Health Research<br>Bryant, Maria; University of York, Health Sciences<br>Siddiqi, Najma; University of York, Health Sciences; Hull York Medical School<br>Lawlor, Deborah; University of Bristol, MRC Integrative Epidemiology Unit at The University of Bristol; University of Bristol Medical School, Population Health Science<br>Wright, John; Bradford Institute for Health Research<br>McEachan, Rosemary; Bradford Institute for Health Research, Bradford, United Kingdom; University of Bradford Faculty of Life Sciences<br>Sheldon, T; Bradford Institute for Health Research; Queen Mary University of London Barts and The London School of Medicine and Dentistry, Institute of Population Health Sciences<br>Pickett, Kate; University of York, Health Sciences
Keywords:	COVID-19, MENTAL HEALTH, Depression & mood disorders < PSYCHIATRY, Anxiety disorders < PSYCHIATRY, PUBLIC HEALTH

SCHOLARONE™
Manuscripts

I, the Submitting Author has the right to grant and does grant on behalf of all authors of the Work (as defined in the below author licence), an exclusive licence and/or a non-exclusive licence for contributions from authors who are: i) UK Crown employees; ii) where BMJ has agreed a CC-BY licence shall apply, and/or iii) in accordance with the terms applicable for US Federal Government officers or employees acting as part of their official duties; on a worldwide, perpetual, irrevocable, royalty-free basis to BMJ Publishing Group Ltd ("BMJ") its licensees and where the relevant Journal is co-owned by BMJ to the co-owners of the Journal, to publish the Work in this journal and any other BMJ products and to exploit all rights, as set out in our licence.

The Submitting Author accepts and understands that any supply made under these terms is made by BMJ to the Submitting Author unless you are acting as an employee on behalf of your employer or a postgraduate student of an affiliated institution which is paying any applicable article publishing charge ("APC") for Open Access articles. Where the Submitting Author wishes to make the Work available on an Open Access basis (and intends to pay the relevant APC), the terms of reuse of such Open Access shall be governed by a Creative Commons licence – details of these licences and which Creative Commons licence will apply to this Work are set out in our licence referred to above.

Other than as permitted in any relevant BMJ Author's Self Archiving Policies, I confirm this Work has not been accepted for publication elsewhere, is not being considered for publication elsewhere and does not duplicate material already published. I confirm all authors consent to publication of this Work and authorise the granting of this licence.

For peer review only

"When will this end? Will it end?" The impact of the March-June 2020 UK Covid-19 lockdown response on mental health: a longitudinal survey of mothers in the Born in Bradford study.

Josie Dickerson[1], Brian Kelly[1], Bridget Lockyer[1], Sally Bridges[1], Christopher Cartwright[1], Kathryn Willan[1], Katy Shire[1], Kirsty Crossley[1], Marian Bryant[1,2] Najma Siddiqi[3], Trevor A Sheldon[1,4], Deborah A Lawlor[,5,6,7], John Wright[1], Rosemary RC McEachan[1,8], Kate E Pickett[1,2] on behalf of the Bradford Institute for Health Research COVID-19 Scientific Advisory Group (https://www.bradfordresearch.nhs.uk/c-sag/).

Corresponding author:

Josie Dickerson, Bradford Institute for Health Research, Bradford Teaching Hospitals NHS Foundation Trust, Bradford Royal Infirmary, Duckworth Lane, Bradford, BD9 6RJ, UK.

Email: josie.dickerson@bthft.nhs.uk

Telephone: 01274 383916

Addresses

[1] Bradford Institute for Health Research, Bradford Teaching Hospitals NHS Foundation Trust, Bradford Royal Infirmary, Duckworth Lane, Bradford, UK.

[2] Department of Health Sciences, University of York, Department of Health Sciences, Seebohm Rowntree Building, University of York, Heslington, York, UK.

[3] The Hull York Medical School, University of York, Heslington York, UK

[4] Institute of Population Health Sciences, Barts and The London School of Medicine and Dentistry, Queen Mary University London, Yvonne Carter Building, 58 Turner Street, London, UK

[5] MRC Integrative Epidemiology Unit at the University of Bristol, Oakfield House, Oakfield Grove, Bristol, , UK

[6] Population Health Science, Bristol Medical School, University of Bristol, UK

[7] Bristol National Institute for Health Research Biomedical Research Centre, UK

[8] Faculty of Life Sciences and Health Studies, University of Bradford, Richmond Rd, Bradford, UK

KEYWORDS

COVID-19; mental health; depression; anxiety; health inequalities; ethnic minorities

WORD COUNT: 4,579

For peer review only - http://bmjopen.bmj.com/site/about/guidelines.xhtml

ABSTRACT

Objectives To determine clinically important change in anxiety and depression from before to during the first UK Covid-19 lockdown and factors related to this change, including ethnic differences.

Design Pre-Covid and lockdown surveys nested within two longitudinal Born in Bradford cohort studies.

Participants 1,860 mothers with a child aged 0-4 or 9-13, 48% Pakistani heritage

Main outcome measures Odds ratios (OR) for a clinically important increase (5 points) in depression (PHQ-8) and anxiety (GAD-7) in unadjusted regression analyses, parsimonious multivariate modelling to explore ethnicity and mental ill health and lived experience of mothers captured in open text questions.

Results Clinically important depression and anxiety increased from 11% to 19%, and 10% to 16% respectively from before to during the first Covid-19 lockdown. Loneliness during lockdown was most strongly associated with increases in depression (OR: 8.37, 95% CIs: 5.70-12.27) and anxiety (8.50, 5.71-12.65), followed by financial insecurity (6.23, 3.96-9.80; 6.03, 3.82-9.51). Other strongly associated variables included food and housing insecurity, a lack of physical activity and a poor partner relationship.

When level of financial insecurity was taken into account, Pakistani heritage mothers were less likely than White British mothers to experience an increase in depression (0.67, 0.51-0.89) and anxiety (0.73, 0.55-0.97).

Responses to open text highlighted a complex inter-play of health anxieties, mental load, loss of social support and coping strategies, and financial insecurity contributing to mental ill health. Positive aspects of lockdown were also reported, including a more relaxed pace of life.

Conclusions Mental ill health has worsened with the Covid-19 lockdown, particularly in those who are lonely, economically insecure and/or of White British ethnicity. Mental health problems may have longer term consequences for public health. Strategies to mitigate adverse impacts of future lockdowns on mental health should focus on those factors we highlight as associated with worsening mental health.

STRENGTHS AND LIMITATIONS OF THIS STUDY

- Two key longitudinal studies have highlighted that the Covid-19 pandemic and lockdowns have had a negative impact on mental health, particular in younger adults, women and those from low socio-economic circumstances, but with participants of predominantly White European ethnicity.

- Mental ill health is more prevalent in people from ethnic minorities and the socially and economically disadvantaged. The Born in Bradford research programme offers a unique opportunity to investigate the impact of Covid-19 lockdown on mental health in a deprived and ethnically diverse population.

- This is a longitudinal study containing linked data collected before the Covid-19 pandemic and at the beginning of the March-June 2020 lockdown which has allowed us to explore change over that time period in a highly ethnically diverse population, the majority of whom live in the most deprived centiles in the UK.

- Respondents in this study were mothers of children aged 0-5 and/or 9-13 which may limit the wider generalizability, though our findings are broadly similar to those from another longitudinal study that included adult men and women, and the deterioration in mental health reported here is similar to that of other UK studies.

- We are not aware of other studies that have explored longitudinal change in mental health from pre- to during- the Covid-19 lock down in a similar ethnically diverse deprived population.

For peer review only - http://bmjopen.bmj.com/site/about/guidelines.xhtml

Introduction

There is growing concern that the first 'lockdown' measures to control the spread of the Covid-19 pandemic (implemented in the UK between March and June 2020)[1,2] have had unintended consequences including an increase in mental ill health. Several studies since the Covid-19 pandemic began have reported high levels of depression and anxiety[3-7] in the UK. However, the majority of these surveys are either cross-sectional or longitudinal *within* the lockdown period (5-7) with only two studies (3, 4) comparing mental health in the pre-Covid-19 period to mental health during the March-June 2020 Covid-19 lockdown. One of those used office of national statistics data and found worsening mental health in younger adults, those who were financially insecure or had a disability[3]. The second, using data from two prospective cohorts, found worsening mental health during, compared to before the lockdown in younger adults, women, those with pre-existing mental and physical health conditions, those living alone and in socio-economic adversity[4]. In these studies most participants were of white European origin and the larger of the two studies was in a relatively affluent population. Mental ill health is more prevalent in people from ethnic minorities and the socially and economically disadvantaged[8,9], but no longitudinal research to date has investigated the impact of Covid-19 lockdown on mental health in these populations[10].

We were able to explore these questions in depth by building on the Born in Bradford research programme which includes two longitudinal birth cohorts of ethnically diverse families living in the city of Bradford, many in deprived circumstances. These cohorts have recent in-depth information on the demographics, socioeconomic status and mental health of participants before the Covid-19 pandemic,[11-13] as well as mental health during the March-June 2020 Covid-19 lockdown[14], and so offer a unique opportunity to assess the impact of Covid-19 and lockdown longitudinally in a deprived and ethnically diverse population.

We explore the data to:

- Describe changes in the prevalence of depression and anxiety in mothers living in Bradford from before the Covid-19 pandemic to during the March-June 2020 Covid-19 lockdown
    - Identify the variables associated with an increased occurrence of mental ill health in this population, to identify vulnerable groups that may need additional support during subsequent lockdowns.
    - To explore mothers' lived experience during the March-June 2020 Covid-19 lockdown by assessing the frequency of worries and concerns relating to mental ill health obtained through free text responses to open questions.

Methods

Study Design

A longitudinal study using data collected at two time points before and during the March-June 2020 Covid-19 lockdown from mothers who participated in one of two prospective birth cohort studies in Bradford: Born in Bradford's Growing Up (BiBGU) study with parents of children currently aged 9-13[11,12] and Born in Bradford's Better Start (BiBBS) with parents of children currently aged 0-4.[13]

Patient and Public Involvement

Born in Bradford is a 'people powered' research study; the local community were consulted to identify key research priorities during the March-June 2020 Covid-19 lockdown. This included consultation with key community groups, seldom-heard communities and local policy and decision makers to ensure that the focus of the research was relevant to local needs. The Covid-19 survey and recruitment approach were tested through our established research advisory groups. The findings of the study were also shared with these groups to enhance interpretation and ensure useful dissemination back to the community[14].

Consent

For peer review only - http://bmjopen.bmj.com/site/about/guidelines.xhtml

Participants had previously consented for their research data, and routinely collected health and education data, to be used for research. For the Covid-19 survey, verbal consent was taken for questionnaires completed over the phone and implied consent was assumed for all questionnaires completed via post or online.

Data Collection

Full details of the data collection and descriptive findings of the March-June 2020 Covid-19 survey can be found elsewhere[14,15]. In summary:

a) Pre-Covid-19 data

Pre-Covid-19 data for BiBGU participants were derived from two sources: a) participant ethnicity and age, collected during pregnancy (2007-2011); [11] b) recent follow-up data on mental health (collected between 24th June 2017 and 12th March 2020).[12] Pre-Covid data for BiBBS participants were taken from data collected during pregnancy (6th January 2016 and 8th February 2020).[13] The median time since most recent pre-Covid data collection was 15 months (range 1 to 35) for BiBGU and 29 months (range 2 to 52) for BiBBS.

b) March-June 2020 Covid-19 lockdown data collection:

The survey was administered between 10th April and 30th June 2020 using a combination of emails, text and phone with a follow-up postal survey. Participants were recruited in their main language wherever possible. Mental health was measured using the PHQ-8[16] for depression and the GAD-7 for anxiety.[17] These are widely used measures of the severity of symptoms of depression and anxiety that have been validated in the general population, [16,17] and in many ethnic minorities including UK residents of Pakistani heritage.[18] Information was also collected on household circumstances; family relationships; social support and loneliness; financial, employment, housing and food insecurity; physical health. This contextual information was captured in self-reported questions administered in the Covid-19 lockdown survey; details of categories used are detailed in Table 1 and our protocol paper[14]. Information on the participants' lived experience during lockdown, including their main worries, challenges and any positive aspects of lockdown were collected using free text questions.

Data Analysis

For PHQ-8 and GAD-7 we employed standard categorisations (0 to 4 no depression, 5 to 9 mild depression, 10-24 moderate-severe depression; 0 to 4 no anxiety, 5 to 9 mild anxiety, 10 to 21 moderate-severe anxiety)[16,17] but collapsed moderate, moderately severe and severe categories to indicate clinically important symptoms of depression and anxiety We also used an increase in PHQ-8 and GAD-7 scores of 5 or more points as an indicator of clinically important change in symptoms. This categorisation was chosen following guidance from previous research and consultation with clinical colleagues[19]. An increase of five points or more would also result in a change in the categorization of symptoms (e.g. from none to mild, mild to moderate/severe and vice versa) whilst also capturing changes in severity within the moderate/severe categories.

Ethnicity was coded using Census 2011 categories and categorised as 'White British', 'Pakistani Heritage' and all other ethnic groups were categorised as 'Other' due to small numbers of a wide range of ethnicities.

A number of categories within variables were collapsed for the regression analysis. This included: Quality of relationship with partner: Average to poor (comprising of 'average', 'poor' and 'very poor'); Loneliness: Not lonely (comprising of: 'none or almost none of the time' and 'some of the time') and Lonely (comprising of: 'Most of the time' and 'All or almost all of the time'); Social support: Easy to get support (comprising of: 'Very easy' and 'easy'); and Not easy to get support (comprising of: 'Very difficult', 'Difficult' and 'Possible'); Food insecurity; Secure (Comprising of' never true' or 'sometimes true' that food didn't last) and insecure (Comprising of 'Often true' that food didn't last). Housing security: Secure (Comprising: 'Strongly Disagree', 'Disagree' and 'Neither disagree or agree' that I worry about being evicted or having my home repossessed) and Insecure (comprising of 'Strongly Agree' or 'Agree').

For peer review only - http://bmjopen.bmj.com/site/about/guidelines.xhtml

Missing data on measures was small for most variables and was not adjusted for in the analyses.

We present results of sample characteristics, including depression and anxiety at pre-Covid-19 and Covid-19 lockdown survey time points. We examined change in depression and anxiety categories between pre-Covid-19 and Covid-19 lockdown surveys to elucidate the patterns of both positive and negative changes. We then modelled the odds ratios associated with an increase in PHQ-8 and GAD-7 scores by five or more points (Model 1). We constructed separate unadjusted models for each outcome and covariate of interest, considering variables that were associated with a clinically important increase in mental ill health in the March-June 2020 Covid-19 lockdown survey.[15] Pre-Covid-19 PHQ-8 and GAD-7 scores were controlled for in each model.

In order to explore the association between ethnicity and a clinically important increase in depression and anxiety in more depth, we conducted parsimonious multivariate modelling exercises. Each model controlled for one variable associated with such an increase in model 1.

All statistical analyses were carried out using Stata 15.[20]

Free text responses in the survey were coded using thematic analysis.[21] The first 100 responses were analysed by one researcher (BL), employing an inductive approach where coding and theme development were driven by the content of the responses. Two codebooks were developed, one for the questions on the three biggest worries and recent challenges during lockdown and another smaller codebook for the question on what had been made more enjoyable and easier during lockdown. The remaining responses were then coded by three different researchers in order to test the strength and validity of the codebooks. Through frequent discussion between the researchers about this process, adjustments were made to the original codebooks so that they were reflective of the total responses. We also used the emergent themes to illuminate the findings from the quantitative analyses.

Ethics

This study was approved by the HRA and Bradford/Leeds research ethics committee (Substantial amendments to: BiBGU 16/YH/0320 and BiBBS 15/YH/0455)

Results

Study Population

Of the 2,043 mothers who responded to the March – June 2020 Covid-19 lockdown survey, 1,860 (91%) had complete surveys and linked data from pre-Covid19 surveys. Of these; 1,316 (71%) were in the BiBGU cohort (with a child aged 9-13) and 544 (29%) were in the BiBBS cohort (with n child aged 0-4). The mothers had a mean age of 37.5 years (SD 6.8). 877 (48%) were of Pakistani heritage, 613 (34%) of White British ethnicity and 320 (18%) of other ethnicities (see Supplemental Table S1). Respondents were representative of the BiB and BiBBS cohorts.[15]

Findings

Moderate / severe depression increased between the pre-Covid-19 and Covid-19 lockdown surveys from 11% (N=212) to 19% (N=349).Rates of mild depression remained similar; while the proportion of those with no depression decreased from 65% (N=1187) to 56% (N=1001), (Table 1). The rates of moderate/severe anxiety increased from 10% (N=167) to 16% (N=289). The prevalence of mild anxiety also increased from 16% (N=270) to 23% (N=408), whilst the proportion of participants with no anxiety fell from 75% (N=1280) to 61% (N=1075), (Table 1).

[Table 1 here]

For peer review only - http://bmjopen.bmj.com/site/about/guidelines.xhtml

For peer review only

Figures 1 and 2 show the change in depression and anxiety categories from the pre-Covid-19 and Covid-19 lockdown surveys. These illustrate that, while rates of depression and anxiety have increased in the study population, some participants' levels of depression and anxiety have improved.

[Figure 1 &2 here]

Table 1 provides the sample characteristics included in the unadjusted regression model. The results from the unadjusted regression models are presented in Table 2. Variables associated with a clinically important increase in depression and anxiety (reported here in order from the largest to smallest odds ratio) were: Loneliness, financial insecurity, relationship quality, food insecurity, housing insecurity, little or no physical activity, lack of social support, unemployment, poor quality housing, pre-Covid-19 financial security, living in a household with someone clinically vulnerable to Covid-19, being a single parent, and having had to self-isolate.
Variables that were not associated with an increase in depression or anxiety were ethnicity, age, being a keyworker, living in a large household and job insecurity.

[Table 2 here]

The results of a multivariate logistic regression model exploring the relationship between ethnicity, financial insecurity, and increased depression and anxiety can be seen in Table 3. When financial security is controlled for, we find that Pakistani heritage respondents are less likely to experience an increase in depression and anxiety compared to White British respondents (0.67, 0.51-0.89) and (0.73, 0.55-0.97). All other variables that were associated with an increase in model 1 were tested in similar parsimonious multivariate logistic regression models. When frequency of physical activity was controlled for, Pakistani mothers are again less likely to experience an increase in depression (0.73, 0.56-0.96), but not anxiety (0.81 (0.61-1.07) compared to White British mothers. There were no significant ethnic differences in any of the other models (see supplementary Table S1-S9).

[Table 3 here]

Free text responses to the question "what are your three biggest worries at the moment" were available for 1799 mothers. Only a small proportion of women identified their mental health issues as one of their biggest worries, N=105 (6%, 95%CI: 5%-7%), slightly greater in White British mothers, N=51 (8%, 95%CI: 6%-11%) than in mothers of Pakistani heritage, N=32 (4%, 95%CI: 3%-5%). More often, mothers reported how wider issues and concerns impacted on their mental health and wellbeing:

Health anxieties about Covid-19: the most commonly reported worry was a fear of bringing the virus home (e.g. from the shops or from their places of work), and themselves or members of their family becoming ill or dying, as well as the fear of what would happen to their children if this did happen to them.

I worry about contracting coronavirus particularly whilst at work and either becoming critically myself unwell or bringing it home to my family

Feeling anxious about the virus and constantly worrying about my kids which 2 of them have health issues and are quite vulnerable

I feel particularly anxious to even step out of the house even for food shopping or taking a walk/exercise

I worry how this will affect my children. I'm terrified they will be separated as I have 2 children with my ex-husband and one with my current. So I haven't been outside in 10 weeks.

Mental load: mothers often reported the mental load of managing work, home-schooling, childcare and domestic tasks, without the break provided by children attending school, nursery or other childcare. Being or feeling stuck inside and unable to move around freely contributed to a sense of suffocation and feeling overwhelmed, and many mothers acknowledged that this was having a detrimental effect on their mental health and self-esteem:

For peer review only - http://bmjopen.bmj.com/site/about/guidelines.xhtml

I'm worried [about] having a nervous breakdown or a panic attack...can't get a break from all the responsibilities and go somewhere for fresh air even

Finding working from home and looking after children very demanding. No time alone. No silence. Surrounded by people and electronics all my waking hours.

I am a keyworker who works five days of week, only while my children are at school. I am becoming mentally drained/exhausted constantly being on duty, it is impacting my mental health.

Homeschooling I am not cut out for this and don't feel good enough

Loss of social support: The loss of social support caused by lockdown, especially for those who didn't live with their partner or were single parents was highlighted as causing loneliness and isolation for some.

My support network was my partner, who I cannot see due to lockdown.

Not able to see my boyfriend - feel isolated and alone once children are in bed with no adult face to face interaction

The isolation and loneliness has been a challenge and having to take care of my child on my own full-time

Being a single parent of a disabled child I rely on my social life/friends for my mental wellbeing and I worry about when it'll be safe to see/hug my immediate family who live locally

Financial and employment insecurity: Household finances and the stress of unemployment or job insecurity related to lockdown measures were also a major worry for women. Many families were in debt, reliant on credit cards and benefits, and in insecure employment before the lockdown measures began and were only just about getting by prior to the pandemic.

I have struggled financially during this time. I would like to not worry about money and bills and shopping and outgoings. I have enough worries being a full time carer.

Worried about the financial impact of covid 19. I am currently furloughed from work but I worry that the virus will have an impact on the business. My husband is self-employed and is not eligible to any funds.

Being unable to switch off: Participants described being frightened of the news reports but unable to switch off, and were wondering when, if ever, things would become normal again:

Worry about my mental health, I know I'm struggling. Worry about the future and how this will affect the country financially. Worry about my children's education & what they've missed out on.

All the bad news on the TV, and the death rate on the News. All the information on the news makes me panic more.

When will this end? Will it end?

A loss of coping strategies: For those who had existing mental health issues before lockdown, the lockdown measures had often taken away their sources of support, their normal routines and methods of coping. In addition, some respondents reported being unable to access mental health services due to Covid-19 and lockdown measures:

Mental health - Exercise at the gym was a coping mechanism, now closed. Can do bits at home but nothing like at the gym. Cannot get any gym equipment - out of stock.

Mental Health - struggling to motivate myself and to keep to a routine. Self-destructive behaviour - drink drugs and binge eating. I am missing my work and colleagues.

Not getting my mental health support since the lockdown. My CPN (Community Psychiatric Nurse) not returning my calls. It has made me a lot worse. I try to talk to my husband so I am not keeping everything inside

Mental health, I have had previous issues in the past and am struggling and don't feel like I can approach my GP at the minute as it isn't an emergency

For peer review only - http://bmjopen.bmj.com/site/about/guidelines.xhtml

Positive aspects of lockdown: Many participants reported positive aspects to changes enforced by the lockdown, commenting that they were getting to spend more quality time with their children, were enjoying a slower pace of life, a more relaxed routine and spending less time driving and commuting.

Life has become a lot more relaxed over the last 3 weeks, no manic mornings trying to get everybody out of the house, time with kids, doing stuff with kids I would normally say 'not now' to. Get to know kids more. More time outside, [doing] jobs in the house that need doing.

Spending more time with my husband and feeling appreciative of each other and having a relaxed day with the children instead of running to school to mosque and then all the extra clubs it's a more relaxed day.

Ramadan is the easiest it has ever been, we are free to make up our sleep and not push ourselves too much, had time to do nice things during Ramadan including having a more peaceful time not having to do school runs, be stressed out, my husband had a chance to take a slower pace to life and not get too stressed.

DISCUSSION

We compared depression and anxiety during the March-June 2020 UK lockdown to pre-Covid19 depression and anxiety data collected in our longitudinal birth cohort studies. We found that clinically important symptoms of depression and anxiety increased from 11% to 19% and from 11% to 16%, respectively. The variables that were most strongly associated with a clinically important increase in depression and anxiety from pre-Covid19 to the March-June 2020 lockdown were loneliness and financial, insecurity. The prevalence of these key variables was high in our families, highlighting the vulnerabilities and risk of poorer outcomes from the Covid-19 pandemic in ethnically diverse and deprived families. White British mothers were found to be more likely to have an increase in depression and anxiety symptoms when variation in financial insecurity was controlled for. White British mothers were also more likely to have an increase in depression but not anxiety when levels of physical activity was controlled for. The free text responses in our Covid-19 lockdown survey allowed us to explore the specificities of poor mental health in more detail highlighting: acute health anxieties; the mental load of managing multiple roles and responsibilities; the loss of social support and other coping strategies; pressures of financial and employment insecurity; and being unable to switch off from the pandemic. These responses highlight the complexities of how the March-June 2020 lockdown may have impacted on mental health, and also how some of the positive experiences may have protected some mothers from mental ill health at this time.

This is a longitudinal study containing linked data collected before the Covid-19 pandemic and at the beginning of the March-June 2020 lockdown which has allowed us to explore change over that time period. It also provides findings from a highly ethnically diverse population, the majority of whom live in the most deprived centiles in the UK. We are not aware of other studies that have explored longitudinal change in mental health from pre- to during- the Covid-19 lock down in a similar ethnically diverse deprived population

Respondents were mothers of children aged 0-5 and/or 9-13 which may limit the wider generalizability, though our findings are broadly similar to those from a previous longitudinal study of two UKC cohorts that included adult men and women (not all of whom were mothers), and found the increased risk of poor mental health in lockdown to be greater in women[4]. Our pre-COVID19 measures were taken from data collected over the past 4 years, so we cannot with confidence attribute all changes to the pandemic and the lockdown. For example, it is possible that some of the difference reflects age related change in the women and/or their children over time. It is also possible that we have underestimated some of the adverse impact of lockdown as a significant percentage of the participants were pregnant at baseline, which itself is associated with raised levels of depression. However, the deterioration in mental health is large and is similar to that of the ONS study which found that depressive symptoms in the general population of the UK doubled to 19.6% in June 2020 compared to the same time period in 2019.[3]

It is possible that our results are influenced by selection bias given that 28% of those invited to participate in the Covid-19 survey responded. However these participants were representative of the BiB and BiBBS cohorts[15], and have demonstrated a wide variability in most characteristics (Table 1).

We will continue to follow our families over time so that we can look at trajectories of change overtime, including during any future national or regional lockdowns. At time of writing (November 2020), the UK is under a further nation-wide lockdown, following a period of reduced restrictions during summer 2020 and

For peer review only - http://bmjopen.bmj.com/site/about/guidelines.xhtml

tighter regional restrictions in the more deprived cities of Northern England, including Bradford, though the autumn. The impact of further and longer periods of restrictions and lockdowns will be a focus of our study moving forwards.

The increase in depression and anxiety in White British mothers relative to Pakistani mothers, once financial insecurity and physical activity were controlled, is of interest. Financial insecurity and low levels of physical activity are higher in our Pakistani families, but the resilience to mental ill health appears to be greater in Pakistani mothers compared to White British mothers once these variables are accounted for. We have previously reported that White British mothers are more likely to have their mental ill health identified by health professionals than South Asian women but that both White British and South Asian women are equally likely to disclose symptoms in self-report research questionnaires as used here.[22,23] It is possible that differences in family structure and culture might provide more support in times of adversity, as we have found in the case of food insecurity previously[24,25]. This hypothesis warrants further investigation, and longitudinal qualitative research would add real depth to understanding this difference.

Our results highlight the public health impact of lockdown on mental health, particularly in those who are lonely and economically insecure and of White British ethnicity. Mental health problems are in general less visible than physical symptoms and in particular the physical symptoms related to Covid-19, including the acutely ill patients in ICUs, but may have more significant longer-term consequences.

Government and local councils should consider policies that permit 'social bubbles' that can be implemented to reduce loneliness for those at risk, and for voluntary services to continue to focus support to those who are lonely/isolated. Policy and decision makers should also make provision for the continuing need to support and protect vulnerable families from financial, food and housing insecurity, all of which were associated with poor mental health in this study. These actions will be increasingly important through further regional and national lockdowns.

For peer review only

DISSEMINATION DECLARATION

The findings of the study were also shared with our community research advisory groups to enhance interpretation. We will share our findings with all participants using a combination of social media, infographics posted to participants and community dissemination events

DATA (AND SOFTWARE) AVAILABILITY

Born in Bradford offer open access to their data resources. Available data and procedures to access this can be found at: https://borninbradford.nhs.uk/research/how-to-access-data/_

AUTHOR CONTRIBUTIONS

JD was involved in the concept and study design, design of the data collection tools, overall supervision of the study and data collection, wrote the statistical analysis plan, and drafted and revised the paper. She is guarantor. BK was involved in the design of data collection tools, wrote the statistical analysis plan, conducted all statistical analyses, and drafted and revised the paper. BL devised the coding framework for free text responses, analysed the data and drafted and revised the paper. SB was involved in the concept and study design, design of the data collection tools, supervision of the study and data collection, and reviewing and revising the paper. CC was involved in the concept and study design, and reviewing and revising the paper. KW was involved in design of the data collection tools, data curation, analysis, reviewing and revising the paper. KS was involved in the study design, design of the data collection tools, supervision of the study and data collection, and reviewing and revising the paper. KC was involved in the study design, design of the data collection tools, supervision of the study and data collection, data curation and reviewing and revising the paper. MB involved in the study design, and drafted and revised the paper. NS advised on the clinical significance of changes in mental ill health, revising the statistical analysis plan, and drafted and revised the paper. TS was involved in the concept and study design, supervision and support to junior members of the team and drafted and revised the paper. DAL was involved in the concept and study design, design of the data collection tools, and drafted and revised the paper. JW was involved in the concept and study design, design of the data collection tools, supervision and support to junior members of the team and drafted and revised the paper. RM was involved in the concept and study design, design of the data collection tools, revised the statistical analysis plan, and drafted and revised the paper. KP was involved in the concept and study design, design of the data collection tools, revised the statistical analysis plan, supervision and support to junior members of the team and drafted and revised the paper.

COMPETING INTERESTS

All authors have completed the ICMJE uniform disclosure form at www.icmje.org/coi_disclosure.pdf and declare: all authors had financial support from funding bodies listed below for the submitted work; no financial relationships with any organisations that might have an interest in the submitted work in the previous three years; no other relationships or activities that could appear to have influenced the submitted work.

FUNDING INFORMATION

This study was funded by The Health foundation Covid-19 Award (2301201), with further contributions from: A Wellcome Trust infrastructure grant (WT101597MA); A joint grant from the UK Medical Research Council (MRC) and UK Economic and Social Science Research Council (ESRC): MR/N024391/1; The National Institute for Health Research under its Applied Research Collaboration Yorkshire and Humber (NIHR200166); ActEarly UK Prevention Research Partnership Consortium (MR/S037527/1); Better Start Bradford through The National Lottery Community Fund. DAL works in a unit that received support from the University of Bristol and UK Medical Research Council (MC_UU_00011/6) and is a UK National Institute of Health Research senior Investigator (NF-0616-10102)

For peer review only - http://bmjopen.bmj.com/site/about/guidelines.xhtml

ACKNOWLEDGEMENTS

Born in Bradford is only possible because of the enthusiasm and commitment of the children and parents in BiB. We are grateful to all the participants, parent governors and community research advisory group members, health professionals and researchers who have made Born in Bradford happen.

This report is independent research partly funded by the National Institute for Health Research Yorkshire and Humber ARC. The views expressed in this publication are those of the authors and not necessarily those of the National Institute for Health Research or the Department of Health and Social Care.

TRANSPARENCY STATEMENT

The lead author (the manuscript's guarantor) affirms that the manuscript is an honest, accurate, and transparent account of the study being reported; that no important aspects of the study have been omitted; and that any discrepancies from the study as originally planned (and, if relevant, registered) have been explained.

For peer review only

1. REFERENCES

1. Coronavirus (Covid-19) UK Government Response. https://www.gov.uk/coronavirus. Accessed 27.11.20

2. Number of coronavirus (COVID-19) cases and risk in the UK. UK Government Guidance. https://www.gov.uk/guidance/coronavirus-covid-19-information-for-the-public . Accessed 27.11.20

3. Office of National Statistics (2020). Coronavirus and depression in adults, Great Britain: June 2020. https://www.ons.gov.uk/peoplepopulationandcommunity/wellbeing/articles/coronavirusanddepressioninadultsgreatbritain/june2020. Accessed 27.11.20

4. Kwong ASF, Pearson RM, Adams MJ, et al., Mental health during the COVID-19 pandemic in two longitudinal UK population cohorts. *MedRxiv* 2020; 20133116. doi: 10.1101/2020.06.16.20133116

5. Pierce M, Hope H, Ford T, et al., Mental health before and during the COVID-19 pandemic: a longitudinal probability sample survey of the UK population. *Lancet Psychiatry* 2020, https://doi.org/10.1016/S2215-0366(20)30308-4

6. Mind (2020). The mental health emergency: how has the coronavirus pandemic impacted our mental health? London: Mind. Available at: mind.org.uk. Accessed 27.11.20

7. Fancourt D, Steptoe A, Bu F. Trajectories of depression and anxiety during enforced isolation due to COVID-19: longitudinal analyses of 59,318 adults in the UK with and without diagnosed mental illness. medRxiv 2020. **doi:** https://doi.org/10.1101/2020.06.03.20120923

8. Bignall T, Jeraj S, Helsby E, Butt J. Racial disparities in mental health: Literature and evidence review. Race Equality Foundation 2019. https://raceequalityfoundation.org.uk/wp-content/uploads/2020/03/mental-health-report-v5-2.pdf. Accessed 27.11.20

9. Grey T, Sewell H, Shapiro G, Ashraf F. Mental Health Inequalities Facing U.K. Minority Ethnic Populations Causal Factors and Solutions. J Psychological Issues in Organizational Culture. 2013: 3 (S1).

10. Public Health England. COVID-19: mental health and wellbeing surveillance report: https://www.gov.uk/government/publications/covid-19-mental-health-and-wellbeing-surveillance-report/2-important-findings-so-far#which-population-groups-appear-to-be-disproportionally-affected. Accessed 27.11.20

11. Wright J, Raynor P, Tuffnell D, et al. Cohort Profile: The Born in Bradford multi-ethnic family cohort study. *International Journal of Epidemiology*, Volume 42, Issue 4, August 2013, Pages 978–991, https://doi.org/10.1093/ije/dys112

12. Bird PK, McEachan RRC, Mon-Williams M, Small N, West J, Whincup P, Wright J, Andrews E, Barber SE, Hill LJB *et al*: Growing up in Bradford: protocol for the age 7–11 follow up of the Born in Bradford birth cohort. *BMC Public Health* 2019, 19(1):939.

13. Dickerson J, Bird PK, McEachan RRC, Pickett KE, Waiblinger D, Uphoff E, Mason D, Bryant M, Bywater T, Bowyer-Crane C *et al*: Born in Bradford's Better Start: an experimental birth cohort study to evaluate the impact of early life interventions. *BMC Public Health* 2016, 16(1):711.

14. McEachan RRC, Dickerson J, Bridges S *et al.* The Born in Bradford COVID-19 Research Study: Protocol for an adaptive mixed methods research study to gather actionable intelligence on the impact of COVID-19 on health inequalities amongst families living in Bradford [version 1; peer review: 3 approved]. *Wellcome Open Res* 2020, **5**:191 (https://doi.org/10.12688/wellcomeopenres.16129.1)

15. Dickerson J, Kelly B, Lockyer B *et al.* Experiences of lockdown during the Covid-19 pandemic: descriptive findings from a survey of families in the Born in Bradford study [version 1; peer review: 1 approved]. *Wellcome Open Res* 2020, **5**:228 (https://doi.org/10.12688/wellcomeopenres.16317.1)

16. Kroenke K, Strine TW, Spitzer RL, Williams JBW, Berry JT, Mokdad AH: The PHQ-8 as a measure of current depression in the general population. *Journal of Affective Disorders* 2009, 114(1):163-173.

For peer review only - http://bmjopen.bmj.com/site/about/guidelines.xhtml

17. Löwe B, Decker O, Müller S, Brähler E, Schellberg D, Herzog W, Herzberg PY: Validation and Standardization of the Generalized Anxiety Disorder Screener (GAD-7) in the General Population. *Medical Care* 2008, 46(3):266-274.

18. Husain N, Waheed W, Tomenson B, Creed F. The validation of personal health questionnaire amongst people of Pakistani family origin living in the United Kingdom. Journal of Affective Disorders 2007, 97: 261-264.

19. McMillan D, Gilbody S, Richards D. Defining successful treatment outcome in depression using the PHQ-9: A comparison of methods. J. Affective Disorders. 2010: 127; 122–129

20. StataCorp. 2017. Stata Statistical Software: Release 15. College Station, TX: StataCorp LLC.

21. Braun V, Clarke V: Using thematic analysis in psychology. *Qualitative Research in Psychology* 2006, 3(2):77-101.

22. Prady SL, Pickett KE, Petherick ES, Gilbody S, Croudace T, Mason D, Sheldon TA, Wright J. (2016). Evaluation of ethnic disparities in detection of depression and anxiety in primary care during the maternal period: combined analysis of routine and cohort data. The British journal of psychiatry, 208(5), 453-61. DOI 10.1192/bjp.bp.114.158832. PMID 26795424

23. Prady SL, Pickett KE, Gilbody S, Petherick ES, Mason D, Sheldon TA, and Wright J (2016). Variation and ethnic inequalities in treatment of common mental disorders before, during and after pregnancy: combined analysis of routine and research data in the Born in Bradford cohort. BMC Psychiatry, 16(99), DOI 10.1186/s12888-016-0805-x PMID 27071711

24. Power M, Uphoff N, Stewart-Knox B et al., Food insecurity and socio-demographic characteristics in two UK ethnic groups: an analysis of women in the Born in Bradford cohort. *Journal of Public Health*, 2017. **40**(1): p. 32-40.

25. Yang T.C, Sahota P, Pickett K, Bryant M. Association of food security status with overweight and dietary intake: exploration of White British and Pakistani-origin families in the Born in Bradford cohort. *Nutrition Journal*, 2018. **17**(1): p. 48.

For peer review only

For peer review only - http://bmjopen.bmj.com/site/about/guidelines.xhtml

FIGURE LEGENDS

Figure 1: PHQ-8 at pre-Covid19 and Covid19 lockdown survey with change between categories

Note: This graph presents findings from participants who had complete pre-Covid19 and Covid19 lockdown PHQ-8 scores (N=1730)

Figure 2: GAD-7 at pre-Covid19 and Covid19 lockdown survey with change between categories

Note: This graph presents findings from participants who had complete pre-Covid19 and Covid19 lockdown GAD-7 scores (N=1634)

For peer review only

Table 1: Sample characteristics

Sample characteristics	N	Percentage (95% CI)
**Depression category at pre-Covid19 survey (from PHQ-8 score)**		
None (PHQ-8 score 0 to 4)	1187	65% (63%-68%)
Mild (PHQ-8 score 5 to 9)	414	23% (21%-25%)
Moderate (PHQ-8 score 10 to 14)	135	7% (6%-9%)
Moderately severe/ severe (PHQ-8 score 15 to 24)	77	4% (3%-5%)
Missing	47	
Total	1860	100%
**Depression category at lockdown survey (from PHQ8 score)**		
None (PHQ-8 score 0 to 4)	1001	56% (54%-59%)
Mild (PHQ-8 score 5 to 9)	425	24% (22%-26%)
Moderate (PHQ-8 score 10 to 14)	199	11% (10%-13%)
Moderately severe/ severe (PHQ-8 score 15 to 24)	150	8% (7%-10%)
Missing	85	
Total	1860	100%
**Anxiety category at pre-Covid19 survey (from GAD7 score)**		
None (GAD-7 score 0 to 4)	1280	75% (72%-77%)
Mild (GAD-7 score 5 to 9)	270	16% (14%-18%)
Moderate (GAD-7 score 10 to 14)	100	6% (5%-7%)
Severe (GAD-7 score 15 to 21)	67	4% (3%-5%)
Missing	143	
Total	1860	100%
**Anxiety category at lockdown survey (from GAD7 score)**		
None (GAD-7 score 0 to 4)	1075	61% (58%-63%)
Mild (GAD-7 score 5 to 9)	408	23% (21%-25%)
Moderate (GAD-7 score 10 to 14)	165	9% (8%-11%)
Severe (GAD-7 score 15 to 21)	124	7% (6%-8%)
Missing	88	
Total	1860	100%
**Ethnicity**		
White British	613	34% (32%-36%)
Pakistani heritage	877	48% (46%-51%)
Other	320	18% (16%-20%)
Missing	50	
Total	1860	100%
**Age at lockdown survey**		
Under 30 yrs.	224	12% (11%-14%)
30 to 34 yrs.	396	21% (19%-23%)
35 to 39 yrs.	516	28% (26%-30%)
40 to 44 yrs.	423	23% (21%-25%)
45 yrs. plus	301	16% (15%-18%)
Total	1860	100%
**Financial security at pre-Covid19 survey**		
Living comfortably	587	33% (30%-35%)
Doing alright	750	42% (39%-44%)
Just about getting by	342	19% (17%-21%)

For peer review only

For peer review only - http://bmjopen.bmj.com/site/about/guidelines.xhtml

Finding it quite/ very difficult	125	7% (6%-8%)
Missing	56	
Total	1860	100%
**Financial security at lockdown survey**		
Living comfortably	354	20% (18%-22%)
Doing alright	766	42% (40%-45%)
Just about getting by	462	26% (24%-28%)
Finding it quite/ very difficult	223	12% (11%-14%)
Missing	55	
Total	1860	100%
**Change in financial status (compared to 3 months prior, as asked in lockdown survey)**		
Better off	166	10% (8%-11%)
About the same	989	58% (55%-60%)
Worse off	562	33% (31%-35%)
Missing	143	
Total	1860	100%
**Index of Multiple Deprivation 2019 Quintile**		
1: Most Deprived	1,211	65% (63%-67%)
2	390	21% (19%-23%)
3	139	7% (6%-9%)
4	88	5% (4%-6%)
5: Least Deprived	29	2% (1%-2%)
Missing	3	
Total	1860	100%
**Employment status of main earner**		
Employed - working from home	393	22% (20%-24%)
Employed: going into work	567	32% (30%-34%)
On furlough	271	15% (14%-17%)
Self-employed: working	147	8% (7%-10%)
Self-employed: not working	200	11% (10%-13%)
Unemployed	208	12% (10%-13%)
Missing	74	
Total	1860	100%
**Whether respondent is a keyworker**		
No	1,259	68% (66%-71%)
Yes	579	32% (29%-34%)
Missing	22	
Total	1860	100%
**Whether anyone in household is clinically vulnerable to Covid-19**		
No	1,419	77% (75%-79%)
Yes	426	23% (21%-25%)
Missing	15	
Total	1860	100%
**Whether anyone in household is currently/ has previously self-isolated**		
No	1,331	72% (70%-74%)
Yes	508	28% (26%-30%)
Missing	21	
Total	1860	100%

For peer review only - http://bmjopen.bmj.com/site/about/guidelines.xhtml

**Number of children in household**		
One	387	21% (19%-23%)
Two	699	38% (35%-40%)
Three	451	24% (22%-26%)
Four or more	323	17% (16%-19%)
Total	1860	100%
**Total household size**		
Two/ Three	321	17% (16%-19%)
Four/ Five	943	51% (49%-53%)
Six or more	583	32% (29%-34%)
Missing	13	
Total	1860	100%
**Whether single parent**		
No	1,604	88% (86%-89%)
Yes	222	12% (11%-14%)
Missing	34	
Total	1860	100%
**Quality of relationship with partner (for those married or in a relationship)**		
Excellent	783	51% (48%-53%)
Good	598	39% (36%-41%)
Average	129	8% (7%-10%)
Poor	23	1% (1%-2%)
Very poor	10	1% (0%-1%)
Missing	61	
Total	1604	100%
**Loneliness: How often felt lonely in the past week**		
None, or almost none of the time	998	57% (55%-60%)
Some of the time	555	32% (30%-34%)
Most of the time	131	8% (6%-9%)
All, or almost all of the time	56	3% (2%-4%)
Missing	120	
Total	1860	100%
**Social support: how easy to get help from friends/ neighbours if needed**		
Very difficult	101	6% (5%-7%)
Difficult	146	8% (7%-9%)
Possible	529	29% (27%-31%)
Easy	529	29% (27%-31%)
Very easy	513	28% (26%-30%)
Missing	42	
Total	1860	100%
**Food security: How often is it true that food does not last**		
Never	1,379	79% (77%-81%)
Sometimes	279	16% (14%-18%)
Often	91	5% (4%-6%)
Missing	111	
Total	1860	100%
**Housing security: Whether worried about eviction/ repossession**		
Strongly disagree or disagree	1,250	71% (68%-73%)
Neither	328	19% (17%-20%)

For peer review only

Strongly agree/ agree	194	11% (10%-12%)
Missing	88	
Total	1860	100%
**Job security: Whether expect main earner to still have job in 12 months (excluding those currently unemployed)**		
No	77	5% (4%-6%)
Yes	979	61% (59%-64%)
Don't know	537	34% (31%-36%)
Missing	59	
Total	1652	100%
**Housing conditions: Whether damp in house**		
No	1,307	71% (69%-73%)
Yes	534	29% (27%-31%)
Missing	19	
Total	1860	100%
**Frequency of physical activity currently**		
Never	239	13% (12%-15%)
1 or 2 days a week	489	27% (25%-29%)
Most days	516	28% (26%-30%)
Every day	586	32% (30%-34%)
Missing	30	
Total	1860	100%
**Change in frequency of physical activity since lockdown**		
Less	766	48% (46%-51%)
About the	408	26% (24%-28%)
More	419	26% (24%-29%)
Missing	267	
Total	1860	100%

For peer review only

Table 2: Odds ratios (with 95% CI) from unadjusted logistic regression models for increase of 5 points or more between pre-Covid19 and Covid-19 lockdown survey

Covariate	PHQ-8 increase >= 5			GAD-7 increase >= 5		
	Odds Ratio	Low 95% CI	High 95% CI	Odds Ratio	Low 95% CI	High 95% CI
**Cohort Source (Reference: BiBGU)**						
BiBBS	0.84	0.64	1.10	0.84	0.63	1.12
**Ethnicity (Reference: White British)**						
Pakistani heritage	0.88	0.68	1.15	0.94	0.72	1.23
Other	0.94	0.67	1.32	0.85	0.59	1.21
**Age group (Reference: Under 30 years)**						
30 to 34 years	1.01	0.66	1.54	0.90	0.57	1.42
35 to 39 years	0.89	0.59	1.35	0.99	0.64	1.53
40 to 44 years	1.04	0.69	1.58	0.88	0.57	1.38
45 years plus	0.92	0.59	1.45	1.02	0.64	1.63
**Financial security at pre-Covid19 survey (Reference: Living comfortably)**						
Doing alright	1.07	0.81	1.42	1.19	0.89	1.59
Just about getting by	1.72	1.23	2.40	1.66	1.17	2.35
Finding it quite/ very difficult	1.70	1.03	2.80	2.39	1.44	3.98
**Financial security at lockdown survey (Reference: Living comfortably)**						
Doing alright	1.74	1.18	2.56	1.47	1.00	2.17
Just about getting by	3.52	2.36	5.26	3.20	2.15	4.76
Finding it quite/ very difficult	6.23	3.96	9.80	6.03	3.82	9.51
**Change in financial status compared to 3 months ago (Reference: Better off)**						
About the same	0.65	0.43	0.98	0.88	0.55	1.40
Worse off	1.36	0.89	2.01	1.99	1.25	3.19
**Index of Multiple Deprivation 2019 Quintile (Reference: 1 Most deprived quintile)**						
2	0.90	0.67	1.21	1.22	0.92	1.63
3	0.91	0.58	1.41	0.87	0.55	1.39
4	0.76	0.43	1.34	0.98	0.57	1.68
5 Least deprived quintile	0.85	0.34	2.13	0.99	0.39	2.48
**Employment status of main earner (Reference: Employed and working from home)**						
Employed: going into work	1.18	0.84	1.65	1.20	0.85	1.69
On furlough	1.19	0.79	1.79	1.29	0.85	1.94
Self-employed: working	0.90	0.53	1.51	0.92	0.55	1.57
Self-employed: not working	1.49	0.97	2.27	1.31	0.84	2.04
Unemployed	2.03	1.34	3.09	2.23	1.44	3.44
**Whether respondent is a keyworker (Reference: No)**						

For peer review only - http://bmjopen.bmj.com/site/about/guidelines.xhtml

Yes	0.99	0.77	1.27	1.05	0.82	1.35
**Whether anyone in household is clinically vulnerable to Covid-19 (Reference: No)**						
Yes	1.68	1.29	2.19	1.53	1.17	2.01
**Whether anyone in household is currently/ has previously self-isolated (Reference: No)**						
Yes	1.59	1.24	2.04	1.48	1.14	1.92
**Number of children in household (Reference: One)**						
Two	1.20	0.87	1.67	1.05	0.76	1.45
Three	1.39	0.98	1.98	1.20	0.84	1.71
Four or more	1.12	0.76	1.65	0.93	0.62	1.39
**Total household size (Reference: Less than four)**						
Four or Five	0.73	0.53	0.99	0.95	0.69	1.32
Six or more	0.72	0.51	1.00	0.90	0.63	1.29
**Whether single parent (Reference: No)**						
Yes	1.66	1.18	2.33	1.28	0.89	1.85
**Quality of relationship with partner (Reference: Excellent)**						
Good	1.93	1.45	2.57	1.82	1.35	2.44
Average to poor	3.64	2.44	5.43	5.07	3.37	7.62
**Loneliness (Reference: not lonely most/ all of the time)**						
Lonely most/ all of the time	8.37	5.70	12.27	8.50	5.71	12.65
**Social support (Reference: Easy to get help from friends/ neighbours if needed)**						
Not easy to get help	2.25	1.78	2.86	2.13	1.67	2.73
**Food security (Reference: Secure - Never or sometimes true that food does not last)**						
Insecure - Often true that food does not last	3.33	2.09	5.28	3.46	2.15	5.58
**Housing security (Reference: Secure - Not worried about eviction/ repossession)**						
Insecure - Worried about eviction/ repossession	3.29	2.36	4.58	3.00	2.11	4.25
**Job security: whether expect main earner to still have job in 12 months (Reference: Yes)**						
No	0.84	0.49	1.43	0.96	0.56	1.64
**Housing conditions (Reference: No damp in house)**						
Damp in house	1.85	1.45	2.38	1.57	1.21	2.03
**Frequency of physical activity (Reference: Every day)**						
Most days	1.58	1.15	2.18	1.67	1.21	2.31
1 or 2 days a week	1.93	1.40	2.67	1.67	1.20	2.31
Never	3.13	2.15	4.56	2.55	1.72	3.78

For peer review only - http://bmjopen.bmj.com/site/about/guidelines.xhtml

**Change in frequency of physical activity since lockdown (Reference: More than before)**						
About the same	0.66	0.45	0.97	0.82	0.56	1.21
Less than before	1.27	0.94	1.72	1.43	1.04	1.95

For peer review only

For peer review only - http://bmjopen.bmj.com/site/about/guidelines.xhtml

Table 3a: Odds ratios (with 95% CI) from univariate and multivariate logistic regression models for increase in PHQ-8 of 5 points or more between pre-Covid19 and Covid-19 lockdown survey

PHQ-8 increase >= 5	Univariate model			Multivariate model		
**Covariate**	**Odds Ratio**	**Low 95% CI**	**High 95% CI**	**Odds Ratio**	**Low 95% CI**	**High 95% CI**
**Ethnicity (Reference: White British)**						
Pakistani heritage	0.88	0.68	1.15	0.67	0.51	0.89
Other	0.94	0.67	1.32	0.78	0.55	1.12
**Financial security at lockdown survey (Reference: Living comfortably)**						
Doing alright				1.90	1.27	2.84
Just about getting by				3.92	2.58	5.97
Finding it quite/ very difficult				7.47	4.64	12.03

Models also controls for baseline PHQ-8 score

Table 3b: Odds ratios (with 95% CI) from univariate and multivariate logistic regression models for increase in GAD-7 of 5 points or more between pre-Covid19 and Covid-19 lockdown survey

GAD-7 increase >= 5	Univariate model			Multivariate model		
**Covariate**	**Odds Ratio**	**Low 95% CI**	**High 95% CI**	**Odds Ratio**	**Low 95% CI**	**High 95% CI**
**Ethnicity (Reference: White British)**						
Pakistani heritage	0.94	0.72	1.23	0.73	0.55	0.97
Other	0.85	0.59	1.21	0.74	0.51	1.08
**Financial security at lockdown survey (Reference: Living comfortably)**						
Doing alright				1.51	1.02	2.24
Just about getting by				3.33	2.22	5.00
Finding it quite/ very difficult				6.62	4.13	10.61

Models also controls for baseline GAD-7 score

For peer review only - http://bmjopen.bmj.com/site/about/guidelines.xhtml

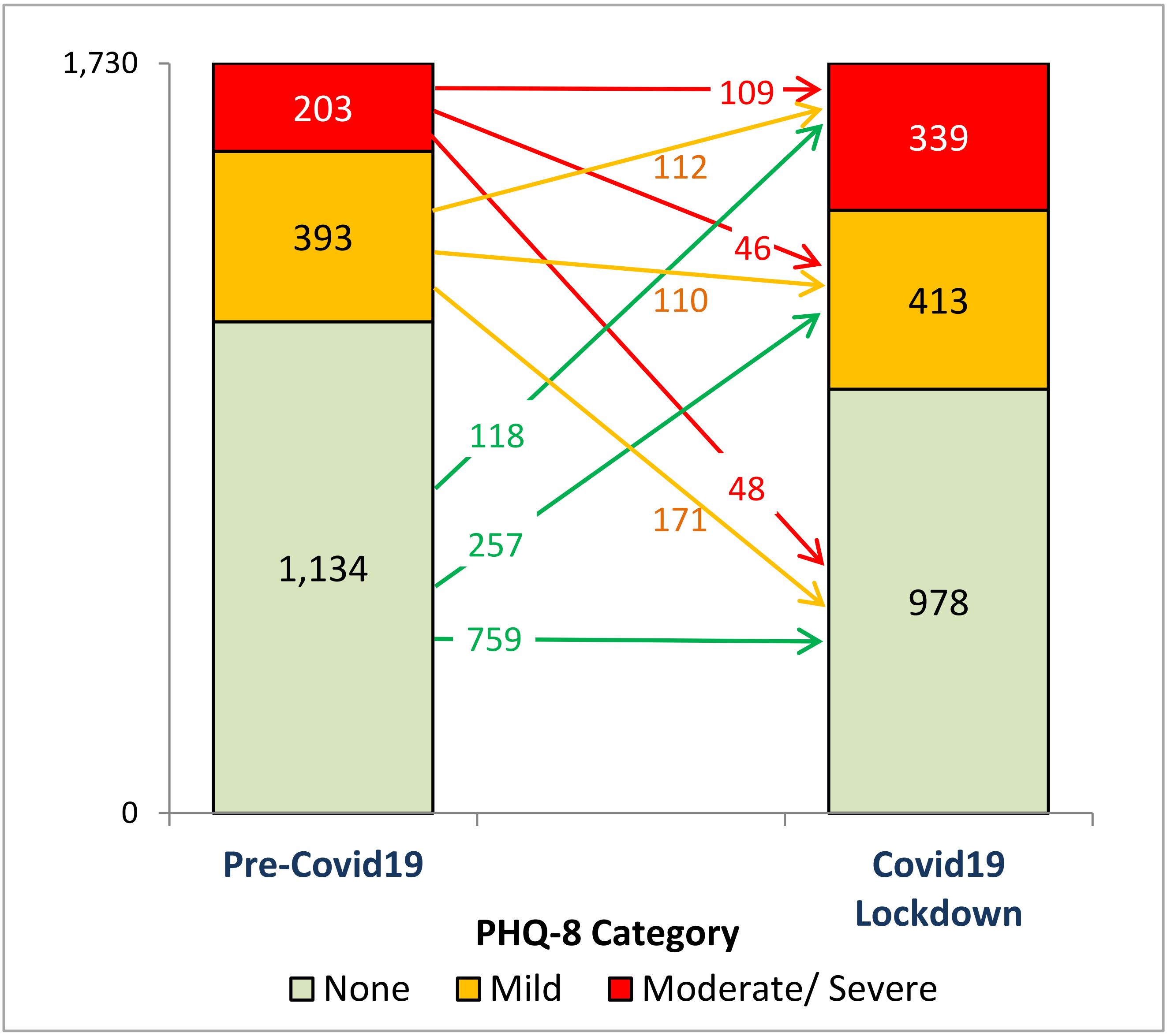
1,730
0
203
393
1,134
339
413
978
109
112
46
110
118
48
171
257
759
Pre-Covid19
Covid19 Lockdown
PHQ-8 Category
None
Mild
Moderate/ Severe

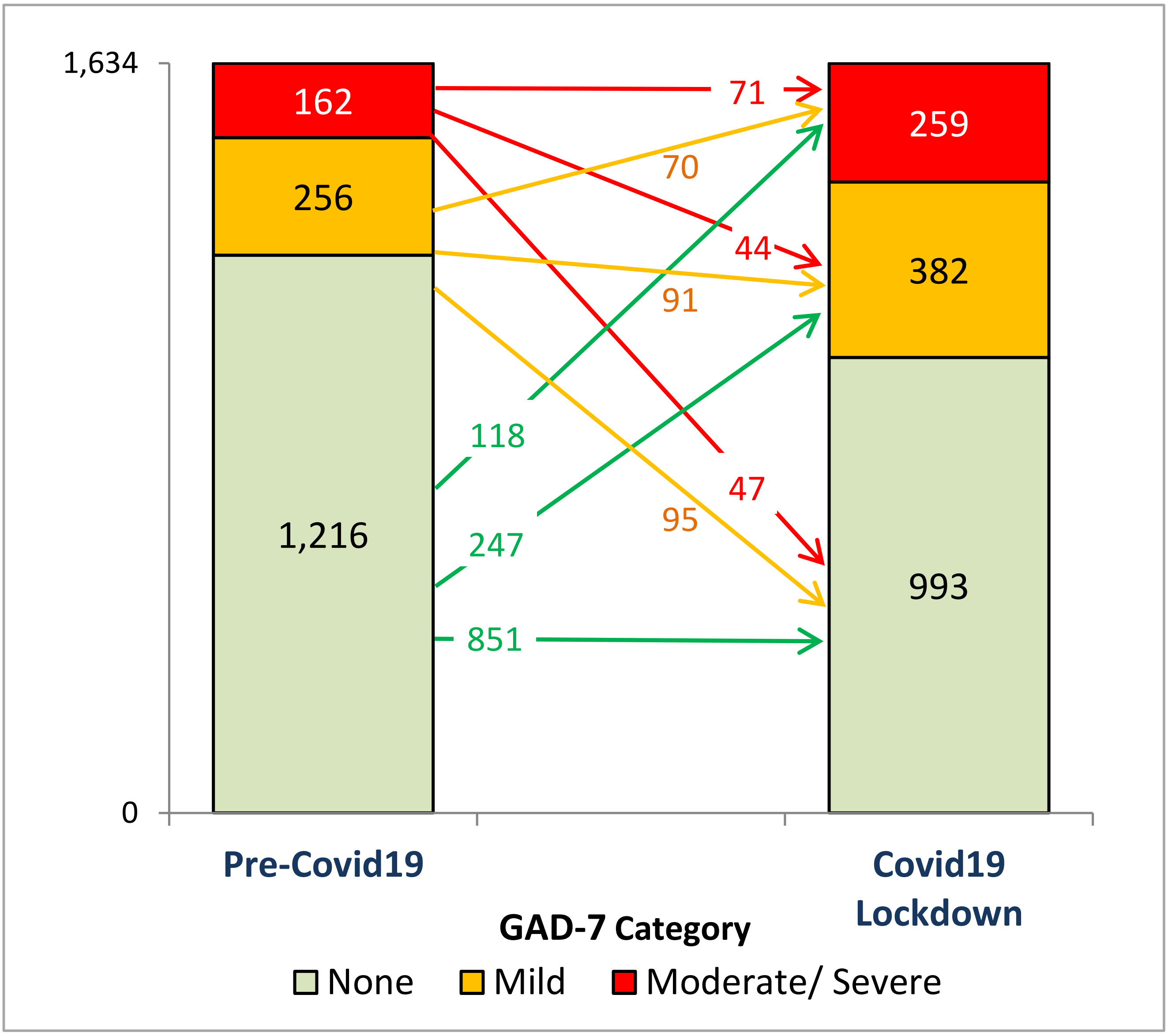
1,634
0
162
256
1,216
259
382
993
71
70
44
91
118
247
47
95
851
Pre-Covid19
Covid19 Lockdown
GAD-7 Category
None
Mild
Moderate/ Severe

Supplementary Tables S1 to S9: Multivariate models for increase in PHQ-8 and GAD-7 with ethnicity and covariates measuring food, housing and employment insecurity; loneliness; social support; quality of relationship with partner; self-isolating and vulnerable households; and physical activity.

All models for change in PHQ-8 also control for baseline PHQ-8, and all models for change in GAD-7 also control for baseline GAD-7. None of these covariates led to significant differences in ethnicity, with the exception of physical activity: after controlling for levels of physical activity Pakistani heritage respondents were significantly less likely to experience an increase in PHQ-8 of 5 points or more.

	PHQ-8		GAD-7	
**Table S1: Ethnicity and Food Security**	**Odds Ratio**	**(95% CI)**	**Odds Ratio**	**(95% CI)**
**Ethnicity (Reference: White British)**				
Pakistani heritage	0.84	(0.64-1.10)	0.89	(0.68-1.17)
Other	0.87	(0.61-1.25)	0.80	(0.55-1.16)
**Food security (Reference: Secure - Never or sometimes true that food does not last)**				
Insecure - Often true that food does not last	3.48	(2.18-5.54)	3.63	(2.24-5.87)

	PHQ-8		GAD-7	
**Table S2: Ethnicity and Housing Security**	**Odds Ratio**	**(95% CI)**	**Odds Ratio**	**(95% CI)**
**Ethnicity (Reference: White British)**				
Pakistani heritage	0.83	(0.63-1.09)	0.87	(0.66-1.14)
Other	0.75	(0.53-1.08)	0.73	(0.50-1.06)
**Housing security (Reference: Secure - Not worried about eviction/ repossession)**				
Insecure - Worried about eviction/ repossession	3.33	(2.37-4.68)	3.04	(2.13-4.35)

	PHQ-8		GAD-7	
**Table S3: Ethnicity and Job Security**	**Odds Ratio**	**(95% CI)**	**Odds Ratio**	**(95% CI)**
**Ethnicity (Reference: White British)**				
Pakistani heritage	0.88	(0.63-1.23)	0.84	(0.59-1.19)
Other	0.68	(0.43-1.10)	0.72	(0.44-1.18)
**Job security: whether expect main earner to still have job in 12 mth (Reference: Yes)**				
No	0.88	(0.52-1.51)	1.01	(0.59-1.73)

	PHQ-8		GAD-7	
**Table S4: Ethnicity and Relationship Quality**	**Odds Ratio**	**(95% CI)**	**Odds Ratio**	**(95% CI)**
**Ethnicity (Reference: White British)**				
Pakistani heritage	0.97	(0.72-1.31)	0.91	(0.67-1.23)
Other	1.10	(0.75-1.63)	0.87	(0.57-1.30)
**Quality of relationship with partner (Reference: Excellent)**				
Good	1.84	(1.38-2.46)	1.74	(1.29-2.35)
Average to very poor	3.65	(2.44-5.46)	5.08	(3.37-7.65)

For peer review only

Table S5: Ethnicity and Loneliness	PHQ-8 Odds Ratio	(95% CI)	GAD-7 Odds Ratio	(95% CI)
**Ethnicity (Reference: White British)**				
Pakistani heritage	0.86	(0.65-1.14)	0.89	(0.67-1.19)
Other	0.92	(0.64-1.33)	0.83	(0.56-1.21)
**Loneliness (Reference: not lonely most/ all of the time)**				
Lonely most/ all of the time	8.63	(5.86-12.73)	8.78	(5.87-13.12)

Table S6: Ethnicity and Social Support	PHQ-8 Odds Ratio	(95% CI)	GAD-7 Odds Ratio	(95% CI)
**Ethnicity (Reference: White British)**				
Pakistani heritage	0.85	(0.65-1.11)	0.90	(0.69-1.18)
Other	0.87	(0.61-1.23)	0.77	(0.53-1.11)
**Social support (Reference: Easy to get help from friends/ neighbours if needed)**				
Not Easy	2.33	(1.82-2.97)	2.21	(1.72-2.83)

Table S7: Ethnicity and Physical Activity	PHQ-8 Odds Ratio	(95% CI)	GAD-7 Odds Ratio	(95% CI)
**Ethnicity (Reference: White British)**				
Pakistani heritage	0.73	(0.56-0.96)	0.81	(0.61-1.07)
Other	0.82	(0.57-1.16)	0.75	(0.52-1.08)
**Frequency of physical activity (Reference: Every day)**				
Never	3.33	(2.25-4.92)	2.70	(1.79-4.07)
1 or 2 days a week	2.03	(1.46-2.83)	1.73	(1.23-2.43)
Most days a week	1.54	(1.11-2.15)	1.70	(1.22-2.36)

Table S8: Ethnicity and Self-isolating	PHQ-8 Odds Ratio	(95% CI)	GAD-7 Odds Ratio	(95% CI)
**Ethnicity (Reference: White British)**				
Pakistani heritage	0.87	(0.67-1.13)	0.92	(0.71-1.20)
Other	0.91	(0.65-1.29)	0.83	(0.57-1.19)
**Whether anyone in household is currently/ has previously self-isolated (Reference: No)**				
Yes	1.57	(1.22-2.03)	1.47	(1.13-1.92)

Table S9: Ethnicity and COVID19 Vulnerable Households	PHQ-8 Odds Ratio	(95% CI)	GAD-7 Odds Ratio	(95% CI)
**Ethnicity (Reference: White British)**				
Pakistani heritage	0.81	(0.62-1.06)	0.88	(0.68-1.16)
Other	0.92	(0.65-1.30)	0.82	(0.57-1.18)
**Whether anyone in household is clinically vulnerable to Covid-19 (Reference: No)**				
Yes	1.76	(1.35-2.30)	1.53	(1.16-2.02)

For peer review only

BMJ Open

“When will this end? Will it end?” The impact of the March-June 2020 UK Covid-19 lockdown response on mental health: a longitudinal survey of mothers in the Born in Bradford study.

Journal:	*BMJ Open*
Manuscript ID	bmjopen-2020-047748.R1
Article Type:	Original research
Date Submitted by the Author:	12-Jul-2021
Complete List of Authors:	Dickerson, Josie; Bradford Institute for Health Research<br>Kelly, Brian; Bradford Institute for Health Research<br>Lockyer, Bridget; Bradford Institute for Health Research<br>Bridges, Sally; Bradford Institute for Health Research<br>Cartwright, Christopher; Bradford Institute for Health Research<br>Willan, Kathryn ; Bradford Institute for Health Research<br>Shire, Katy; Bradford Institute for Health Research<br>Crossley, Kirsty; Bradford Institute for Health Research<br>Bryant, Maria; University of York, Health Sciences<br>Siddiqi, Najma; University of York, Health Sciences; Hull York Medical School<br>Sheldon, T; Bradford Institute for Health Research; Queen Mary University of London Barts and The London School of Medicine and Dentistry, Institute of Population Health Sciences<br>Lawlor, Deborah; University of Bristol, MRC Integrative Epidemiology Unit at The University of Bristol; University of Bristol Medical School, Population Health Science<br>Wright, John; Bradford Institute for Health Research<br>McEachan, Rosemary; Bradford Institute for Health Research, Bradford, United Kingdom; University of Bradford Faculty of Life Sciences<br>Pickett, Kate; University of York, Health Sciences
**Primary Subject Heading**:	Public health
Secondary Subject Heading:	Mental health, Public health
Keywords:	COVID-19, MENTAL HEALTH, Depression & mood disorders < PSYCHIATRY, Anxiety disorders < PSYCHIATRY, PUBLIC HEALTH

SCHOLARONE™
Manuscripts

I, the Submitting Author has the right to grant and does grant on behalf of all authors of the Work (as defined in the below author licence), an exclusive licence and/or a non-exclusive licence for contributions from authors who are: i) UK Crown employees; ii) where BMJ has agreed a CC-BY licence shall apply, and/or iii) in accordance with the terms applicable for US Federal Government officers or employees acting as part of their official duties; on a worldwide, perpetual, irrevocable, royalty-free basis to BMJ Publishing Group Ltd ("BMJ") its licensees and where the relevant Journal is co-owned by BMJ to the co-owners of the Journal, to publish the Work in this journal and any other BMJ products and to exploit all rights, as set out in our licence.

The Submitting Author accepts and understands that any supply made under these terms is made by BMJ to the Submitting Author unless you are acting as an employee on behalf of your employer or a postgraduate student of an affiliated institution which is paying any applicable article publishing charge ("APC") for Open Access articles. Where the Submitting Author wishes to make the Work available on an Open Access basis (and intends to pay the relevant APC), the terms of reuse of such Open Access shall be governed by a Creative Commons licence – details of these licences and which Creative Commons licence will apply to this Work are set out in our licence referred to above.

Other than as permitted in any relevant BMJ Author's Self Archiving Policies, I confirm this Work has not been accepted for publication elsewhere, is not being considered for publication elsewhere and does not duplicate material already published. I confirm all authors consent to publication of this Work and authorise the granting of this licence.

For peer review only

"When will this end? Will it end?" The impact of the March-June 2020 UK Covid-19 lockdown response on mental health: a longitudinal survey of mothers in the Born in Bradford study.

Josie Dickerson[1], Brian Kelly[1], Bridget Lockyer[1], Sally Bridges[1], Christopher Cartwright[1], Kathryn Willan[1], Katy Shire[1], Kirsty Crossley[1], Maria Bryant[1,2] Najma Siddiqi[3], Trevor A Sheldon[1,4], Deborah A Lawlor[5,6,7], John Wright[1], Rosemary RC McEachan[1,8], Kate E Pickett[1,2] on behalf of the Bradford Institute for Health Research Covid-19 Scientific Advisory Group (https://www.bradfordresearch.nhs.uk/c-sag/).

Corresponding author:

Josie Dickerson, Bradford Institute for Health Research, Bradford Teaching Hospitals NHS Foundation Trust, Bradford Royal Infirmary, Duckworth Lane, Bradford, BD9 6RJ, UK.

Email: josie.dickerson@bthft.nhs.uk

Telephone: 01274 383916

Addresses

[1] Bradford Institute for Health Research, Bradford Teaching Hospitals NHS Foundation Trust, Bradford Royal Infirmary, Duckworth Lane, Bradford, UK.

[2] Department of Health Sciences, University of York, Department of Health Sciences, Seebohm Rowntree Building, University of York, Heslington, York, UK.

[3] The Hull York Medical School, University of York, Heslington York, UK

[4] Institute of Population Health Sciences, Barts and The London School of Medicine and Dentistry, Queen Mary University London, Yvonne Carter Building, 58 Turner Street, London, UK

[5] MRC Integrative Epidemiology Unit at the University of Bristol, Oakfield House, Oakfield Grove, Bristol, , UK

[6] Population Health Science, Bristol Medical School, University of Bristol, UK

[7] Bristol National Institute for Health Research Biomedical Research Centre, UK

[8] Faculty of Life Sciences and Health Studies, University of Bradford, Richmond Rd, Bradford, UK

KEYWORDS

Covid-19; mental health; depression; anxiety; health inequalities; ethnic minorities

WORD COUNT: 5,506

For peer review only - http://bmjopen.bmj.com/site/about/guidelines.xhtml

ABSTRACT

Objectives To explore clinically important increases in depression/anxiety from before to during the first UK Covid-19 lockdown and factors related to this change, with a particular focus on ethnic differences.

Design Pre-Covid and lockdown surveys nested within two longitudinal Born in Bradford cohort studies.

Participants 1,860 mothers with a child aged 0-4 or 9-13, 47% Pakistani heritage

Main outcome measures Odds ratios (OR) for a clinically important increase (5 points or more) in depression (PHQ-8) and anxiety (GAD-7) in unadjusted regression analyses, repeated with exposures of interest separated by ethnicity to look for differences in magnitude of associations, and lived experience of mothers captured in open text questions.

Results The number of women reporting clinically important depression/anxiety increased from 11% to 19% and 10% to 16% respectively from before to during the first Covid-19 lockdown. Increases in depression/anxiety were associated with loneliness (OR: 8.37, 95% CIs: 5.70-12.27; 8.50, 5.71-12.65 respectively), financial insecurity (6.23, 3.96-9.80; 6.03, 3.82-9.51). Food and housing insecurity, a lack of physical activity and a poor partner relationship were also associated.

Pakistani heritage mothers who were lonely or had a poor partner relationship had greater odds of worsening mental ill health compared to White British mothers with these exposures. White British mothers who were financially insecure or physically inactive had greater odds of worsening mental ill health than Pakistani mothers. Responses to open text questions illustrated this complex inter-play of challenges contributing to mental ill health.

Conclusions Mental ill health has worsened for many during the Covid-19 lockdown, particularly in those who are lonely and economically insecure. The magnitude of associations between key exposures and worsening mental health varied between ethnic groups. Mental health problems may have longer term consequences for public health and interventions that address the potential causes are needed.

STRENGTHS AND LIMITATIONS OF THIS STUDY

- Two key longitudinal studies have highlighted that the Covid-19 pandemic and lockdowns have had a negative impact on mental health, particular in younger adults, women and those from low socio-economic circumstances, but with participants of predominantly White European ethnicity.
- Mental ill health is often reported to be more prevalent in people from ethnic minorities and the socially and economically disadvantaged. The Born in Bradford research programme offers a unique opportunity to investigate the impact of Covid-19 lockdown on mental health in a deprived and ethnically diverse population.
- This is a longitudinal study containing linked data collected before the Covid-19 pandemic and during the March-June 2020 lockdown which has allowed us to explore change over that time period in a highly ethnically diverse population, the majority of whom live in the most deprived centiles in the UK.
- Respondents in this study were mothers of children aged 0-5 and/or 9-13 which may limit the wider generalisability, though our findings are broadly similar (in prevalence and associations) to those from another longitudinal study that included adult men and women.
- We are not aware of other studies that have explored longitudinal change in mental health from pre- to during- the Covid-19 lock down in a similar ethnically diverse deprived population.

For peer review only - http://bmjopen.bmj.com/site/about/guidelines.xhtml

Introduction

There is growing concern that the ‘lockdown’ measures to control the spread of the Covid-19 pandemic[1,2] have had unintended consequences including an increase in mental ill health. Several studies since the Covid-19 pandemic began have reported high levels of depression and anxiety[3-7] in the UK. However, the majority of these surveys are either cross-sectional or longitudinal *within* the lockdown period[5-7] with only two studies[3,4] comparing mental health in the pre-Covid-19 period to mental health during the Covid-19 lockdown. One of those used Office of National Statistics data and found worsening mental health in younger adults, those who were financially insecure, and those who had a disability[3]. The second, using data from two prospective cohorts, found worsening mental health during, compared to before, the lockdown in: younger adults, women, those with pre-existing mental and physical health conditions, those living alone and in socio-economic adversity[4]. In these two studies, most participants were of white European origin and the larger of the two studies was in a relatively affluent population. Mental ill health is often reported as being more prevalent in people from ethnic minorities and the socially and economically disadvantaged[8,9], but no longitudinal research to date has investigated the impact of Covid-19 lockdown on mental health in these populations[10].

We were able to explore these questions in depth by building on the Born in Bradford research programme which includes two longitudinal birth cohorts of ethnically diverse families living in the city of Bradford, many in deprived circumstances. These cohorts have recent in-depth information on the demographics, socioeconomic status and mental health of mothers before the Covid-19 pandemic,[11-13] as well as mental health during the March-June 2020 Covid-19 lockdown[14], and so offer a unique opportunity to assess the impact of Covid-19 and lockdown longitudinally in a deprived and ethnically diverse population.

We used the data to:

- Describe the changes in the prevalence of depression and anxiety in mothers living in Bradford from before the Covid-19 pandemic to during the March-June 2020 Covid-19 lockdown.
    - Identify variables associated with a clinically important increase in mental ill health in this population, to identify vulnerable groups that may need additional support in the recovery from the pandemic.
    - Explore whether there is a difference in the magnitude of the associations of key exposures with an increase in clinically important mental ill health by ethnicity (Pakistani heritage compared to White British ethnicity).
    - To explore mothers’ lived experience during the March-June 2020 Covid-19 lockdown by assessing the frequency of worries and concerns relating to mental ill health obtained through free text responses to open questions.

Methods

Study Design

A longitudinal study using data collected at two time points, before and during the March-June 2020 Covid-19 lockdown, from mothers who participated in one of two prospective birth cohort studies in Bradford: Born in Bradford’s Growing Up (BiBGU) study, with parents of children currently aged 9-13[11,12] and Born in Bradford’s Better Start (BiBBS), with parents of children currently aged 0-4.[13]

Patient and Public Involvement

Born in Bradford is a ‘people powered’ research study; the local community were consulted to identify key research priorities during the March-June 2020 Covid-19 lockdown. This included consultation with key community groups, seldom-heard communities and local policy and decision makers to ensure that the focus of the research was relevant to local needs. The Covid-19 survey and recruitment approach were tested through our established research advisory groups. The findings of the study were also shared with these groups to enhance interpretation and ensure useful dissemination back to the community[14].

Consent

For peer review only - http://bmjopen.bmj.com/site/about/guidelines.xhtml

Participants had previously consented for their research data, and routinely collected health and education data, to be used for research. For the Covid-19 survey, verbal consent was taken for questionnaires completed over the phone and implied consent was assumed for all questionnaires completed via post or online.

Data Collection

Full details of the data collection of the March-June 2020 Covid-19 survey can be found elsewhere[14,15]. In summary:

Participants were recruited using a combination of emails, text and phone with a follow-up postal survey, and in their main language wherever possible.

a) Pre-Covid-19 data

Pre-Covid-19 data for BiBGU participants were derived from two sources: a) participant ethnicity and age were collected during pregnancy (2007-2011); [11] b) recent follow-up data on mental health (collected between 24th June 2017 and 12th March 2020).[12] Pre-Covid data for BiBBS participants were taken from data collected during pregnancy (6th January 2016 and 8th February 2020).[13] The median time since most recent pre-Covid data collection was 15 months (range 1 to 35) for BiBGU and 29 months (range 2 to 52) for BiBBS.

b) March-June 2020 Covid-19 lockdown data collection:

Mental health was measured using the PHQ-8[16] for depression and the GAD-7 for anxiety.[17] These are widely used measures of the severity of symptoms of depression and anxiety that have been validated in the general population, [16,17] and in many ethnic minorities including UK residents of Pakistani heritage.[18] Information was also collected on: household circumstances; family relationships; social support and loneliness; financial, employment, housing and food insecurity; physical health. This contextual information was captured in self-reported questions administered in the Covid-19 lockdown survey; details of variables used are in Table 1 and our protocol paper[14]. Information on the participants' lived experience during lockdown were captured using free text questions that asked "what are your three biggest worries at the moment?"; and "has anything been easier or more enjoyable in lockdown?"

Data Preparation

To describe the changes in prevalence of depression and anxiety from before the Covid-19 pandemic to during the Covid-19 lockdown we derived a categorical variable for each PHQ-8 and GAD-7 score based on the standard clinical scoring classification: 0 to 4 no depression, 5 to 9 mild depression, 10-24 moderate-severe depression; 0 to 4 no anxiety, 5 to 9 mild anxiety, 10 to 21 moderate-severe anxiety.[16,17] and then collapsed moderate, moderately severe and severe categories to indicate clinically important symptoms of depression and anxiety. To identify variables associated with an increase of mental ill health we used an increase in PHQ-8 and GAD-7 scores of 5 or more points as an indicator of clinically important change in symptoms. This cut-off was chosen following guidance from previous research and consultation with clinical colleagues[19]. An increase of five points or more would also always result in a change in the categorisation of symptoms (e.g. from none to mild or mild to moderate/severe) whilst also capturing changes in severity within the moderate/severe categories.

Ethnicity was coded using Census 2011 categories and also as 'White British', 'Pakistani Heritage' and 'Other', which included all other groups due to small numbers of a wide range of ethnicities. Given the heterogeneity of the 'Other' ethnic group, the decision was made to not use this group in these analyses, but to focus on comparing those of Pakistani heritage and White British ethnicity.

A number of categories within other explanatory variables were collapsed for analysis. These included: Quality of relationship with partner: Average to poor (comprising of 'average', 'poor' and 'very poor'); Loneliness: Not lonely (comprising of: 'none or almost none of the time' and 'some of the time') and Lonely (comprising of: 'Most of the time' and 'All or almost all of the time'); Social support: Easy to get support (comprising of: 'Very easy' and 'easy'); and Not easy to get support (comprising of: 'Very difficult', 'Difficult'

For peer review only - http://bmjopen.bmj.com/site/about/guidelines.xhtml

and 'Possible'); Food insecurity: Secure (Comprising of' never true' or 'sometimes true' that food didn't last) and Insecure (Comprising of 'Often true' that food didn't last). Housing security: Secure (Comprising: 'Strongly Disagree', 'Disagree' and 'Neither disagree or agree' that I worry about being evicted or having my home repossessed) and Insecure (comprising of 'Strongly Agree' or 'Agree').

Missing data on measures was small for most variables (Table 1) and was not adjusted for in the analyses.

Data analysis

To describe the changes in the prevalence of depression and anxiety from before to during the pandemic we explored the changes in PHQ-8 and GAD-7 categories using descriptive statistics and presented these visually using flow diagrams to elucidate the patterns of both positive and negative changes.

We used descriptive statistics to present results of sample characteristics, including depression and anxiety at pre-Covid-19 and Covid-19 lockdown survey time points.

We then modelled the odds ratios associated with an increase in PHQ-8 and GAD-7 scores by five or more points using separate unadjusted logistic regression models for each exposure variable of interest. Exposure variables included in the analyses were identified as indicative of an increase in mental ill-health in the lockdown survey findings reported previously.[15] Pre-Covid-19 PHQ-8 and GAD-7 scores were controlled for in each model.

In order to explore whether or not the magnitude of the association between exposure variables and a clinically important increase in symptoms of depression and anxiety differed between ethnic groups, we repeated the above analyses for Pakistani heritage and White British participants separately. This approach avoids the difficulties inherent in interpreting the ethnicity coefficient in regression models controlling for other variables[20]. All statistical analyses were carried out using Stata 15.[21]

To explore mothers' lived experience during the March-June 2020 Covid-19 lockdown, the free text responses to open questions were reviewed and themes that related to mental health of the mothers were pulled out. Responses were coded using thematic analysis.[22] The first 100 responses were analysed by one researcher (BL), employing an inductive approach where coding and theme development were driven by the content of the responses. Two codebooks were developed, one for the questions on the three biggest worries and recent challenges during lockdown and another smaller codebook for the question on what had been made more enjoyable and easier during lockdown. Using Microsoft Excel, the remaining responses were then coded by three different researchers in order to test the strength and validity of the codebooks. Through frequent discussion between the researchers about this process, adjustments were made to the original codebooks so that they were reflective of the total responses. The emergent themes relating to mental health were used to illuminate the findings from the quantitative analyses.

Ethics

This study was approved by the HRA and Bradford/Leeds research ethics committee (Substantial amendments to: BiBGU 16/YH/0320 and BiBBS 15/YH/0455)

Results

Study Population

2,144 (28%) of those invited participated in the Covid-19 survey between 10th April and 30th June 2020. Full details of the study population are described elsewhere[15], in summary: Participants were broadly representative of the invited cohort: 47% were of Pakistani heritage, 35% White British and 18% other ethnic groups; 46% lived in the most deprived decile of material deprivation in England. Baseline (i.e. prior to the Covid-19 pandemic) levels of depression and anxiety in participants and non-participants were also similar (see Supplementary Table 1).

Of the 2,144 mothers who responded to the survey, 1,860 (87%) had complete surveys and linked data from pre-Covid19 surveys. Of these; 1,316 (71%) were in the BiBGU cohort (with a child aged 9-13) and

For peer review only - http://bmjopen.bmj.com/site/about/guidelines.xhtml

544 (29%) were in the BiBBS cohort (with a child aged 0-4). Respondents were representative of the BiB and BiBBS cohorts (see Supplementary Table 1).[15]
The mothers had a mean age of 37.5 years (SD 6.8). 877 (48%) were of Pakistani heritage, 613 (34%) of White British ethnicity and 320 (18%) of other ethnicities. Table 1 describes the sample characteristics of the study population.

[Table 1 here]

Study Sample Change in Prevalence of Depression and Anxiety

The prevalence of moderate / severe depression increased between the pre-Covid-19 and Covid-19 lockdown surveys from 11% (n=212) to 19% (n=349). The proportion of mothers reporting mild symptoms of depression remained similar; while the proportion of those with no depressive symptoms decreased from 65% (n=1187) to 56% (n=1001), (Table 2). The proportion of mothers with moderate/severe anxiety increased from 10% (n=167) to 16% (n=289). The prevalence of mild anxiety also increased from 16% (n=270) to 23% (n=408), whilst the proportion of participants with no anxiety fell from 75% (n=1280) to 61% (n=1075) (see Table 2).

[Table 2 here]

Within-Mothers Change in Depression and Anxiety
1,760 participants had both pre-Covid19 and Covid-19 lockdown depression data and 1634 had pre-Covid19 and Covid-19 lockdown anxiety data. For these mothers, Figures 1 and 2 show the flow of individuals from different categories of depression and anxiety across the two time points (pre-pandemic and during lockdown).
The majority of mothers stayed in the same depression and anxiety categories across the two time points, for example 67% (n=759) who had no symptoms in the pre-Covid-19 data continued to have no symptoms during lockdown; and 54% (n=109) of those who had moderate/severe symptoms before the pandemic, remained in this category in the lockdown survey). However, many mothers' mental health worsened, with 38% (n=230) who had no/mild symptoms pre-Covid-19 reporting moderate/severe symptoms in the lockdown survey. A smaller number of mothers' mental health improved: of those mothers with moderate/severe symptoms pre-Covid-19, 24% (n=48) subsequently reported no symptoms and 23% (n=46) reported mild depression in the Covid-19 survey. Similar patterns of change were seen with the anxiety categories (see also Supplementary Table 2).

[Figure 1 & 2 here]

367 (21%) of mothers reported a clinically important increase (5 or more points) in depressive symptoms, and 348 (21%) reported a clinically important increase (5 or more points) in anxiety symptoms. Table 3 presents the odds ratios for a clinically important increase in PHQ-8 and GAD-7 scores in relation to each exposure variable from the unadjusted logistic regression models. The estimates resulting from these models are imprecise, with wide confidence intervals.

Compared to White British mothers, the odds of an increase in clinically important depression was reduced by 12% in Pakistani women (Odds Ratio (OR) 0.88 [95% Confidence Intervals: 0.68-1.15]), but there was no clear association for anxiety (OR 0.94: [0.72-1.23]). Financial, food and housing insecurity during lockdown were associated with a higher likelihood of a clinically important increase in both depression and anxiety symptoms: the odds were more than 6 times greater for women who were financially insecure (OR 6.23 [3.96-9.8] for depression; OR 6.03 [3.82-9.51] for anxiety) and over 3 times greater in mothers who were food insecure (OR 3.33 [2.09-5.28] for depression; OR 3.46 [2.15-5.58] for anxiety) or housing insecure (OR 3.29 [2.36-4.58] for depression; OR 3.0 [2.11-4.25] for anxiety).

Social circumstances were also associated with an increase in depression and anxiety: The odds of increased depression or anxiety were more than eight times greater in mothers reporting loneliness (OR 8.37 [5.7-12.27] for depression; OR 8.5 [5.71-12.65] for anxiety), and a lack of social support doubled the likelihood of an increase in depression and anxiety (OR 2.25 [1.78-2.86]; OR 2.13 [1.67-2.73] respectively).

For peer review only - http://bmjopen.bmj.com/site/about/guidelines.xhtml

An average / poor relationship increased the odds of experiencing symptoms of depression by 3.6 [2.44-5.43] and of anxiety by 5.1 [3.37-7.62]. A larger household size reduced the OR for depressive symptoms (OR 0.73 [0.53-0.99]), but less so for anxiety (OR 0.90 [0.63-1.29].

A lack of physical activity during lockdown was associated with an increased OR for both depression and anxiety (OR 3.13 [2.15-4.56]; OR 2.55 [1.72-3.78], respectively). Mothers who did the same amount of physical activity during lockdown as they had done pre-Covid-19 reduced the odds of an increase in depressive symptoms by 34% (OR 0.66 [0.45-0.97]).

Compared to mothers in the BiBGU cohort, the odds of an increase in depression or anxiety was reduced by 16% for mothers in the BiBBS cohort. Cohort differences included a) pregnancy status at pre-pandemic data collection (BiBBS=pregnant; BiBGU= primary school aged child); and b) child age at Covid-19 data collection (BiBBS=pre-school; BiBGU=primary/secondary).

[Table 3 here]

When the unadjusted regression analysis was repeated separately for White British and Pakistani heritage mothers, interesting differences in the magnitude of the association between exposure variables and a clinically important increase in symptoms of depression and anxiety was found (Table 4a and 4b). The odds of an increase in depression and anxiety were greater for Pakistani heritage women who reported loneliness (OR 11.22 [6.45-19.53] for depression and OR 11.27 [6.16-20.56] for anxiety) compared to White British mothers (OR 7.17 [3.66-14.01] and OR 5.90 [3.03-11.47] respectively). There was also a greater magnitude of association for increased depression, but not anxiety, for Pakistani heritage women who reported an average / poor relationship with their partner (OR 4.91 [2.78-8.67] for depression and OR 4.99 [2.72-9.14] for anxiety), compared to White British mothers (OR 2.61 [61.31-5.20] and OR 4.39 [2.26-8.53] respectively).

In contrast the magnitude of the odds of an increase in depression and anxiety was greater for White British women who reported financial insecurity (OR 12.14 [5.15-28.60] for depression and OR 7.69 [3.43-17.26] for anxiety) or never doing physical activity (OR 5.54 [2.69-11.43] and OR 3.90 [1.82-8.36] respectively) compared to Pakistani heritage women (financial insecurity: OR 5.79 [2.90-11.56] for depression and OR 4.35 [2.27-8.30] for anxiety); (physical activity: OR 3.34 [1.88-5.92] and OR 2.50 [1.40-4.45] respectively).

For Pakistani heritage mothers living in large households, the odds of an increase in depression (OR 0.54 [0.31-0.94]) and anxiety (OR 0.61 [0.34-1.07]) was much reduced compared to White British mothers (OR 0.91 [0.47-1.73] for depression and OR 1.13 [0.58-2.20] for anxiety).

[Table 4 here]

Free text responses to the question “what are your three biggest worries at the moment” were available for 1799 mothers. Only a small proportion of women identified their mental health issues as one of their biggest worries, n=105 (6%, 95%CI: 5%-7%), slightly greater in White British mothers, n=51 (8%, 95%CI: 6%-11%) than in mothers of Pakistani heritage, n=32 (4%, 95%CI: 3%-5%). More often, mothers reported how wider issues and concerns impacted on their mental health and wellbeing:

Health anxieties about Covid-19: the most commonly reported worry was a fear of bringing the virus home (e.g. from the shops or from their places of work), and themselves or members of their family becoming ill or dying, as well as the fear of what would happen to their children if this did happen to them.

I worry about contracting coronavirus particularly whilst at work and either becoming critically myself unwell or bringing it home to my family

Feeling anxious about the virus and constantly worrying about my kids which 2 of them have health issues and are quite vulnerable

I feel particularly anxious to even step out of the house even for food shopping or taking a walk/exercise

I worry how this will affect my children. I'm terrified they will be separated as I have 2 children with my ex-husband and one with my current. So I haven't been outside in 10 weeks.

For peer review only - http://bmjopen.bmj.com/site/about/guidelines.xhtml

Mental load: mothers often reported the mental load of managing work, home-schooling, childcare and domestic tasks, without the break provided by children attending school, nursery or other childcare. Being or feeling stuck inside and unable to move around freely contributed to a sense of suffocation and feeling overwhelmed, and many mothers acknowledged that this was having a detrimental effect on their mental health and self-esteem:

I'm worried [about] having a nervous breakdown or a panic attack...can't get a break from all the responsibilities and go somewhere for fresh air even

Finding working from home and looking after children very demanding. No time alone. No silence. Surrounded by people and electronics all my waking hours.

I am a keyworker who works five days of week, only while my children are at school. I am becoming mentally drained/exhausted constantly being on duty, it is impacting my mental health.

Homeschooling I am not cut out for this and don't feel good enough

Loss of social support: The loss of social support caused by lockdown, especially for those who didn't live with their partner or were single parents was highlighted as causing loneliness and isolation for some.

My support network was my partner, who I cannot see due to lockdown.

Not able to see my boyfriend - feel isolated and alone once children are in bed with no adult face to face interaction

The isolation and loneliness has been a challenge and having to take care of my child on my own full-time

Being a single parent of a disabled child I rely on my social life/friends for my mental wellbeing and I worry about when it'll be safe to see/hug my immediate family who live locally

Financial and employment insecurity: Household finances and the stress of unemployment or job insecurity related to lockdown measures were also a major worry for women. Many families were in debt, reliant on credit cards and benefits, and in insecure employment before the lockdown measures began and were only just about getting by prior to the pandemic.

I have struggled financially during this time. I would like to not worry about money and bills and shopping and outgoings. I have enough worries being a full time carer.

Worried about the financial impact of covid 19. I am currently furloughed from work but I worry that the virus will have an impact on the business. My husband is self-employed and is not eligible to any funds.

Being unable to switch off: Participants described being frightened of the news reports but unable to switch off, and were wondering when, if ever, things would become normal again:

Worry about my mental health, I know I'm struggling. Worry about the future and how this will affect the country financially. Worry about my children's education & what they've missed out on.

All the bad news on the TV, and the death rate on the News. All the information on the news makes me panic more.

When will this end? Will it end?

A loss of coping strategies: For those who had existing mental health issues before lockdown, the lockdown measures had often taken away their sources of support, their normal routines and methods of coping. In addition, some respondents reported being unable to access mental health services due to Covid-19 and lockdown measures:

Mental health - Exercise at the gym was a coping mechanism, now closed. Can do bits at home but nothing like at the gym. Cannot get any gym equipment - out of stock.

Mental Health - struggling to motivate myself and to keep to a routine. Self-destructive behaviour - drink drugs and binge eating. I am missing my work and colleagues.

For peer review only - http://bmjopen.bmj.com/site/about/guidelines.xhtml

Not getting my mental health support since the lockdown. My CPN (Community Psychiatric Nurse) not returning my calls. It has made me a lot worse. I try to talk to my husband so I am not keeping everything inside

Mental health, I have had previous issues in the past and am struggling and don't feel like I can approach my GP at the minute as it isn't an emergency

Positive aspects of lockdown: Many participants reported positive aspects to changes enforced by the lockdown, commenting that they were getting to spend more quality time with their children, were enjoying a slower pace of life, a more relaxed routine and spending less time driving and commuting.

Life has become a lot more relaxed over the last 3 weeks, no manic mornings trying to get everybody out of the house, time with kids, doing stuff with kids I would normally say 'not now' to. Get to know kids more. More time outside, [doing] jobs in the house that need doing.

Spending more time with my husband and feeling appreciative of each other and having a relaxed day with the children instead of running to school to mosque and then all the extra clubs it's a more relaxed day.

Ramadan is the easiest it has ever been, we are free to make up our sleep and not push ourselves too much, had time to do nice things during Ramadan including having a more peaceful time not having to do school runs, be stressed out, my husband had a chance to take a slower pace to life and not get too stressed.

DISCUSSION

We compared depression and anxiety during the March-June 2020 UK lockdown to pre-Covid19 depression and anxiety data collected in our longitudinal birth cohort studies. We found that clinically important symptoms of depression and anxiety increased from 11% to 19% and from 11% to 16%, respectively. These findings reflect those of other longitudinal studies which have reported similar changes in mental health from before to during the Covid-19 pandemic[3,4].

We hypothesised that our vulnerable population with diverse ethnicity and high levels of deprivation would be susceptible to increases in depression and anxiety during the lockdown. Financial, food and housing insecurity all increased the odds of an increase in depression and anxiety, as did loneliness, a lack of social support, an average/poor partner relationship and a lack of physical activity.

We also observed reduced odds of depression and anxiety in Pakistani compared to White British mothers. A recent study has identified larger increases in mental ill health from before to during the pandemic in school age children of White British compared to minority ethnic groups in the UK, which is consistent with our findings[23]. When we separated out the regression analyses by ethnicity, we found interesting differences in the magnitude of the associations with an increase in depression and anxiety. Mothers from a Pakistani heritage had greater odds of an increase in depression and anxiety if they were lonely or had an average/poor relationship (for depression, but not anxiety) than White British mothers. Pakistani heritage mothers had a much reduced odds of an increase in depression or anxiety if they lived in a large household compared to White British mothers.

In contrast, mother of White British ethnicity had greater odds of an increase if they were financially insecure and/or physically inactive compared to Pakistani heritage mothers reporting the same exposures. The free text responses supported these findings, highlighting a complex inter-play of challenges contributing to poor mental health in mothers including: acute health anxieties; the mental load of managing multiple roles and responsibilities; the loss of social support and other coping strategies; pressures of financial and employment insecurity; and being unable to switch off from the pandemic.

The ethnic differences reported in this study warrant further investigation, including an understanding of whether there are ethnic differences in relation to disclosing mental ill health symptoms, and potentially differing protective factors in different ethnic groups. We have previously reported, outside of the Covid-19 context, that (a) White British mothers are more likely to have their mental ill health identified by health professionals than South Asian women, and that (b) both White British and South Asian are equally likely to disclose symptoms in self-report research questionnaires, as used here.[24,25] Ethnic differences in household structure, and cultural practices might provide more support in times of adversity, as we have found in the case of food insecurity previously [26,27].

For peer review only - http://bmjopen.bmj.com/site/about/guidelines.xhtml

This is a longitudinal study comparing data collected before the Covid-19 pandemic and during the March-June 2020 lockdown which has allowed us to explore change over that time period. It also provides findings from a highly ethnically diverse population, the majority of whom live in the most deprived centiles in the UK. We are not aware of other studies that have explored longitudinal change in mental health from pre- to during- the Covid-19 lockdown in a similar population.

Respondents were mothers of children aged 0-5 and/or 9-13 which may limit the wider generalizability, though our findings are broadly similar to those from a previous longitudinal study of two UK cohorts that included adult men and women (not all of whom were mothers), and found the increased risk of poor mental health in lockdown to be greater in women[4]. Our pre-COVID19 measures were taken from data collected over the past 4 years, so we cannot, with confidence, attribute all changes to the pandemic and the lockdown. For example, it is possible that some of the difference reflects age related change in the women and/or their children over time. It is also possible that we have underestimated some of the adverse impact of lockdown as a significant percentage of the BiBBS participants were pregnant at baseline, which itself is associated with raised levels of mental ill health. Indeed our analysis did find reduced odds of an increase in depression and anxiety in the BiBBS compared to the BiBGU cohort. However, our analyses did not find any strong association between time since baseline data capture and the odds of an increase in depression and anxiety.

We undertook unadjusted logistic regression analyses to explore possible factors that might explain or influence changes in mental health from before- to during- the pandemic, and repeated these separating out the two main ethnic groups. This approach avoids the difficulties inherent in interpreting the ethnicity coefficient in regression models controlling for other variables[20], however it also limits the interpretation of the data. In addition, the total sample size for this mental health analysis was ~1,700 mothers and many estimates in the analyses were, as a consequence, imprecise with wide confidence intervals. These results should be treated with some caution unless replicated in larger studies.

Whilst it is possible that our results are influenced by the survey response rate of 28%, although, these participants were representative of the BiB and BiBBS cohorts[15], including on the rates of baseline depression and anxiety scores, and have demonstrated a wide variability in most characteristics (Table 1).

We undertook stratified analyses to explore ethnic differences in how exposures related to changes in mental health between pre-pandemic times to during lockdown. However, whilst our analysis was able to look for differences between Pakistani heritage and White British mothers, we were unable to explore the heterogeneity of the 'other' ethnic group and we have no doubt missed important nuances in this population based on different social and cultural experiences.

We will continue to follow the BiBGU and BiBBS families so that we can look at trajectories of change over time, including during any future national or regional lockdowns. At time of writing (November 2020), the UK was under a further nationwide lockdown, following a period of reduced restrictions during summer 2020 and tighter regional restrictions in the more deprived cities of Northern England, including Bradford, though the autumn. The impact of further and longer periods of restrictions and lockdowns will be a focus of our study moving forwards. As the longitudinal follow-up lengthens and more data become available it may be possible to model different transitions in mental health in relation to changing social and economic circumstances. It may also be of interest to explore positive / null transitions from clinically important symptoms to mild/no symptoms, or continued mild/no symptoms despite reported exposure to adversity.

Our results highlight the potential public health impact of lockdown on mental health, particularly in those who are lonely and economically insecure and of White British ethnicity. Mental health problems are in general less visible than physical symptoms and in particular the physical symptoms related to Covid-19, including the acutely ill patients in ICUs, but may have more significant longer-term consequences. This study also suggests a complex interplay of factors between an individuals' circumstances and the odds of worsening mental health during the pandemic. A 'one-size fits all' approach to supporting mental ill health will not be effective, instead, understanding and addressing the potential causes in different groups will be important. For example, in case of future need for social distancing measures, government and local councils should consider policies that permit 'social bubbles' that can be implemented to reduce loneliness for those at risk of mental health problems, and they as well as voluntary services should continue to focus support to those who are lonely/isolated. Policy and decision makers should also make provision for the continuing need to support and protect vulnerable families from financial, food and housing insecurity, all of

For peer review only - http://bmjopen.bmj.com/site/about/guidelines.xhtml

which were associated with poor mental health in this study. These actions will be important throughout any further regional and national lockdowns and during the post-pandemic period of recovery.

For peer review only

For peer review only - http://bmjopen.bmj.com/site/about/guidelines.xhtml

DISSEMINATION DECLARATION

The findings of the study were also shared with our community research advisory groups to enhance interpretation. We will share our findings with all participants using a combination of social media, infographics posted to participants and community dissemination events

DATA (AND SOFTWARE) AVAILABILITY

Born in Bradford offer open access to their data resources. Available data and procedures to access this can be found at: https://borninbradford.nhs.uk/research/how-to-access-data/_

AUTHOR CONTRIBUTIONS

JD was involved in the concept and study design, design of the data collection tools, overall supervision of the study and data collection, wrote the statistical analysis plan, and drafted and revised the paper. She is guarantor. BK was involved in the design of data collection tools, wrote the statistical analysis plan, conducted all statistical analyses, and drafted and revised the paper. BL devised the coding framework for free text responses, analysed the data and drafted and revised the paper. SB was involved in the concept and study design, design of the data collection tools, supervision of the study and data collection, and reviewing and revising the paper. CC was involved in the concept and study design, and reviewing and revising the paper. KW was involved in design of the data collection tools, data curation, analysis, reviewing and revising the paper. KS was involved in the study design, design of the data collection tools, supervision of the study and data collection, and reviewing and revising the paper. KC was involved in the study design, design of the data collection tools, supervision of the study and data collection, data curation and reviewing and revising the paper. MB involved in the study design, and drafted and revised the paper. NS advised on the clinical significance of changes in mental ill health, revising the statistical analysis plan, and drafted and revised the paper. TS was involved in the concept and study design, supervision and support to junior members of the team and drafted and revised the paper. DAL was involved in the concept and study design, design of the data collection tools, and drafted and revised the paper. JW was involved in the concept and study design, design of the data collection tools, supervision and support to junior members of the team and drafted and revised the paper. RM was involved in the concept and study design, design of the data collection tools, revised the statistical analysis plan, and drafted and revised the paper. KP was involved in the concept and study design, design of the data collection tools, revised the statistical analysis plan, supervision and support to junior members of the team and drafted and revised the paper.

COMPETING INTERESTS

All authors have completed the ICMJE uniform disclosure form at www.icmje.org/coi_disclosure.pdf and declare: all authors had financial support from funding bodies listed below for the submitted work; no financial relationships with any organisations that might have an interest in the submitted work in the previous three years; no other relationships or activities that could appear to have influenced the submitted work, with the exception of DAL who declares previous support from Roche Diagnostics and Medtronic Ltd for research unrelated to that presented here.

FUNDING INFORMATION

This study was funded by The Health foundation Covid-19 Award (2301201), with further contributions from: A Wellcome Trust infrastructure grant (WT101597MA); A joint grant from the UK Medical Research Council (MRC) and UK Economic and Social Science Research Council (ESRC): MR/N024391/1; The National Institute for Health Research under its Applied Research Collaboration Yorkshire and Humber (NIHR200166); ActEarly UK Prevention Research Partnership Consortium (MR/S037527/1); Better Start Bradford through The National Lottery Community Fund; and the British Heart Foundation (CS/16/4/32482). DAL works in a unit that received support from the University of Bristol and UK Medical Research Council (MC_UU_00011/6) and is a UK National Institute of Health Research senior Investigator (NF-0616-10102)

ACKNOWLEDGEMENTS

Born in Bradford is only possible because of the enthusiasm and commitment of the children and parents in BiB. We are grateful to all the participants, parent governors and community research advisory group members, schools, health professionals and researchers who have made Born in Bradford happen.

This report is independent research partly funded by the National Institute for Health Research Yorkshire and Humber ARC. The views expressed in this publication are those of the authors and not necessarily those of the National Institute for Health Research or the Department of Health and Social Care.

TRANSPARENCY STATEMENT

The lead author (the manuscript's guarantor) affirms that the manuscript is an honest, accurate, and transparent account of the study being reported; that no important aspects of the study have been omitted; and that any discrepancies from the study as originally planned (and, if relevant, registered) have been explained.

For peer review only

For peer review only - http://bmjopen.bmj.com/site/about/guidelines.xhtml

1. REFERENCES

1. Coronavirus (Covid-19) UK Government Response. https://www.gov.uk/coronavirus. Accessed 27.11.20

2. Number of coronavirus (COVID-19) cases and risk in the UK. UK Government Guidance. https://www.gov.uk/guidance/coronavirus-covid-19-information-for-the-public . Accessed 27.11.20

3. Office of National Statistics (2020). Coronavirus and depression in adults, Great Britain: June 2020. https://www.ons.gov.uk/peoplepopulationandcommunity/wellbeing/articles/coronavirusanddepressioninadultsgreatbritain/june2020. Accessed 27.11.20

4. Kwong ASF, Pearson RM, Adams MJ, et al., Mental health during the COVID-19 pandemic in two longitudinal UK population cohorts. *MedRxiv* 2020; 20133116. doi: 10.1101/2020.06.16.20133116

5. Pierce M, Hope H, Ford T, et al., Mental health before and during the COVID-19 pandemic: a longitudinal probability sample survey of the UK population. *Lancet Psychiatry* 2020, https://doi.org/10.1016/S2215-0366(20)30308-4

6. Mind (2020). The mental health emergency: how has the coronavirus pandemic impacted our mental health? London: Mind. Available at: mind.org.uk. Accessed 27.11.20

7. Fancourt D, Steptoe A, Bu F. Trajectories of depression and anxiety during enforced isolation due to COVID-19: longitudinal analyses of 59,318 adults in the UK with and without diagnosed mental illness. medRxiv 2020. **doi:** https://doi.org/10.1101/2020.06.03.20120923

8. Bignall T, Jeraj S, Helsby E, Butt J. Racial disparities in mental health: Literature and evidence review. Race Equality Foundation 2019. https://raceequalityfoundation.org.uk/wp-content/uploads/2020/03/mental-health-report-v5-2.pdf. Accessed 27.11.20

9. Grey T, Sewell H, Shapiro G, Ashraf F. Mental Health Inequalities Facing U.K. Minority Ethnic Populations Causal Factors and Solutions. J Psychological Issues in Organizational Culture. 2013: 3 (S1).

10. Public Health England. COVID-19: mental health and wellbeing surveillance report: https://www.gov.uk/government/publications/covid-19-mental-health-and-wellbeing-surveillance-report/2-important-findings-so-far#which-population-groups-appear-to-be-disproportionally-affected. Accessed 27.11.20

11. Wright J, Raynor P, Tuffnell D, et al. Cohort Profile: The Born in Bradford multi-ethnic family cohort study. *International Journal of Epidemiology*, Volume 42, Issue 4, August 2013, Pages 978–991, https://doi.org/10.1093/ije/dys112

12. Bird PK, McEachan RRC, Mon-Williams M, Small N, West J, Whincup P, Wright J, Andrews E, Barber SE, Hill LJB *et al*: Growing up in Bradford: protocol for the age 7–11 follow up of the Born in Bradford birth cohort. *BMC Public Health* 2019, 19(1):939.

13. Dickerson J, Bird PK, McEachan RRC, Pickett KE, Waiblinger D, Uphoff E, Mason D, Bryant M, Bywater T, Bowyer-Crane C *et al*: Born in Bradford's Better Start: an experimental birth cohort study to evaluate the impact of early life interventions. *BMC Public Health* 2016, 16(1):711.

14. McEachan RRC, Dickerson J, Bridges S *et al.* The Born in Bradford COVID-19 Research Study: Protocol for an adaptive mixed methods research study to gather actionable intelligence on the impact of COVID-19 on health inequalities amongst families living in Bradford [version 1; peer review: 3 approved]. *Wellcome Open Res* 2020, **5**:191 (https://doi.org/10.12688/wellcomeopenres.16129.1)

15. Dickerson J, Kelly B, Lockyer B *et al.* Experiences of lockdown during the Covid-19 pandemic: descriptive findings from a survey of families in the Born in Bradford study [version 2; peer review: 2 approved]. *Wellcome Open Res* 2021, **5**:228 (https://doi.org/10.12688/wellcomeopenres.16317.2)

16. Kroenke K, Strine TW, Spitzer RL, Williams JBW, Berry JT, Mokdad AH: The PHQ-8 as a measure of current depression in the general population. *Journal of Affective Disorders* 2009, 114(1):163-173.

For peer review only - http://bmjopen.bmj.com/site/about/guidelines.xhtml

17. Löwe B, Decker O, Müller S, Brähler E, Schellberg D, Herzog W, Herzberg PY: Validation and Standardization of the Generalized Anxiety Disorder Screener (GAD-7) in the General Population. *Medical Care* 2008, 46(3):266-274.

18. Husain N, Waheed W, Tomenson B, Creed F. The validation of personal health questionnaire amongst people of Pakistani family origin living in the United Kingdom. Journal of Affective Disorders 2007, 97: 261-264.

19. McMillan D, Gilbody S, Richards D. Defining successful treatment outcome in depression using the PHQ-9: A comparison of methods. J. Affective Disorders. 2010: 127; 122–129

20. VanderWeele, T. J., & Robinson, W. R. On the causal interpretation of race in regressions adjusting for confounding and mediating variables. *Epidemiology.* 2014: *25*(4), 473–484.

21. StataCorp. 2017. Stata Statistical Software: Release 15. College Station, TX: StataCorp LLC.

22. Braun V, Clarke V: Using thematic analysis in psychology. *Qualitative Research in Psychology* 2006, 3(2):77-101.

23. Vizard T, Sadler K, Ford T, Newlove-Delgado T, McManus S, Marcheselli F, Davis J, Williams T, Leach C, Mandalia D, Cartwright C. Mental Health of Children and Young People in England, 2020. Health and Social Care Information Centre 2020. https://files.digital.nhs.uk/AF/AECD6B/mhcyp_2020_rep_v2.pdf

24. Prady SL, Pickett KE, Petherick ES, Gilbody S, Croudace T, Mason D, Sheldon TA, Wright J. (2016). Evaluation of ethnic disparities in detection of depression and anxiety in primary care during the maternal period: combined analysis of routine and cohort data. The British journal of psychiatry, 208(5), 453-61. DOI 10.1192/bjp.bp.114.158832. PMID 26795424

25. Prady SL, Pickett KE, Gilbody S, Petherick ES, Mason D, Sheldon TA, and Wright J (2016). Variation and ethnic inequalities in treatment of common mental disorders before, during and after pregnancy: combined analysis of routine and research data in the Born in Bradford cohort. BMC Psychiatry, 16(99), DOI 10.1186/s12888-016-0805-x PMID 27071711

26. Power M, Uphoff N, Stewart-Knox B et al., Food insecurity and socio-demographic characteristics in two UK ethnic groups: an analysis of women in the Born in Bradford cohort. *Journal of Public Health*, 2017. **40**(1): p. 32-40.

27. Yang T.C, Sahota P, Pickett K, Bryant M. Association of food security status with overweight and dietary intake: exploration of White British and Pakistani-origin families in the Born in Bradford cohort. *Nutrition Journal*, 2018. **17**(1): p. 48.

For peer review only - http://bmjopen.bmj.com/site/about/guidelines.xhtml

FIGURE LEGENDS

Figure 1: PHQ-8 at pre-Covid19 and Covid19 lockdown survey with change between categories

Note: This graph presents findings from participants who had complete pre-Covid19 and Covid19 lockdown PHQ-8 scores (n=1730)

Figure 2: GAD-7 at pre-Covid19 and Covid19 lockdown survey with change between categories

Note: This graph presents findings from participants who had complete pre-Covid19 and Covid19 lockdown GAD-7 scores (n=1634)

For peer review only

For peer review only - http://bmjopen.bmj.com/site/about/guidelines.xhtml

Table 1: Sample Characteristics of Mothers who responded to the BiB Covid-19 survey (n=1860)

**Financial security**		
Living comfortably	354	20% (18%-22%)
Doing alright	766	42% (40%-45%)
Just about getting by	462	26% (24%-28%)
Finding it quite/ very difficult	223	12% (11%-14%)
Missing	55	
**Change in financial status (compared to 3 months prior, as asked in lockdown survey)**		
Better off	166	10% (8%-11%)
About the same	989	58% (55%-60%)
Worse off	562	33% (31%-35%)
Missing	143	
**Index of Multiple Deprivation 2019 Quintile**		
1: Most Deprived	1,211	65% (63%-67%)
2	390	21% (19%-23%)
3	139	7% (6%-9%)
4	88	5% (4%-6%)
5: Least Deprived	29	2% (1%-2%)
Missing	3	
**Employment status of main earner**		
Employed - working from home	393	22% (20%-24%)
Employed: going into work	567	32% (30%-34%)
On furlough	271	15% (14%-17%)
Self-employed: working	147	8% (7%-10%)
Self-employed: not working	200	11% (10%-13%)
Unemployed	208	12% (10%-13%)
Missing	74	
**Whether respondent is a keyworker**		
No	1,259	68% (66%-71%)
Yes	579	32% (29%-34%)
Missing	22	
**Whether anyone in household is clinically vulnerable to Covid-19**		
No	1,419	77% (75%-79%)
Yes	426	23% (21%-25%)
Missing	15	
**Whether anyone in household is currently/ has previously self-isolated**		
No	1,331	72% (70%-74%)
Yes	508	28% (26%-30%)
Missing	21	
**Number of children in household**		
One	387	21% (19%-23%)
Two	699	38% (35%-40%)
Three	451	24% (22%-26%)
Four or more	323	17% (16%-19%)
**Total household size**		

For peer review only - http://bmjopen.bmj.com/site/about/guidelines.xhtml

Two/ Three	321	17% (16%-19%)
Four/ Five	943	51% (49%-53%)
Six or more	583	32% (29%-34%)
Missing	13	
**Whether single parent**		
No	1,604	88% (86%-89%)
Yes	222	12% (11%-14%)
Missing	34	
**Quality of relationship with partner (for those married or in a relationship, n=1604)**		
Excellent	783	51% (48%-53%)
Good	598	39% (36%-41%)
Average	129	8% (7%-10%)
Poor	23	1% (1%-2%)
Very poor	10	1% (0%-1%)
Missing	61	
**Loneliness: How often felt lonely in the past week**		
None, or almost none of the time	998	57% (55%-60%)
Some of the time	555	32% (30%-34%)
Most of the time	131	8% (6%-9%)
All, or almost all of the time	56	3% (2%-4%)
Missing	120	
**Social support: how easy to get help from friends/ neighbours if needed**		
Very difficult	101	6% (5%-7%)
Difficult	146	8% (7%-9%)
Possible	529	29% (27%-31%)
Easy	529	29% (27%-31%)
Very easy	513	28% (26%-30%)
Missing	42	
**Food security: How often is it true that food does not last**		
Never	1,379	79% (77%-81%)
Sometimes	279	16% (14%-18%)
Often	91	5% (4%-6%)
Missing	111	
**Housing security: Whether worried about eviction/ repossession**		
Strongly disagree or disagree	1,250	71% (68%-73%)
Neither	328	19% (17%-20%)
Strongly agree/ agree	194	11% (10%-12%)
Missing	88	
**Job security: Whether expect main earner to still have job in 12 months (for those currently employed (n=1652))**		
No	77	5% (4%-6%)
Yes	979	61% (59%-64%)
Don't know	537	34% (31%-36%)
Missing	59	
**Housing conditions: Whether damp in house**		
No	1,307	71% (69%-73%)
Yes	534	29% (27%-31%)
Missing	19	

For peer review only

**Frequency of physical activity currently**		
Never	239	13% (12%-15%)
1 or 2 days a week	489	27% (25%-29%)
Most days	516	28% (26%-30%)
Every day	586	32% (30%-34%)
Missing	30	
**Change in frequency of physical activity since lockdown**		
Less	766	48% (46%-51%)
About the	408	26% (24%-28%)
More	419	26% (24%-29%)
Missing	267	

For peer review only

For peer review only - http://bmjopen.bmj.com/site/about/guidelines.xhtml

Table 2: Depression and Anxiety Scores in the Pre-Covid19 and Covid-19 surveys (n=1860).

	Pre-Covid19 survey		Covid-19 survey	
	N	Percentage (95% CI)	N	Percentage (95% CI)
**Depression category (PHQ-8)**				
None (PHQ-8 score 0 to 4)	1187	65% (63%-68%)	1001	56% (54%-59%)
Mild (PHQ-8 score 5 to 9)	414	23% (21%-25%)	425	24% (22%-26%)
Moderate (PHQ-8 score 10 to 14)	135	7% (6%-9%)	199	11% (10%-13%)
Moderately severe/ severe (PHQ-8 score 15 to 24)	77	4% (3%-5%)	150	8% (7%-10%)
Missing	47		85	
**Anxiety category (GAD7)**				
None (GAD-7 score 0 to 4)	1280	75% (72%-77%)	1075	61% (58%-63%)
Mild (GAD-7 score 5 to 9)	270	16% (14%-18%)	408	23% (21%-25%)
Moderate (GAD-7 score 10 to 14)	100	6% (5%-7%)	165	9% (8%-11%)
Severe (GAD-7 score 15 to 21)	67	4% (3%-5%)	124	7% (6%-8%)
Missing	143		88	

For peer review only

Table 3: Odds ratios (with 95% CI) from unadjusted logistic regression models for an increase of 5 points or more on PHQ-8 and GAD-7 between pre-Covid19 and Covid-19 lockdown survey.

	PHQ-8 increase >= 5			GAD-7 increase >= 5		
**Exposure**	**Odds Ratio**	**Low 95% CI**	**High 95% CI**	**Odds Ratio**	**Low 95% CI**	**High 95% CI**
**Ethnicity (Reference: White British)**						
Pakistani heritage	0.88	0.68	1.15	0.94	0.72	1.23
**Age group (Reference: Under 30 years)**						
30 to 34 years	1.01	0.66	1.54	0.90	0.57	1.42
35 to 39 years	0.89	0.59	1.35	0.99	0.64	1.53
40 to 44 years	1.04	0.69	1.58	0.88	0.57	1.38
45 years plus	0.92	0.59	1.45	1.02	0.64	1.63
**Financial security at pre-Covid19 survey (Reference: Living comfortably)**						
Doing alright	1.07	0.81	1.42	1.19	0.89	1.59
Just about getting by	1.72	1.23	2.40	1.66	1.17	2.35
Finding it quite/ very difficult	1.70	1.03	2.80	2.39	1.44	3.98
**Financial security at lockdown survey (Reference: Living comfortably)**						
Doing alright	1.74	1.18	2.56	1.47	1.00	2.17
Just about getting by	3.52	2.36	5.26	3.20	2.15	4.76
Finding it quite/ very difficult	6.23	3.96	9.80	6.03	3.82	9.51
**Change in financial status compared to 3 months ago (Reference: Better off)**						
About the same	0.65	0.43	0.98	0.88	0.55	1.40
Worse off	1.36	0.89	2.01	1.99	1.25	3.19
**Index of Multiple Deprivation 2019 Quintile (Reference: 1 Most deprived fifth)**						
2	0.90	0.67	1.21	1.22	0.92	1.63
3	0.91	0.58	1.41	0.87	0.55	1.39
4	0.76	0.43	1.34	0.98	0.57	1.68
5 Least deprived fifth	0.85	0.34	2.13	0.99	0.39	2.48
Per 1 fifth increase in lower deprivation	1.07	0.94	1.25	1.00	0.88	1.13
**Employment status of main earner (Reference: Employed and working from home)**						
Employed: going into work	1.18	0.84	1.65	1.20	0.85	1.69
On furlough	1.19	0.79	1.79	1.29	0.85	1.94
Self-employed: working	0.90	0.53	1.51	0.92	0.55	1.57
Self-employed: not working	1.49	0.97	2.27	1.31	0.84	2.04
Unemployed	2.03	1.34	3.09	2.23	1.44	3.44

For peer review only

**Whether respondent is a keyworker**						
**(Reference: No)**						
Yes	0.99	0.77	1.27	1.05	0.82	1.35
**Whether anyone in household is clinically vulnerable to Covid-19**						
**(Reference: No)**						
Yes	1.68	1.29	2.19	1.53	1.17	2.01
**Whether anyone in household is currently/ has previously self-isolated**						
**(Reference: No)**						
Yes	1.59	1.24	2.04	1.48	1.14	1.92
**Number of children in household**						
**(Reference: One)**						
Two	1.20	0.87	1.67	1.05	0.76	1.45
Three	1.39	0.98	1.98	1.20	0.84	1.71
Four or more	1.12	0.76	1.65	0.93	0.62	1.39
**Total household size**						
**(Reference: Less than four)**						
Four or Five	0.73	0.53	0.99	0.95	0.69	1.32
Six or more	0.72	0.51	1.00	0.90	0.63	1.29
**Whether single parent**						
**(Reference: No)**						
Yes	1.66	1.18	2.33	1.28	0.89	1.85
**Quality of relationship with partner**						
**(Reference: Excellent)**						
Good	1.93	1.45	2.57	1.82	1.35	2.44
Average to poor	3.64	2.44	5.43	5.07	3.37	7.62
**Loneliness**						
**(Reference: Not lonely)**						
Lonely most/ all of the time	8.37	5.70	12.27	8.50	5.71	12.65
**Social support**						
**(Reference: Easy to get support)**						
Not easy to get support	2.25	1.78	2.86	2.13	1.67	2.73
**Food security**						
**(Reference: Secure)**						
Insecure - Often true that food does not last	3.33	2.09	5.28	3.46	2.15	5.58
**Housing security**						
**(Reference: Secure)**						
Insecure - Worried about eviction/ repossession	3.29	2.36	4.58	3.00	2.11	4.25
**Job security: whether expect main earner to still have job in 12 months**						
**(Reference: Yes)**						

For peer review only - http://bmjopen.bmj.com/site/about/guidelines.xhtml

For peer review only

No	0.84	0.49	1.43	0.96	0.56	1.64
**Housing conditions (Reference: No damp in house)**						
Damp in house	1.85	1.45	2.38	1.57	1.21	2.03
**Frequency of physical activity (Reference: Every day)**						
Most days	1.58	1.15	2.18	1.67	1.21	2.31
1 or 2 days a week	1.93	1.40	2.67	1.67	1.20	2.31
Never	3.13	2.15	4.56	2.55	1.72	3.78
**Change in frequency of physical activity since lockdown (Reference: More than before)**						
About the same	0.66	0.45	0.97	0.82	0.56	1.21
Less than before	1.27	0.94	1.72	1.43	1.04	1.95
**Time between baseline and Covid-19 lockdown survey**						
Time (in Months)	1.00	0.99	1.01	1.00	0.99	1.01
**Cohort Source (Reference: BiBGU)**						
BiBBS	0.84	0.64	1.10	0.84	0.63	1.12

For peer review only

For peer review only - http://bmjopen.bmj.com/site/about/guidelines.xhtml

Table 4a: Odds ratios (with 95% CI) from unadjusted logistic regression models for an increase of 5 points or more on PHQ-8 between pre-Covid19 and Covid-19 lockdown survey for White British and Pakistani heritage mothers.

	White British			Pakistani Heritage		
**Exposure**	**Odds Ratio**	**Low 95% CI**	**High 95% CI**	**Odds Ratio**	**Low 95% CI**	**High 95% CI**
**Financial security (Reference: Living comfortably)**						
Doing alright	2.00	1.12	3.56	1.70	0.88	3.27
Just about getting by	3.98	2.15	7.38	3.03	1.56	5.88
Finding it quite/ very difficult	12.14	5.15	28.60	5.79	2.90	11.56
**Food security (Reference: Secure)**						
Insecure	3.73	1.58	8.77	4.13	2.08	8.17
**Housing security (Reference: Secure)**						
Insecure	4.14	2.07	8.29	3.20	1.99	5.16
**Loneliness (Reference: not lonely)**						
Lonely most/ all of the time	7.17	3.66	14.01	11.22	6.45	19.53
**Social support (Reference: Easy to get support)**						
Not Easy	2.08	1.39	3.10	2.20	1.54	3.14
**Quality of relationship with partner (Reference: Excellent)**						
Good	1.68	1.02	2.77	1.76	1.15	2.68
Average to very poor	2.61	1.31	5.20	4.91	2.78	8.67
**Total household size (Reference: Less than four)**						
Four or Five	0.69	0.44	1.07	0.61	0.35	1.05
Six or more	0.91	0.47	1.73	0.54	0.31	0.94
**Frequency of physical activity (Reference: Every day)**						
Never	5.54	2.69	11.43	3.34	1.88	5.92
1 or 2 days a week	1.97	1.15	3.37	2.05	1.22	3.47
Most days a week	1.33	0.81	2.17	2.08	1.21	3.58

For peer review only

Table 4b: Odds ratios (with 95% CI) from unadjusted logistic regression models for an increase of 5 points or more on GAD-7 between pre-Covid19 and Covid-19 lockdown survey for White British and Pakistani heritage mothers.

	White British			Pakistani Heritage		
**Exposure**	**Odds Ratio**	**Low 95% CI**	**High 95% CI**	**Odds Ratio**	**Low 95% CI**	**High 95% CI**
**Financial security (Reference: Living comfortably)**						
Doing alright	1.70	0.97	3.00	0.93	0.50	1.74
Just about getting by	2.82	1.53	5.21	2.55	1.39	4.68
Finding it quite/ very difficult	7.69	3.43	17.26	4.35	2.27	8.30
**Food security (Reference: Secure)**						
Insecure	3.59	1.47	8.7	4.96	2.49	9.87
**Housing security (Reference: Secure)**						
Insecure	3.32	1.67	6.57	3.07	1.84	5.10
**Loneliness (Reference: not lonely)**						
Lonely	5.90	3.03	11.47	11.27	6.16	20.56
**Social support (Reference: Easy to get support)**						
Not Easy	2.33	1.55	3.51	2.09	1.45	3.01
**Quality of relationship with partner (Reference: Excellent)**						
Good	1.54	0.93	2.55	1.88	1.22	2.89
Average to very poor	4.39	2.26	8.53	4.99	2.72	9.14
**Total household size (Reference: Less than four)**						
Four or Five	1.01	0.63	1.62	0.64	0.36	1.14
Six or more	1.13	0.58	2.20	0.61	0.34	1.07
**Frequency of physical activity (Reference: Every day)**						
Never	3.90	1.82	8.36	2.50	1.40	4.45
1 or 2 days a week	1.52	0.88	2.63	1.61	0.97	2.70
Most days a week	1.45	0.90	2.34	1.86	1.09	3.18

For peer review only

For peer review only

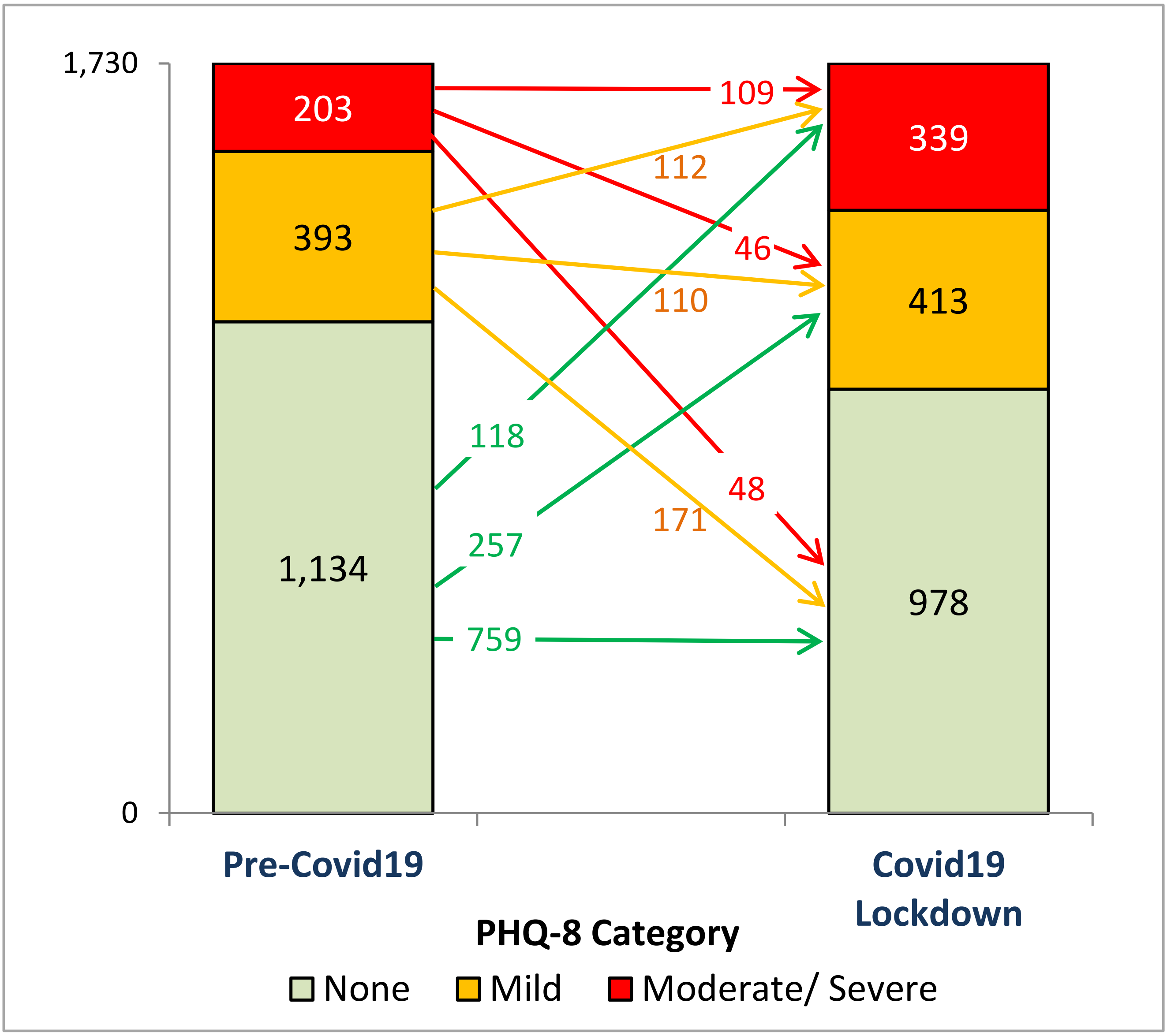
1,730
0
203
393
1,134
339
413
978
109
112
46
110
118
48
171
257
759
Pre-Covid19
Covid19 Lockdown
PHQ-8 Category
None
Mild
Moderate/ Severe

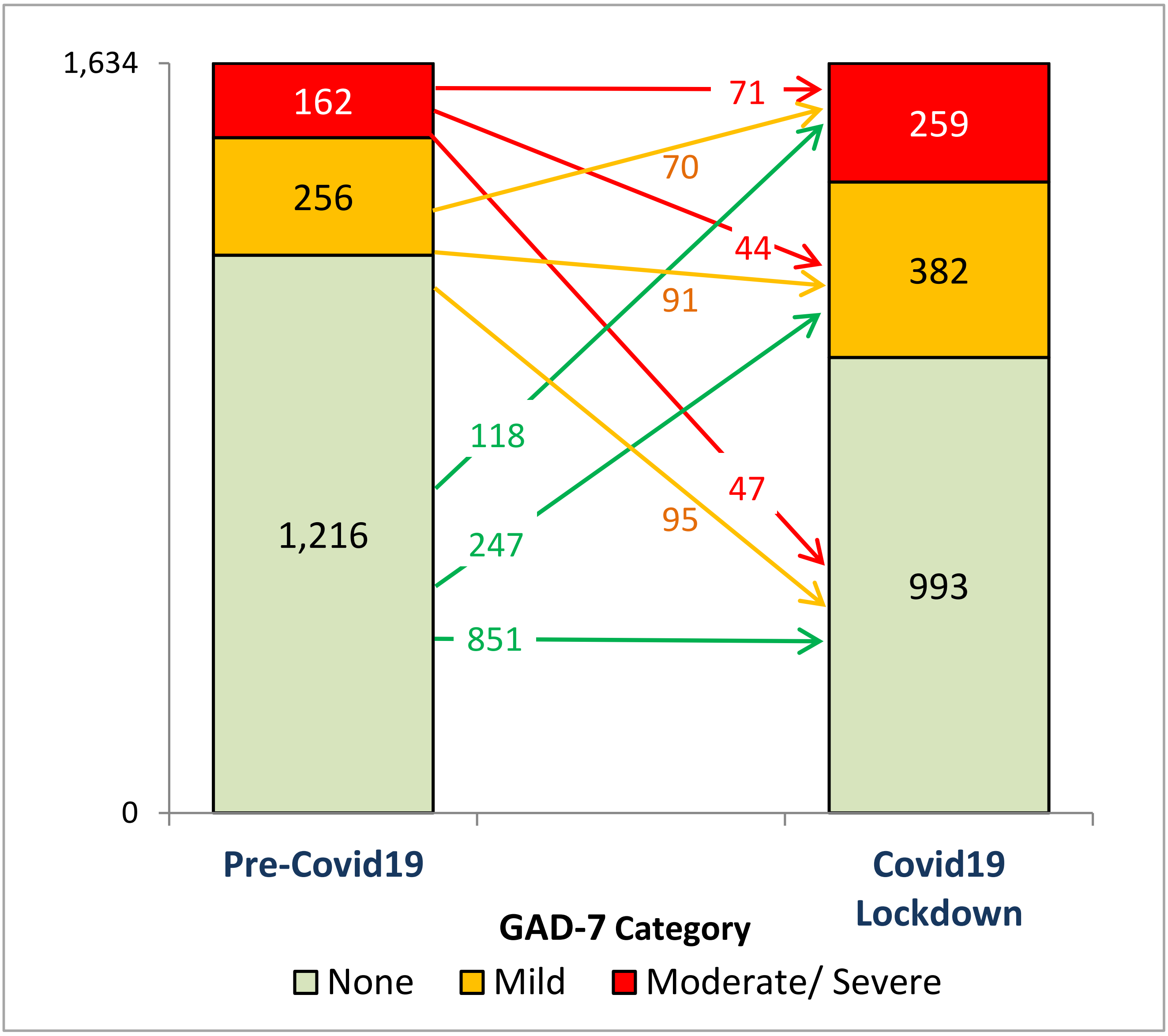
1,634
0
162
256
1,216
259
382
993
71
70
44
91
118
47
95
247
851
Pre-Covid19
Covid19 Lockdown
GAD-7 Category
None
Mild
Moderate/ Severe

Supplementary Table 1: The sample characteristics of those invited to complete the Covid-19 survey, by survey completion status.

**Age**	Completed survey (n=1860)	Not completed survey (n=4722)	Total eligible (n=6866)
Under 30 yrs.	224 (12%)	751 (16%)	963 (14%)
30 to 34 yrs.	396 (21%)	1182 (25%)	1600 (24%)
35 to 39 yrs.	516 (28%)	1367 (29%)	1939 (29%)
40 to 44 yrs.	423 (23%)	892 (19%)	1395 (21%)
45 yrs. plus	301 (16%)	495 (11%)	887 (13%)
Missing	-	35	82
**Ethnicity***			
White British	613 (34%)	821 (18%)	1527 (23%)
Pakistani Heritage	877 (48%)	2921 (64%)	3843 (59%)
Other	320 (18%)	803 (18%)	1148 (18%)
Missing	50	177	348
**Depression (PHQ8)**			
None	1187 (66%)	2594 (64%)	3774 (64%)
Mild	414 (23%)	945 (23%)	1345 (23%)
Moderate	135 (7%)	346 (8%)	475 (8%)
Moderately severe	58 (3%)	134 (3%)	193 (3%)
Severe	19 (1%)	64 (2%)	83 (1%)
Missing	47	639	996
**Anxiety (GAD7)**			
None	1280 (75%)	2720 (72%)	3991 (73%)
Mild	270 (16%)	646 (17%)	908 (17%)
Moderate	100 (6%)	224 (6%)	322 (6%)
Severe	67 (4%)	166 (4%)	234 (4%)
Missing	143	966	1,411

**Note: The ethnicity representativeness is skewed by the participants in BiBGU – see reference 15 for explanation*

For peer review only

Supplementary Table 2: Change in PhQ-8 and Gad-7 categories from Pre-Covid19 baseline to Covid-19 lockdown survey.

**Pre-Covid19 PHQ8 Category**	**PHQ8 at Covid-19 Lockdown Survey** None	Mild	Moderate/ Severe	Missing	Total
None	759 (67%)	257 (23%)	118 (10%)	53	1187 (100%)
Mild	171 (44%)	110 (28%)	112 (28%)	21	414 (100%)
Moderate/ Severe	48 (24%)	46 (23%)	109 (54%)	9	212 (100%)
Missing	23	12	10	2	47 (100%)
Total	978	413	339	85	1860 (100%)

Pre-Covid19 GAD7 Category	GAD7 at Covid-19 Lockdown Survey None	Mild	Moderate/ Severe	Missing	Total
None	851 (70%)	247 (20%)	118 (10%)	64	1280 (100%)
Mild	95 (37%)	91 (36%)	70 (27%)	14	270 (100%)
Moderate/ Severe	47 (29%)	44 (27%)	71 (44%)	5	167 (100%)
Missing	82	26	30	5	143 (100%)
Total	993	382	259	88	1860 (100%)

RED: category worse in lockdown compared to baseline; AMBER: category stayed the same; GREEN: category improved in lockdown compared to baseline

For peer review only

BMJ Open

"When will this end? Will it end?" The impact of the March-June 2020 UK Covid-19 lockdown response on mental health: a longitudinal survey of mothers in the Born in Bradford study.

Journal:	*BMJ Open*
Manuscript ID	bmjopen-2020-047748.R2
Article Type:	Original research
Date Submitted by the Author:	11-Oct-2021
Complete List of Authors:	Dickerson, Josie; Bradford Institute for Health Research<br>Kelly, Brian; Bradford Institute for Health Research<br>Lockyer, Bridget; Bradford Institute for Health Research<br>Bridges, Sally; Bradford Institute for Health Research<br>Cartwright, Christopher; Bradford Institute for Health Research<br>Willan, Kathryn ; Bradford Institute for Health Research<br>Shire, Katy; Bradford Institute for Health Research<br>Crossley, Kirsty; Bradford Institute for Health Research<br>Bryant, Maria; University of York, Health Sciences<br>Siddiqi, Najma; University of York, Health Sciences; Hull York Medical School<br>Sheldon, T; Bradford Institute for Health Research; Queen Mary University of London Barts and The London School of Medicine and Dentistry, Institute of Population Health Sciences<br>Lawlor, Deborah; MRC Integrative Epidemiology Unit, MRC Integrative Epidemiology Unit at The University of Bristol; University of Bristol Medical School, Population Health Science<br>Wright, John; Bradford Institute for Health Research<br>McEachan, Rosemary; Bradford Institute for Health Research; University of Bradford Faculty of Life Sciences<br>Pickett, Kate; University of York, Health Sciences
**Primary Subject Heading**:	Public health
Secondary Subject Heading:	Mental health, Public health
Keywords:	COVID-19, MENTAL HEALTH, Depression & mood disorders < PSYCHIATRY, Anxiety disorders < PSYCHIATRY, PUBLIC HEALTH

SCHOLARONE™
Manuscripts

I, the Submitting Author has the right to grant and does grant on behalf of all authors of the Work (as defined in the below author licence), an exclusive licence and/or a non-exclusive licence for contributions from authors who are: i) UK Crown employees; ii) where BMJ has agreed a CC-BY licence shall apply, and/or iii) in accordance with the terms applicable for US Federal Government officers or employees acting as part of their official duties; on a worldwide, perpetual, irrevocable, royalty-free basis to BMJ Publishing Group Ltd ("BMJ") its licensees and where the relevant Journal is co-owned by BMJ to the co-owners of the Journal, to publish the Work in this journal and any other BMJ products and to exploit all rights, as set out in our licence.

The Submitting Author accepts and understands that any supply made under these terms is made by BMJ to the Submitting Author unless you are acting as an employee on behalf of your employer or a postgraduate student of an affiliated institution which is paying any applicable article publishing charge ("APC") for Open Access articles. Where the Submitting Author wishes to make the Work available on an Open Access basis (and intends to pay the relevant APC), the terms of reuse of such Open Access shall be governed by a Creative Commons licence – details of these licences and which Creative Commons licence will apply to this Work are set out in our licence referred to above.

Other than as permitted in any relevant BMJ Author's Self Archiving Policies, I confirm this Work has not been accepted for publication elsewhere, is not being considered for publication elsewhere and does not duplicate material already published. I confirm all authors consent to publication of this Work and authorise the granting of this licence.

For peer review only

"When will this end? Will it end?" The impact of the March-June 2020 UK Covid-19 lockdown response on mental health: a longitudinal survey of mothers in the Born in Bradford study.

Josie Dickerson[1], Brian Kelly[1], Bridget Lockyer[1], Sally Bridges[1], Christopher Cartwright[1], Kathryn Willan[1], Katy Shire[1], Kirsty Crossley[1], Maria Bryant[1,2] Najma Siddiqi[3], Trevor A Sheldon[1,4], Deborah A Lawlor[5,6,7], John Wright[1], Rosemary RC McEachan[1,8], Kate E Pickett[1,2] on behalf of the Bradford Institute for Health Research Covid-19 Scientific Advisory Group (https://www.bradfordresearch.nhs.uk/c-sag/).

Corresponding author:

Josie Dickerson, Bradford Institute for Health Research, Bradford Teaching Hospitals NHS Foundation Trust, Bradford Royal Infirmary, Duckworth Lane, Bradford, BD9 6RJ, UK.

Email: josie.dickerson@bthft.nhs.uk

Telephone: 01274 383916

Addresses

[1] Bradford Institute for Health Research, Bradford Teaching Hospitals NHS Foundation Trust, Bradford Royal Infirmary, Duckworth Lane, Bradford, UK.

[2] Department of Health Sciences, University of York, Department of Health Sciences, Seebohm Rowntree Building, University of York, Heslington, York, UK.

[3] The Hull York Medical School, University of York, Heslington York, UK

[4] Institute of Population Health Sciences, Barts and The London School of Medicine and Dentistry, Queen Mary University London, Yvonne Carter Building, 58 Turner Street, London, UK

[5] MRC Integrative Epidemiology Unit at the University of Bristol, Oakfield House, Oakfield Grove, Bristol, , UK

[6] Population Health Science, Bristol Medical School, University of Bristol, UK

[7] Bristol National Institute for Health Research Biomedical Research Centre, UK

[8] Faculty of Life Sciences and Health Studies, University of Bradford, Richmond Rd, Bradford, UK

KEYWORDS

Covid-19; mental health; depression; anxiety; health inequalities; ethnic minorities

WORD COUNT: 5,231

For peer review only

For peer review only - http://bmjopen.bmj.com/site/about/guidelines.xhtml

ABSTRACT

Objectives To explore clinically important increases in depression/anxiety from before to during the first UK Covid-19 lockdown and factors related to this change, with a particular focus on ethnic differences.

Design Pre-Covid-19 and lockdown surveys nested within two longitudinal Born in Bradford cohort studies.

Participants 1,860 mothers with a child aged 0-5 or 9-13, 48% Pakistani heritage

Main outcome measures Odds ratios (OR) for a clinically important increase (5 points or more) in depression (PHQ-8) and anxiety (GAD-7) in unadjusted regression analyses, repeated with exposures of interest separated by ethnicity to look for differences in magnitude of associations, and lived experience of mothers captured in open text questions.

Results The number of women reporting clinically important depression/anxiety increased from 11% to 20% [10-13%;18-22%] and 10% to 16% [8-11%; 15-18%]) respectively. Increases in depression/anxiety were associated with: loneliness (OR: 8.37, [5.70-12.27]; 8.50, [5.71-12.65] respectively); financial (6.23, [3.96-9.80]; 6.03, [3.82-9.51]); food (3.33 [2.09-5.28]; 3.46 [2.15-5.58]); and housing insecurity (3.29 [2.36-4.58]; 3.0 [2.11-4.25]); a lack of physical activity (3.13 [2.15-4.56]; 2.55 [1.72-3.78]); and a poor partner relationship (3.6 [2.44-5.43]; 5.1 [3.37-7.62]. The magnitude of associations between key exposures and worsening mental health varied between ethnic groups.
Responses to open text questions illustrated a complex inter-play of challenges contributing to mental ill health including: acute health anxieties; the mental load of managing multiple responsibilities; loss of social support and coping strategies; pressures of financial and employment insecurity; and being unable to switch off from the pandemic.

Conclusions Mental ill health has worsened for many during the Covid-19 lockdown, particularly in those who are lonely and economically insecure. The magnitude of associations between key exposures and worsening mental health varied between ethnic groups. Mental health problems may have longer term consequences for public health and interventions that address the potential causes are needed.

STRENGTHS AND LIMITATIONS OF THIS STUDY

- Three key longitudinal studies have highlighted that the Covid-19 pandemic and lockdowns have had a negative impact on mental health, particular in younger adults, women and those from low socio-economic circumstances, but with participants of predominantly White European ethnicity.

- The Born in Bradford research programme offers a unique opportunity to investigate the impact of Covid-19 lockdown on mental health in a deprived and ethnically diverse population in whom mental ill health is often reported to be more prevalent.

- This is a longitudinal study containing linked data collected before the Covid-19 pandemic and during the March-June 2020 lockdown which has allowed us to explore change over that time period in a highly ethnically diverse population, the majority of whom live in the most deprived centiles in the UK.

- Respondents in this study were mothers of children aged 0-5 and/or 9-13 which may limit the wider generalisability, though our findings are broadly similar (in prevalence and associations) to those from another longitudinal study that included adult men and women.

- We are not aware of other studies that have explored longitudinal change in mental health from before to during the Covid-19 lockdown in a similar ethnically diverse and deprived population.

For peer review only - http://bmjopen.bmj.com/site/about/guidelines.xhtml

Introduction

There is growing concern that the 'lockdown' measures to control the spread of the Covid-19 pandemic[1,2] have had unintended consequences including an increase in mental ill health. Several studies since the Covid-19 pandemic began have reported high levels of depression and anxiety[3-7] in the UK. However, some of these surveys are either cross-sectional or longitudinal *within* the lockdown period[6-7] with only three studies[3,4,5] comparing mental health in the pre-Covid-19 period to mental health during the Covid-19 lockdown. These studies consistently found worsening mental health in younger adults and those who were financially insecure. Other associations were reported in those who: had a disability[3]; pre-existing mental and physical health conditions[4]; were living alone[4]; in parents of young children[5]; and in women[4,5].

In these studies, most participants were of white European origin and the larger of the studies was in a relatively affluent population[4]. Mental ill health is often reported as being more prevalent in people from ethnic minorities and the socially and economically disadvantaged[8,9], but no longitudinal research to date has investigated the impact of Covid-19 lockdown on mental health in these populations[10].

We were able to explore these questions in depth by building on the Born in Bradford research programme which includes two longitudinal birth cohorts of ethnically diverse families living in the city of Bradford, many in deprived circumstances. These cohorts have recent in-depth information on the demographics, socioeconomic status and mental health of mothers before the Covid-19 pandemic,[11-13] as well as mental health during the March-June 2020 Covid-19 lockdown[14], and so offer a unique opportunity to assess the impact of Covid-19 and lockdown longitudinally in a deprived and ethnically diverse population.

We used the data to:

- Describe the changes in the prevalence of depression and anxiety in mothers living in Bradford from before the Covid-19 pandemic to during the March-June 2020 Covid-19 lockdown.
    - Identify variables associated with a clinically important increase in mental ill health in this population, to identify vulnerable groups that may need additional support in the recovery from the pandemic.
    - Explore whether there is a difference in the magnitude of the associations of key exposures with an increase in clinically important mental ill health by ethnicity (Pakistani heritage compared to White British ethnicity).
    - To explore mothers' lived experience during the March-June 2020 Covid-19 lockdown by assessing the frequency of worries and concerns relating to mental ill health obtained through free text responses to open questions.

Methods

Study Design

A longitudinal study using data collected at two time points, before and during the March-June 2020 Covid-19 lockdown, from mothers who participated in one of two prospective birth cohort studies in Bradford: Born in Bradford's Growing Up (BiBGU) study, with parents of children currently aged 9-13[11,12] and Born in Bradford's Better Start (BiBBS), with parents of children currently aged 0-5.[13]

Patient and Public Involvement

Born in Bradford is a 'people powered' research study; the local community were consulted to identify key research priorities during the March-June 2020 Covid-19 lockdown. This included consultation with key community groups, seldom-heard communities and local policy and decision makers to ensure that the focus of the research was relevant to local needs. The Covid-19 survey and recruitment approach were tested through our established research advisory groups. The findings of the study were also shared with these groups to enhance interpretation and ensure useful dissemination back to the community[14].

Consent

For peer review only - http://bmjopen.bmj.com/site/about/guidelines.xhtml

Participants had previously consented for their research data, and routinely collected health and education data, to be used for research. For the Covid-19 survey, verbal consent was taken for questionnaires completed over the phone and implied consent was assumed for all questionnaires completed via post or online.

Data Collection

Full details of the data collection of the March-June 2020 Covid-19 survey can be found elsewhere[14,15]. In summary:

Participants were recruited using a combination of emails, text and phone with a follow-up postal survey, and in their main language wherever possible.

a) Pre-Covid-19 data

Pre-Covid-19 data for BiBGU participants were derived from two sources: a) participant ethnicity and age were collected during pregnancy (2007-2011); [11] b) recent follow-up data on mental health (collected between 24th June 2017 and 12th March 2020).[12] Pre-Covid-19 data for BiBBS participants were taken from data collected during pregnancy (6th January 2016 and 8th February 2020).[13] The median time since most recent pre-Covid-19 data collection was 15 months (range 1 to 35) for BiBGU and 29 months (range 2 to 52) for BiBBS.

b) March-June 2020 Covid-19 lockdown data collection:

Mental health was measured using the PHQ-8[16] for depression and the GAD-7 for anxiety.[17] These are widely used measures of the severity of symptoms of depression and anxiety that have been validated in the general population, [16,17] and in many ethnic minorities including UK residents of Pakistani heritage.[18] Information was also collected on: household circumstances; family relationships; social support and loneliness; financial, employment, housing and food insecurity; physical health. This contextual information was captured in self-reported questions administered in the Covid-19 lockdown survey; details of variables used are in Table 1 and our protocol paper[14]. Information on the participants' lived experience during lockdown were captured using free text questions that asked "what are your three biggest worries at the moment?" and "has anything been easier or more enjoyable in lockdown?"

Data Preparation

To describe the changes in prevalence of depression and anxiety from before the Covid-19 pandemic to during the Covid-19 lockdown we derived a categorical variable for each PHQ-8 and GAD-7 score based on the standard clinical scoring classification: 0 to 4 no depression, 5 to 9 mild depression, 10-24 moderate-severe depression; 0 to 4 no anxiety, 5 to 9 mild anxiety, 10 to 21 moderate-severe anxiety.[16,17] and then collapsed moderate, moderately severe and severe categories to indicate clinically important symptoms of depression and anxiety. To identify variables associated with an increase of mental ill health we used an increase in PHQ-8 and GAD-7 scores of 5 or more points as an indicator of clinically important change in symptoms. This cut-off was chosen following guidance from previous research and consultation with clinical colleagues[19]. An increase of five points or more would also always result in a change in the categorisation of symptoms (e.g. from none to mild or mild to moderate/severe) whilst also capturing changes in severity within the moderate/severe categories.

Ethnicity was coded using Census 2011 categories and also as 'White British', 'Pakistani Heritage' and 'Other', which included all other groups due to small numbers of a wide range of ethnicities. Given the heterogeneity of the 'Other' ethnic group, the decision was made to not use this group in these analyses, but to focus on comparing those of Pakistani heritage and White British ethnicity.

A number of categories within other explanatory variables were collapsed for analysis. These included: Quality of relationship with partner: Average to poor (comprising of 'average', 'poor' and 'very poor'); Loneliness: Not lonely (comprising of: 'none or almost none of the time' and 'some of the time') and Lonely (comprising of: 'Most of the time' and 'All or almost all of the time'); Social support: Easy to get support (comprising of: 'Very easy' and 'easy'); and Not easy to get support (comprising of: 'Very difficult', 'Difficult'

For peer review only - http://bmjopen.bmj.com/site/about/guidelines.xhtml

and ‘Possible’); Food insecurity: Secure (Comprising of’ never true’ or ‘sometimes true’ that food didn’t last) and Insecure (Comprising of ‘Often true’ that food didn’t last). Housing security: Secure (Comprising: ‘Strongly Disagree’, ‘Disagree’ and ‘Neither disagree or agree’ that I worry about being evicted or having my home repossessed) and Insecure (comprising of ‘Strongly Agree’ or ‘Agree’).

Missing data on measures was small for most variables (Table 1) and was not adjusted for in the analyses.

Data analysis

To describe the changes in the prevalence of depression and anxiety from before to during the pandemic we explored the changes in PHQ-8 and GAD-7 categories using descriptive statistics and presented these visually to elucidate the patterns of both positive and negative changes.

We used descriptive statistics to present results of sample characteristics, including depression and anxiety scores at pre-Covid-19 and Covid-19 lockdown survey time points.

We then modelled the odds ratios associated with an increase in PHQ-8 and GAD-7 scores by five or more points using separate unadjusted logistic regression models for each exposure variable of interest. Exposure variables included in the analyses were identified as indicative of an increase in mental ill-health in the lockdown survey findings reported previously.[15] Pre-Covid-19 PHQ-8 and GAD-7 scores were controlled for in each model.

In order to explore whether or not the magnitude of the association between exposure variables and a clinically important increase in symptoms of depression and anxiety differed between ethnic groups, we repeated the above analyses for Pakistani heritage and White British participants separately. This approach avoids the difficulties inherent in interpreting the ethnicity coefficient in regression models controlling for other variables[20]. All statistical analyses were carried out using Stata 15.[21]

To explore mothers’ lived experience during the March-June 2020 Covid-19 lockdown, the free text responses to open questions were reviewed and themes that related to mental health of the mothers were pulled out. Responses were coded using thematic analysis[22]. The first 100 responses were analysed by one researcher (BL), employing an inductive approach where coding and theme development were driven by the content of the responses. Two codebooks were developed, one for the questions on the three biggest worries and recent challenges during lockdown and another smaller codebook for the question on what had been made more enjoyable and easier during lockdown. Using Microsoft Excel, the remaining responses were then coded by three different researchers in order to test the strength and validity of the codebooks. Through frequent discussion between the researchers about this process, adjustments were made to the original codebooks so that they were reflective of the total responses. The emergent themes relating to mental health were used to illuminate the findings from the quantitative analyses.

Ethics

This study was approved by the HRA and Bradford/Leeds research ethics committee (Substantial amendments to: BiBGU 16/YH/0320 and BiBBS 15/YH/0455)

Results

Study Population

2,144 (28%) of those invited participated in the Covid-19 survey between 10th April and 30th June 2020. Full details of the study population are described elsewhere and demonstrate that the population are broadly representative of the BiBGU and BiBBS cohorts[15]. Of these 2,144 mothers, 1,860 (87%) had complete surveys and linked data from pre-Covid-19 surveys and were used for this study. Of these participants, 1,316 (71%) were in the BiBGU cohort (with a child aged 9-13) and 544 (29%) were in the BiBBS cohort (with a child aged 0-5) and had a mean age of 37.5 years (SD 6.8). Participants were ethnically diverse: 877 (48%) were of Pakistani heritage, 613 (34%) White British and 320 (18%) other ethnic groups; 1,211 (65%) lived in the most deprived quintile of material deprivation in England. Table 1 describes the sample characteristics of the study population. Respondents were representative of the invited cohort on baseline

For peer review only - http://bmjopen.bmj.com/site/about/guidelines.xhtml

(i.e. prior to the Covid-19 pandemic) levels of depression and anxiety. Ethnicity was skewed with more White British and less Pakistani heritage respondents than in the invited sample, although the study sample still offers a large ethnically diverse population (see Supplementary Table 1).

[Table 1 here]

Study Sample Change in Prevalence of Depression and Anxiety (n=1860)

The prevalence of moderate / severe depression increased between the pre-Covid-19 and Covid-19 lockdown surveys (n=1860) from 11% (n=212 [95% Confidence Intervals 10-13%] to 20% (n=349 [18-22%]). The proportion of mothers reporting mild symptoms of depression remained similar; while the proportion of those with no depressive symptoms decreased from 65% (n=1187 [63-68%]) to 56% (n=1001 [54-59%]), (Table 2). The proportion of mothers with moderate/severe anxiety increased from 10% (n=167 [8-11%]) to 16% (n=289 [15-18%). The prevalence of mild anxiety also increased from 16% (n=270 [14-18%]) to 23% (n=408 [21-25%]), whilst the proportion of participants with no anxiety fell from 75% (n=1280 [72-77%]) to 61% (n=1075 [58-63%]) (Table 2).

[Table 2 here]

Within-Mothers Change in Depression (n=1760) and Anxiety (n=1634)

1,760 participants had both pre-Covid-19 and Covid-19 lockdown depression data and 1634 had pre-Covid-19 and Covid-19 lockdown anxiety data. Table 3 shows the change in depression and anxiety categorisations for these mothers across the two time points 2 (see also Supplementary Figure 1 & 2). The majority of mothers stayed in the same depression and anxiety categories across the two time points, for example 67% (n=759) who had no symptoms in the pre-Covid-19 data continued to have no symptoms during lockdown; and 54% (n=109) of those who had moderate/severe symptoms before the pandemic, remained in this category in the lockdown survey). However, many mothers' mental health worsened, with 38% (n=230) who had no/mild symptoms pre-Covid-19 reporting moderate/severe symptoms in the lockdown survey. A smaller number of mothers' mental health improved: of those mothers with moderate/severe symptoms pre-Covid-19, 24% (n=48) subsequently reported no symptoms and 23% (n=46) reported mild depression in the Covid-19 survey. Similar patterns of change were seen with the anxiety categories.

Factors associated with a clinically important increase in Depression (n=1760) and Anxiety (n=1634)

367 (21%) of mothers reported a clinically important increase (5 or more points) in depressive symptoms, and 348 (21%) reported a clinically important increase (5 or more points) in anxiety symptoms.

Table 4 presents the odds ratios for a clinically important increase in PHQ-8 and GAD-7 scores in relation to each exposure variable from the unadjusted logistic regression models. The estimates resulting from these models are imprecise, with wide confidence intervals.

Financial, food and housing insecurity during lockdown were associated with a higher likelihood of a clinically important increase in both depression and anxiety symptoms: the odds were more than 6 times greater for women who were financially insecure (Odds Ratio (OR) 6.23 [3.96-9.8] for depression; OR 6.03 [3.82-9.51] for anxiety) and over 3 times greater in mothers who were food insecure (OR 3.33 [2.09-5.28] for depression; OR 3.46 [2.15-5.58] for anxiety) or housing insecure (OR 3.29 [2.36-4.58] for depression; OR 3.0 [2.11-4.25] for anxiety).

Social circumstances were also associated with an increase in depression and anxiety: The odds of increased depression or anxiety were more than eight times greater in mothers reporting loneliness (OR 8.37 [5.7-12.27] for depression; OR 8.5 [5.71-12.65] for anxiety), and a lack of social support doubled the likelihood of an increase in depression and anxiety (OR 2.25 [1.78-2.86]; OR 2.13 [1.67-2.73] respectively). An average / poor relationship increased the odds of experiencing symptoms of depression by 3.6 [2.44-

For peer review only - http://bmjopen.bmj.com/site/about/guidelines.xhtml

5.43] and of anxiety by 5.1 [3.37-7.62]. A larger household size reduced the OR for depressive symptoms (OR 0.73 [0.53-0.99]), but not for anxiety (OR 0.90 [0.63-1.29].

A lack of physical activity during lockdown was associated with an increased OR for both depression and anxiety (OR 3.13 [2.15-4.56]; OR 2.55 [1.72-3.78], respectively). Mothers who did the same amount of physical activity during lockdown as they had done pre-Covid-19 reduced the odds of an increase in depressive symptoms by 34% (OR 0.66 [0.45-0.97]).

[Table 4 here]

There was no clear association between ethnicity (Pakistani heritage compared to White British) and an increase in depression (OR 0.88 [0.68-1.15]) or anxiety (OR 0.94: [0.72-1.23]). When the unadjusted regression analysis was repeated separately for White British and Pakistani heritage mothers, interesting differences in the magnitude of the association between exposure variables and a clinically important increase in symptoms of depression and anxiety was found, although confidence intervals are wide (Table 5a and 5b). The odds of an increase in depression and anxiety were greater for Pakistani heritage women who reported loneliness (OR 11.22 [6.45-19.53] for depression and OR 11.27 [6.16-20.56] for anxiety) compared to White British mothers (OR 7.17 [3.66-14.01] and OR 5.90 [3.03-11.47] respectively). There was also a greater magnitude of association for increased depression, but not anxiety, for Pakistani heritage women who reported an average / poor relationship with their partner (OR 4.91 [2.78-8.67] for depression and OR 4.99 [2.72-9.14] for anxiety), compared to White British mothers (OR 2.61 [1.31-5.20] and OR 4.39 [2.26-8.53] respectively).

In contrast the magnitude of the odds of an increase in depression and anxiety was greater for White British women who reported financial insecurity (OR 12.14 [5.15-28.60] for depression and OR 7.69 [3.43-17.26] for anxiety) or never doing physical activity (OR 5.54 [2.69-11.43] and OR 3.90 [1.82-8.36] respectively) compared to Pakistani heritage women (financial insecurity: OR 5.79 [2.90-11.56] for depression and OR 4.35 [2.27-8.30] for anxiety); (physical activity: OR 3.34 [1.88-5.92] and OR 2.50 [1.40-4.45] respectively).

For Pakistani heritage mothers living in large households, the odds of an increase in depression was reduced (OR 0.54 [0.31-0.94]) compared to White British mothers for whom there was no association (OR 0.91 [0.47-1.73]. There were no clear associations between ethnicity and household size for anxiety) (OR 0.61 [0.34-1.07] and OR 1.13 [0.58-2.20]).

[Table 5 here]

Free text responses to the question "what are your three biggest worries at the moment" were available for 1799 mothers. Only a small proportion of women identified their mental health issues as one of their biggest worries, n=105 (6%, 95%CI: 5%-7%), slightly greater in White British mothers, n=51 (8%, 95%CI: 6%-11%) than in mothers of Pakistani heritage, n=32 (4%, 95%CI: 3%-5%). More often, mothers reported how wider issues and concerns impacted on their mental health and wellbeing:

Health anxieties about Covid-19: the most commonly reported worry was a fear of bringing the virus home (e.g. from the shops or from their places of work), and themselves or members of their family becoming ill or dying, as well as the fear of what would happen to their children if this did happen to them.

I worry about contracting coronavirus particularly whilst at work and either becoming critically myself unwell or bringing it home to my family

Feeling anxious about the virus and constantly worrying about my kids which 2 of them have health issues and are quite vulnerable

I feel particularly anxious to even step out of the house even for food shopping or taking a walk/exercise

I worry how this will affect my children. I'm terrified they will be separated as I have 2 children with my ex-husband and one with my current. So I haven't been outside in 10 weeks.

For peer review only - http://bmjopen.bmj.com/site/about/guidelines.xhtml

Mental load: mothers often reported the mental load of managing work, home-schooling, childcare and domestic tasks, without the break provided by children attending school, nursery or other childcare. Being or feeling stuck inside and unable to move around freely contributed to a sense of suffocation and feeling overwhelmed, and many mothers acknowledged that this was having a detrimental effect on their mental health and self-esteem:

I'm worried [about] having a nervous breakdown or a panic attack...can't get a break from all the responsibilities and go somewhere for fresh air even

Finding working from home and looking after children very demanding. No time alone. No silence. Surrounded by people and electronics all my waking hours.

I am a keyworker who works five days of week, only while my children are at school. I am becoming mentally drained/exhausted constantly being on duty, it is impacting my mental health.

Homeschooling I am not cut out for this and don't feel good enough

Loss of social support: The loss of social support caused by lockdown, especially for those who didn't live with their partner or were single parents was highlighted as causing loneliness and isolation for some.

My support network was my partner, who I cannot see due to lockdown.

Not able to see my boyfriend - feel isolated and alone once children are in bed with no adult face to face interaction

The isolation and loneliness has been a challenge and having to take care of my child on my own full-time

Being a single parent of a disabled child I rely on my social life/friends for my mental wellbeing and I worry about when it'll be safe to see/hug my immediate family who live locally

Financial and employment insecurity: Household finances and the stress of unemployment or job insecurity related to lockdown measures were also a major worry for women. Many families were in debt, reliant on credit cards and benefits, and in insecure employment before the lockdown measures began and were only just about getting by prior to the pandemic.

I have struggled financially during this time. I would like to not worry about money and bills and shopping and outgoings. I have enough worries being a full time carer.

Worried about the financial impact of covid 19. I am currently furloughed from work but I worry that the virus will have an impact on the business. My husband is self-employed and is not eligible to any funds.

Being unable to switch off: Participants described being frightened of the news reports but unable to switch off, and were wondering when, if ever, things would become normal again:

Worry about my mental health, I know I'm struggling. Worry about the future and how this will affect the country financially. Worry about my children's education & what they've missed out on.

All the bad news on the TV, and the death rate on the News. All the information on the news makes me panic more.

When will this end? Will it end?

A loss of coping strategies: For those who had existing mental health issues before lockdown, the lockdown measures had often taken away their sources of support, their normal routines and methods of coping. In addition, some respondents reported being unable to access mental health services due to Covid-19 and lockdown measures:

Mental health - Exercise at the gym was a coping mechanism, now closed. Can do bits at home but nothing like at the gym. Cannot get any gym equipment - out of stock.

Mental Health - struggling to motivate myself and to keep to a routine. Self-destructive behaviour - drink drugs and binge eating. I am missing my work and colleagues.

For peer review only - http://bmjopen.bmj.com/site/about/guidelines.xhtml

Not getting my mental health support since the lockdown. My CPN (Community Psychiatric Nurse) not returning my calls. It has made me a lot worse. I try to talk to my husband so I am not keeping everything inside

Mental health, I have had previous issues in the past and am struggling and don't feel like I can approach my GP at the minute as it isn't an emergency

Positive aspects of lockdown: Many participants reported positive aspects to changes enforced by the lockdown, commenting that they were getting to spend more quality time with their children, were enjoying a slower pace of life, a more relaxed routine and spending less time driving and commuting.

Life has become a lot more relaxed over the last 3 weeks, no manic mornings trying to get everybody out of the house, time with kids, doing stuff with kids I would normally say 'not now' to. Get to know kids more. More time outside, [doing] jobs in the house that need doing.

Spending more time with my husband and feeling appreciative of each other and having a relaxed day with the children instead of running to school to mosque and then all the extra clubs it's a more relaxed day.

Ramadan is the easiest it has ever been, we are free to make up our sleep and not push ourselves too much, had time to do nice things during Ramadan including having a more peaceful time not having to do school runs, be stressed out, my husband had a chance to take a slower pace to life and not get too stressed.

DISCUSSION

We compared depression and anxiety during the March-June 2020 UK lockdown to pre-Covid-19 depression and anxiety data collected in our longitudinal birth cohort studies. We found that clinically important symptoms of depression and anxiety increased from 11% to 20% and from 11% to 16%, respectively. These findings reflect those of other longitudinal studies which have reported similar changes in mental health from before to during the Covid-19 pandemic[3,4,5].

We hypothesised that our vulnerable population with diverse ethnicity and high levels of deprivation would be susceptible to increases in depression and anxiety during the lockdown. Financial, food and housing insecurity all increased the odds of an increase in depression and anxiety, as did loneliness, a lack of social support, an average/poor partner relationship and a lack of physical activity.

There was no clear association between White British and Pakistani heritage mothers and a clinically important increase in depression or anxiety. However, when we separated out the regression analyses by ethnicity, we found interesting differences in the magnitude of the associations with an increase in depression and anxiety: Mothers from a Pakistani heritage had greater odds of an increase in depression and anxiety if they were lonely or had an average/poor relationship (for depression, but not anxiety) than White British mothers. Pakistani heritage mothers had a much reduced odds of an increase in depression if they lived in a large household compared to White British mothers (for whom there was no association). In contrast, mothers of White British ethnicity had greater odds of an increase if they were financially insecure and/or physically inactive compared to Pakistani heritage mothers reporting the same exposures.

The free text responses supported these findings, highlighting a complex inter-play of challenges contributing to poor mental health in mothers including: acute health anxieties; the mental load of managing multiple roles and responsibilities; the loss of social support and other coping strategies; pressures of financial and employment insecurity; and being unable to switch off from the pandemic.

The potential ethnic differences in the magnitude of the associations of different variables and increased mental ill health reported in this study warrant further investigation, including an understanding of potentially differing protective factors in different ethnic groups. We have previously reported, outside of the Covid-19 context, that (a) White British mothers are more likely to have their mental ill health identified by health professionals than South Asian mothers , and that (b) both White British and South Asian mothers are equally likely to disclose symptoms in self-report research questionnaires, as used here.[23,24] Ethnic differences in household structure, and cultural practices might provide more support in times of adversity, as we have found in the case of food insecurity previously[25,26].

For peer review only - http://bmjopen.bmj.com/site/about/guidelines.xhtml

This is a longitudinal study comparing data collected before the Covid-19 pandemic and during the March-June 2020 lockdown which has allowed us to explore change over that time period. It also provides findings from a highly ethnically diverse population, the majority of whom live in the most deprived centiles in the UK. We are not aware of other studies that have explored longitudinal change in mental health from pre- to during- the Covid-19 lockdown in a similar population.

Respondents were mothers of children aged 0-5 and/or 9-13 which may limit the wider generalizability, though our findings are broadly similar to those from a previous longitudinal study of two UK cohorts that included adult men and women (not all of whom were mothers), and found the increased risk of poor mental health in lockdown to be greater in women[4]. Our pre-Covid-19 measures were taken from data collected over the past 4 years, so we cannot, with confidence, attribute all changes to the pandemic and the lockdown. For example, it is possible that some of the difference reflects age related change in the women and/or their children over time. It is also possible that we have underestimated some of the adverse impact of lockdown as a significant percentage of the BiBBS participants were pregnant at baseline, which itself is associated with raised levels of mental ill health. However our analysis did not find increased levels of depression and anxiety for the BiBBS cohort compared to the BiBGU cohort. Similarly, our analyses did not find any association between time since baseline data capture and the odds of an increase in depression and anxiety, suggesting that the timing of our baseline collection is not influencing our findings...

We undertook unadjusted logistic regression analyses to explore possible factors that might explain or influence changes in mental health from before- to during- the pandemic, and repeated these separating out the two main ethnic groups. This approach avoids the difficulties inherent in interpreting the ethnicity coefficient in regression models controlling for other variables[20], however it also limits the interpretation of the data. In addition, the total sample size for this mental health analysis was ~1,700 mothers and many estimates in the analyses were, as a consequence, imprecise with wide confidence intervals. These results demonstrate the need for further studies with sufficient sample sizes, including boosted samples of ethnic minority groups to enable accurate understanding of the possible differing experiences and needs of the diverse UK population.

Whilst it is possible that our results are influenced by the survey response rate of 28%, these participants were representative of the BiBGU and BiBBS cohorts[15], including on the rates of baseline depression and anxiety scores, and have demonstrated a wide variability in most characteristics (Table 1).

We undertook stratified analyses to explore ethnic differences in how exposures related to changes in mental health between pre-pandemic times to during lockdown. However, whilst our analysis was able to look for differences between Pakistani heritage and White British mothers, we were unable to explore the heterogeneity of the 'other' ethnic group and we have no doubt missed important nuances in this population based on different social and cultural experiences.

We will continue to follow the BiBGU and BiBBS families so that we can look at trajectories of change over time, including during any future national or regional lockdowns. At time of writing (November 2020), the UK was under a further nationwide lockdown, following a period of reduced restrictions during summer 2020 and tighter regional restrictions in the more deprived cities of Northern England, including Bradford, though the autumn. The impact of further and longer periods of restrictions and lockdowns will be a focus of our study moving forwards. As the longitudinal follow-up lengthens and more data become available it may be possible to model different transitions in mental health in relation to changing social and economic circumstances. It may also be of interest to explore positive / null transitions from clinically important symptoms to mild/no symptoms, or continued mild/no symptoms despite reported exposure to adversity.

Our results highlight the potential public health impact of lockdown on mental health, particularly in those who are lonely and economically insecure. Mental health problems are in general less visible than physical symptoms and in particular the physical symptoms related to Covid-19, including the acutely ill patients in ICUs, but may have more significant longer-term consequences. This study also suggests a complex interplay of factors between an individuals' circumstances and the odds of worsening mental health during the pandemic. A 'one-size fits all' approach to supporting mental ill health will not be effective; instead, understanding and addressing the potential causes in different groups will be important. For example, in case of future need for social distancing measures, government and local councils should consider policies that permit 'social bubbles' that can be implemented to reduce loneliness for those at risk of mental health problems, and they as well as voluntary services should continue to focus support to those who are lonely/isolated. Policy and decision makers should also make provision for the continuing need to support

For peer review only - http://bmjopen.bmj.com/site/about/guidelines.xhtml

and protect vulnerable families from financial, food and housing insecurity, all of which were associated with poor mental health in this study. These actions will be important throughout any further regional and national lockdowns and during the post-pandemic period of recovery.

For peer review only

For peer review only - http://bmjopen.bmj.com/site/about/guidelines.xhtml

DISSEMINATION DECLARATION

The findings of the study were also shared with our community research advisory groups to enhance interpretation. We will share our findings with all participants using a combination of social media, infographics posted to participants and community dissemination events

DATA (AND SOFTWARE) AVAILABILITY

Born in Bradford offer open access to their data resources. Available data and procedures to access this can be found at: https://borninbradford.nhs.uk/research/how-to-access-data/_

AUTHOR CONTRIBUTIONS

JD was involved in the concept and study design, design of the data collection tools, overall supervision of the study and data collection, wrote the statistical analysis plan, and drafted and revised the paper. She is guarantor. BK was involved in the design of data collection tools, wrote the statistical analysis plan, conducted all statistical analyses, and drafted and revised the paper. BL devised the coding framework for free text responses, analysed the data and drafted and revised the paper. SB was involved in the concept and study design, design of the data collection tools, supervision of the study and data collection, and reviewing and revising the paper. CC was involved in the concept and study design, and reviewing and revising the paper. KW was involved in design of the data collection tools, data curation, analysis, reviewing and revising the paper. KS was involved in the study design, design of the data collection tools, supervision of the study and data collection, and reviewing and revising the paper. KC was involved in the study design, design of the data collection tools, supervision of the study and data collection, data curation and reviewing and revising the paper. MB involved in the study design, and drafted and revised the paper. NS advised on the clinical significance of changes in mental ill health, revising the statistical analysis plan, and drafted and revised the paper. TS was involved in the concept and study design, supervision and support to junior members of the team and drafted and revised the paper. DAL was involved in the concept and study design, design of the data collection tools, and drafted and revised the paper. JW was involved in the concept and study design, design of the data collection tools, supervision and support to junior members of the team and drafted and revised the paper. RM was involved in the concept and study design, design of the data collection tools, revised the statistical analysis plan, and drafted and revised the paper. KP was involved in the concept and study design, design of the data collection tools, revised the statistical analysis plan, supervision and support to junior members of the team and drafted and revised the paper.

COMPETING INTERESTS

All authors have completed the ICMJE uniform disclosure form at www.icmje.org/coi_disclosure.pdf and declare: all authors had financial support from funding bodies listed below for the submitted work; no financial relationships with any organisations that might have an interest in the submitted work in the previous three years; no other relationships or activities that could appear to have influenced the submitted work, with the exception of DAL who declares previous support from Roche Diagnostics and Medtronic Ltd for research unrelated to that presented here.

FUNDING INFORMATION

This study was funded by The Health foundation Covid-19 Award (2301201), with further contributions from: A Wellcome Trust infrastructure grant (WT101597MA); A joint grant from the UK Medical Research Council (MRC) and UK Economic and Social Science Research Council (ESRC): MR/N024391/1; The National Institute for Health Research under its Applied Research Collaboration Yorkshire and Humber (NIHR200166); ActEarly UK Prevention Research Partnership Consortium (MR/S037527/1); Better Start Bradford through The National Lottery Community Fund; and the British Heart Foundation (CS/16/4/32482). DAL works in a unit that received support from the University of Bristol and UK Medical Research Council (MC_UU_00011/6) and is a UK National Institute of Health Research senior Investigator (NF-0616-10102)

For peer review only - http://bmjopen.bmj.com/site/about/guidelines.xhtml

ACKNOWLEDGEMENTS

Born in Bradford is only possible because of the enthusiasm and commitment of the children and parents in BiB. We are grateful to all the participants, parent governors and community research advisory group members, schools, health professionals and researchers who have made Born in Bradford happen.

This report is independent research partly funded by the National Institute for Health Research Yorkshire and Humber ARC. The views expressed in this publication are those of the authors and not necessarily those of the National Institute for Health Research or the Department of Health and Social Care.

TRANSPARENCY STATEMENT

The lead author (the manuscript's guarantor) affirms that the manuscript is an honest, accurate, and transparent account of the study being reported; that no important aspects of the study have been omitted; and that any discrepancies from the study as originally planned (and, if relevant, registered) have been explained.

For peer review only

For peer review only - http://bmjopen.bmj.com/site/about/guidelines.xhtml

1. REFERENCES

1. Coronavirus (Covid-19) UK Government Response. https://www.gov.uk/coronavirus. Accessed 27.11.20

2. Number of coronavirus (COVID-19) cases and risk in the UK. UK Government Guidance. https://www.gov.uk/guidance/coronavirus-covid-19-information-for-the-public . Accessed 27.11.20

3. Office of National Statistics (2020). Coronavirus and depression in adults, Great Britain: June 2020. https://www.ons.gov.uk/peoplepopulationandcommunity/wellbeing/articles/coronavirusanddepressioninadultsgreatbritain/june2020. Accessed 27.11.20

4. Kwong ASF, Pearson RM, Adams MJ, et al., Mental health during the COVID-19 pandemic in two longitudinal UK population cohorts. *MedRxiv* 2020; 20133116. doi: 10.1101/2020.06.16.20133116

5. Pierce M, Hope H, Ford T, et al., Mental health before and during the COVID-19 pandemic: a longitudinal probability sample survey of the UK population. *Lancet Psychiatry* 2020, https://doi.org/10.1016/S2215-0366(20)30308-4

6. Mind (2020). The mental health emergency: how has the coronavirus pandemic impacted our mental health? London: Mind. Available at: mind.org.uk. Accessed 27.11.20

7. Fancourt D, Steptoe A, Bu F. Trajectories of depression and anxiety during enforced isolation due to COVID-19: longitudinal analyses of 59,318 adults in the UK with and without diagnosed mental illness. medRxiv 2020. **doi:** https://doi.org/10.1101/2020.06.03.20120923

8. Bignall T, Jeraj S, Helsby E, Butt J. Racial disparities in mental health: Literature and evidence review. Race Equality Foundation 2019. https://raceequalityfoundation.org.uk/wp-content/uploads/2020/03/mental-health-report-v5-2.pdf. Accessed 27.11.20

9. Grey T, Sewell H, Shapiro G, Ashraf F. Mental Health Inequalities Facing U.K. Minority Ethnic Populations Causal Factors and Solutions. J Psychological Issues in Organizational Culture. 2013: 3 (S1).

10. Public Health England. COVID-19: mental health and wellbeing surveillance report: https://www.gov.uk/government/publications/covid-19-mental-health-and-wellbeing-surveillance-report/2-important-findings-so-far#which-population-groups-appear-to-be-disproportionally-affected. Accessed 27.11.20

11. Wright J, Raynor P, Tuffnell D, et al. Cohort Profile: The Born in Bradford multi-ethnic family cohort study. *International Journal of Epidemiology*, Volume 42, Issue 4, August 2013, Pages 978–991, https://doi.org/10.1093/ije/dys112

12. Bird PK, McEachan RRC, Mon-Williams M, Small N, West J, Whincup P, Wright J, Andrews E, Barber SE, Hill LJB *et al*: Growing up in Bradford: protocol for the age 7–11 follow up of the Born in Bradford birth cohort. *BMC Public Health* 2019, 19(1):939.

13. Dickerson J, Bird PK, McEachan RRC, Pickett KE, Waiblinger D, Uphoff E, Mason D, Bryant M, Bywater T, Bowyer-Crane C *et al*: Born in Bradford's Better Start: an experimental birth cohort study to evaluate the impact of early life interventions. *BMC Public Health* 2016, 16(1):711.

14. McEachan RRC, Dickerson J, Bridges S *et al.* The Born in Bradford COVID-19 Research Study: Protocol for an adaptive mixed methods research study to gather actionable intelligence on the impact of COVID-19 on health inequalities amongst families living in Bradford [version 1; peer review: 3 approved]. *Wellcome Open Res* 2020, **5**:191 (https://doi.org/10.12688/wellcomeopenres.16129.1)

15. Dickerson J, Kelly B, Lockyer B *et al.* Experiences of lockdown during the Covid-19 pandemic: descriptive findings from a survey of families in the Born in Bradford study [version 2; peer review: 2 approved]. *Wellcome Open Res* 2021, **5**:228 (https://doi.org/10.12688/wellcomeopenres.16317.2)

16. Kroenke K, Strine TW, Spitzer RL, Williams JBW, Berry JT, Mokdad AH: The PHQ-8 as a measure of current depression in the general population. *Journal of Affective Disorders* 2009, 114(1):163-173.

For peer review only - http://bmjopen.bmj.com/site/about/guidelines.xhtml

17. Löwe B, Decker O, Müller S, Brähler E, Schellberg D, Herzog W, Herzberg PY: Validation and Standardization of the Generalized Anxiety Disorder Screener (GAD-7) in the General Population. *Medical Care* 2008, 46(3):266-274.

18. Husain N, Waheed W, Tomenson B, Creed F. The validation of personal health questionnaire amongst people of Pakistani family origin living in the United Kingdom. Journal of Affective Disorders 2007, 97: 261-264.

19. McMillan D, Gilbody S, Richards D. Defining successful treatment outcome in depression using the PHQ-9: A comparison of methods. J. Affective Disorders. 2010: 127; 122–129

20. VanderWeele, T. J., & Robinson, W. R. On the causal interpretation of race in regressions adjusting for confounding and mediating variables. *Epidemiology.* 2014: *25*(4), 473–484.

21. StataCorp. 2017. Stata Statistical Software: Release 15. College Station, TX: StataCorp LLC.

22. Braun V, Clarke V: Using thematic analysis in psychology. *Qualitative Research in Psychology* 2006, 3(2):77-101.

23. Prady SL, Pickett KE, Petherick ES, Gilbody S, Croudace T, Mason D, Sheldon TA, Wright J. (2016). Evaluation of ethnic disparities in detection of depression and anxiety in primary care during the maternal period: combined analysis of routine and cohort data. The British journal of psychiatry, 208(5), 453-61. DOI 10.1192/bjp.bp.114.158832. PMID 26795424

24. Prady SL, Pickett KE, Gilbody S, Petherick ES, Mason D, Sheldon TA, and Wright J (2016). Variation and ethnic inequalities in treatment of common mental disorders before, during and after pregnancy: combined analysis of routine and research data in the Born in Bradford cohort. BMC Psychiatry, 16(99), DOI 10.1186/s12888-016-0805-x PMID 27071711

25. Power M, Uphoff N, Stewart-Knox B et al., Food insecurity and socio-demographic characteristics in two UK ethnic groups: an analysis of women in the Born in Bradford cohort. *Journal of Public Health*, 2017. **40**(1): p. 32-40.

26. Yang T.C, Sahota P, Pickett K, Bryant M. Association of food security status with overweight and dietary intake: exploration of White British and Pakistani-origin families in the Born in Bradford cohort. *Nutrition Journal*, 2018. **17**(1): p. 48.

For peer review only - http://bmjopen.bmj.com/site/about/guidelines.xhtml

Table 1: Sample Characteristics of Mothers who responded to the BiB Covid-19 survey (n=1860)

**Financial security**		
Living comfortably	354	20% (18%-22%)
Doing alright	766	42% (40%-45%)
Just about getting by	462	26% (24%-28%)
Finding it quite/ very difficult	223	12% (11%-14%)
Missing	55	
**Change in financial status (compared to 3 months prior, as asked in lockdown survey)**		
Better off	166	10% (8%-11%)
About the same	989	58% (55%-60%)
Worse off	562	33% (31%-35%)
Missing	143	
**Index of Multiple Deprivation 2019 Quintile**		
1: Most Deprived	1,211	65% (63%-67%)
2	390	21% (19%-23%)
3	139	7% (6%-9%)
4	88	5% (4%-6%)
5: Least Deprived	29	2% (1%-2%)
Missing	3	
**Employment status of main earner**		
Employed - working from home	393	22% (20%-24%)
Employed: going into work	567	32% (30%-34%)
On furlough	271	15% (14%-17%)
Self-employed: working	147	8% (7%-10%)
Self-employed: not working	200	11% (10%-13%)
Unemployed	208	12% (10%-13%)
Missing	74	
**Whether respondent is a keyworker**		
No	1,259	68% (66%-71%)
Yes	579	32% (29%-34%)
Missing	22	
**Whether anyone in household is clinically vulnerable to Covid-19**		
No	1,419	77% (75%-79%)
Yes	426	23% (21%-25%)
Missing	15	
**Whether anyone in household is currently/ has previously self-isolated**		
No	1,331	72% (70%-74%)
Yes	508	28% (26%-30%)
Missing	21	
**Number of children in household**		
One	387	21% (19%-23%)
Two	699	38% (35%-40%)
Three	451	24% (22%-26%)
Four or more	323	17% (16%-19%)
**Total household size**		

For peer review only

Two/ Three	321	17% (16%-19%)
Four/ Five	943	51% (49%-53%)
Six or more	583	32% (29%-34%)
Missing	13	
**Whether single parent**		
No	1,604	88% (86%-89%)
Yes	222	12% (11%-14%)
Missing	34	
**Quality of relationship with partner (for those married or in a relationship, n=1604)**		
Excellent	783	51% (48%-53%)
Good	598	39% (36%-41%)
Average	129	8% (7%-10%)
Poor	23	1% (1%-2%)
Very poor	10	1% (0%-1%)
Missing	61	
**Loneliness: How often felt lonely in the past week**		
None, or almost none of the time	998	57% (55%-60%)
Some of the time	555	32% (30%-34%)
Most of the time	131	8% (6%-9%)
All, or almost all of the time	56	3% (2%-4%)
Missing	120	
**Social support: how easy to get help from friends/ neighbours if needed**		
Very difficult	101	6% (5%-7%)
Difficult	146	8% (7%-9%)
Possible	529	29% (27%-31%)
Easy	529	29% (27%-31%)
Very easy	513	28% (26%-30%)
Missing	42	
**Food security: How often is it true that food does not last**		
Never	1,379	79% (77%-81%)
Sometimes	279	16% (14%-18%)
Often	91	5% (4%-6%)
Missing	111	
**Housing security: Whether worried about eviction/ repossession**		
Strongly disagree or disagree	1,250	71% (68%-73%)
Neither	328	19% (17%-20%)
Strongly agree/ agree	194	11% (10%-12%)
Missing	88	
**Job security: Whether they expect the main earner to still have a job in 12 months (for those currently employed (n=1652))**		
No	77	5% (4%-6%)
Yes	979	61% (59%-64%)
Don't know	537	34% (31%-36%)
Missing	59	
**Housing conditions: Whether damp in house**		
No	1,307	71% (69%-73%)
Yes	534	29% (27%-31%)
Missing	19	

For peer review only

**Frequency of physical activity currently**		
Never	239	13% (12%-15%)
1 or 2 days a week	489	27% (25%-29%)
Most days	516	28% (26%-30%)
Every day	586	32% (30%-34%)
Missing	30	
**Change in frequency of physical activity since lockdown**		
Less	766	48% (46%-51%)
About the same	408	26% (24%-28%)
More	419	26% (24%-29%)
Missing	267	

For peer review only

Table 2: Depression and Anxiety Scores in the Pre-Covid-19 and Covid-19 surveys (n=1860).

	Pre-Covid-19 survey		Covid-19 survey	
	N	Percentage (95% CI)	N	Percentage (95% CI)
**Depression category (PHQ-8)**				
None (PHQ-8 score 0 to 4)	1187	65% (63%-68%)	1001	56% (54%-59%)
Mild (PHQ-8 score 5 to 9)	414	23% (21%-25%)	425	24% (22%-26%)
Moderate to Severe (PHQ-8 score 10 to 24)	212	12% (10%-13%)	349	20% (18%-22%)
Missing	47		85	
**Anxiety category (GAD7)**				
None (GAD-7 score 0 to 4)	1280	75% (72%-77%)	1075	61% (58%-63%)
Mild (GAD-7 score 5 to 9)	270	16% (14%-18%)	408	23% (21%-25%)
Moderate to Severe (GAD-7 score 10 to 21)	167	10% (8%-11%)	289	16% (15%-18%)
Missing	143		88	

For peer review only

Table 3: Change in PHQ-8 and GAD-7 categories from Pre-Covid-19 baseline to Covid-19 lockdown survey.

Pre-Covid-19 PHQ8 Category	PHQ8 at Covid-19 Lockdown Survey None	Mild	Moderate/ Severe	Missing	Total
None	759 (67%)	257 (23%)	118 (10%)	53	1187 (100%)
Mild	171 (44%)	110 (28%)	112 (28%)	21	414 (100%)
Moderate/ Severe	48 (24%)	46 (23%)	109 (54%)	9	212 (100%)
Missing	23	12	10	2	47 (100%)
Total	978	413	339	85	1860 (100%)

Pre-Covid-19 GAD7 Category	GAD7 at Covid-19 Lockdown Survey None	Mild	Moderate/ Severe	Missing	Total
None	851 (70%)	247 (20%)	118 (10%)	64	1280 (100%)
Mild	95 (37%)	91 (36%)	70 (27%)	14	270 (100%)
Moderate/ Severe	47 (29%)	44 (27%)	71 (44%)	5	167 (100%)
Missing	82	26	30	5	143 (100%)
Total	993	382	259	88	1860 (100%)

RED: category worse in lockdown compared to baseline;
AMBER: category stayed the same;
GREEN: category improved in lockdown compared to baseline

For peer review only

Table 4: Odds ratios (with 95% CI) from unadjusted logistic regression models for an increase of 5 points or more on PHQ-8 and GAD-7 between pre-Covid-19 and Covid-19 lockdown survey.

	PHQ-8 increase >= 5			GAD-7 increase >= 5		
**Exposure**	**Odds Ratio**	**Low 95% CI**	**High 95% CI**	**Odds Ratio**	**Low 95% CI**	**High 95% CI**
**Ethnicity (Reference: White British)**						
Pakistani heritage	0.88	0.68	1.15	0.94	0.72	1.23
**Age group (Reference: Under 30 years)**						
30 to 34 years	1.01	0.66	1.54	0.90	0.57	1.42
35 to 39 years	0.89	0.59	1.35	0.99	0.64	1.53
40 to 44 years	1.04	0.69	1.58	0.88	0.57	1.38
45 years plus	0.92	0.59	1.45	1.02	0.64	1.63
**Financial security at pre-Covid-19 survey (Reference: Living comfortably)**						
Doing alright	1.07	0.81	1.42	1.19	0.89	1.59
Just about getting by	1.72	1.23	2.40	1.66	1.17	2.35
Finding it quite/ very difficult	1.70	1.03	2.80	2.39	1.44	3.98
**Financial security at lockdown survey (Reference: Living comfortably)**						
Doing alright	1.74	1.18	2.56	1.47	1.00	2.17
Just about getting by	3.52	2.36	5.26	3.20	2.15	4.76
Finding it quite/ very difficult	6.23	3.96	9.80	6.03	3.82	9.51
**Change in financial status compared to 3 months ago (Reference: Better off)**						
About the same	0.65	0.43	0.98	0.88	0.55	1.40
Worse off	1.36	0.89	2.01	1.99	1.25	3.19
**Index of Multiple Deprivation 2019 Quintile (Reference: 1 Most deprived fifth)**						
2	0.90	0.67	1.21	1.22	0.92	1.63
3	0.91	0.58	1.41	0.87	0.55	1.39
4	0.76	0.43	1.34	0.98	0.57	1.68
5 Least deprived fifth	0.85	0.34	2.13	0.99	0.39	2.48
Per 1 fifth increase in lower deprivation	1.07	0.94	1.25	1.00	0.88	1.13
**Employment status of main earner (Reference: Employed and working from home)**						
Employed: going into work	1.18	0.84	1.65	1.20	0.85	1.69
On furlough	1.19	0.79	1.79	1.29	0.85	1.94
Self-employed: working	0.90	0.53	1.51	0.92	0.55	1.57
Self-employed: not working	1.49	0.97	2.27	1.31	0.84	2.04
Unemployed	2.03	1.34	3.09	2.23	1.44	3.44
**Whether respondent is a keyworker (Reference: No)**						
Yes	0.99	0.77	1.27	1.05	0.82	1.35

For peer review only

**Whether anyone in household is clinically vulnerable to Covid-19 (Reference: No)**						
Yes	1.68	1.29	2.19	1.53	1.17	2.01
**Whether anyone in household is currently/ has previously self-isolated (Reference: No)**						
Yes	1.59	1.24	2.04	1.48	1.14	1.92
**Number of children in household (Reference: One)**						
Two	1.20	0.87	1.67	1.05	0.76	1.45
Three	1.39	0.98	1.98	1.20	0.84	1.71
Four or more	1.12	0.76	1.65	0.93	0.62	1.39
**Total household size (Reference: Less than four)**						
Four or Five	0.73	0.53	0.99	0.95	0.69	1.32
Six or more	0.72	0.51	1.00	0.90	0.63	1.29
**Whether single parent (Reference: No)**						
Yes	1.66	1.18	2.33	1.28	0.89	1.85
**Quality of relationship with partner (Reference: Excellent)**						
Good	1.93	1.45	2.57	1.82	1.35	2.44
Average to poor	3.64	2.44	5.43	5.07	3.37	7.62
**Loneliness (Reference: Not lonely)**						
Lonely most/ all of the time	8.37	5.70	12.27	8.50	5.71	12.65
**Social support (Reference: Easy to get support)**						
Not easy to get support	2.25	1.78	2.86	2.13	1.67	2.73
**Food security (Reference: Secure)**						
Insecure - Often true that food does not last	3.33	2.09	5.28	3.46	2.15	5.58
**Housing security (Reference: Secure)**						
Insecure - Worried about eviction/ repossession	3.29	2.36	4.58	3.00	2.11	4.25
**Job security: whether expect main earner to still have job in 12 months (Reference: Yes)**						
No	0.84	0.49	1.43	0.96	0.56	1.64
**Housing conditions (Reference: No damp in house)**						
Damp in house	1.85	1.45	2.38	1.57	1.21	2.03
**Frequency of physical activity (Reference: Every day)**						
Most days	1.58	1.15	2.18	1.67	1.21	2.31

For peer review only

1 or 2 days a week	1.93	1.40	2.67	1.67	1.20	2.31
Never	3.13	2.15	4.56	2.55	1.72	3.78
**Change in frequency of physical activity since lockdown (Reference: More than before)**						
About the same	0.66	0.45	0.97	0.82	0.56	1.21
Less than before	1.27	0.94	1.72	1.43	1.04	1.95
**Time between baseline and Covid-19 lockdown survey**						
Time (in Months)	1.00	0.99	1.01	1.00	0.99	1.01
**Cohort Source (Reference: BiBGU)**						
BiBBS	0.84	0.64	1.10	0.84	0.63	1.12

For peer review only

Table 5a: Odds ratios (with 95% CI) from unadjusted logistic regression models for an increase of 5 points or more on PHQ-8 between pre-Covid-19 and Covid-19 lockdown survey for White British and Pakistani heritage mothers.

	White British			Pakistani Heritage		
**Exposure**	**Odds Ratio**	**Low 95% CI**	**High 95% CI**	**Odds Ratio**	**Low 95% CI**	**High 95% CI**
**Financial security (Reference: Living comfortably)**						
Doing alright	2.00	1.12	3.56	1.70	0.88	3.27
Just about getting by	3.98	2.15	7.38	3.03	1.56	5.88
Finding it quite/ very difficult	12.14	5.15	28.60	5.79	2.90	11.56
**Food security (Reference: Secure)**						
Insecure	3.73	1.58	8.77	4.13	2.08	8.17
**Housing security (Reference: Secure)**						
Insecure	4.14	2.07	8.29	3.20	1.99	5.16
**Loneliness (Reference: not lonely)**						
Lonely most/ all of the time	7.17	3.66	14.01	11.22	6.45	19.53
**Social support (Reference: Easy to get support)**						
Not Easy	2.08	1.39	3.10	2.20	1.54	3.14
**Quality of relationship with partner (Reference: Excellent)**						
Good	1.68	1.02	2.77	1.76	1.15	2.68
Average to very poor	2.61	1.31	5.20	4.91	2.78	8.67
**Total household size (Reference: Less than four)**						
Four or Five	0.69	0.44	1.07	0.61	0.35	1.05
Six or more	0.91	0.47	1.73	0.54	0.31	0.94
**Frequency of physical activity (Reference: Every day)**						
Never	5.54	2.69	11.43	3.34	1.88	5.92
1 or 2 days a week	1.97	1.15	3.37	2.05	1.22	3.47
Most days a week	1.33	0.81	2.17	2.08	1.21	3.58

For peer review only

Table 5b: Odds ratios (with 95% CI) from unadjusted logistic regression models for an increase of 5 points or more on GAD-7 between pre-Covid-19 and Covid-19 lockdown survey for White British and Pakistani heritage mothers.

	White British			Pakistani Heritage		
**Exposure**	**Odds Ratio**	**Low 95% CI**	**High 95% CI**	**Odds Ratio**	**Low 95% CI**	**High 95% CI**
**Financial security (Reference: Living comfortably)**						
Doing alright	1.70	0.97	3.00	0.93	0.50	1.74
Just about getting by	2.82	1.53	5.21	2.55	1.39	4.68
Finding it quite/ very difficult	7.69	3.43	17.26	4.35	2.27	8.30
**Food security (Reference: Secure)**						
Insecure	3.59	1.47	8.7	4.96	2.49	9.87
**Housing security (Reference: Secure)**						
Insecure	3.32	1.67	6.57	3.07	1.84	5.10
**Loneliness (Reference: not lonely)**						
Lonely	5.90	3.03	11.47	11.27	6.16	20.56
**Social support (Reference: Easy to get support)**						
Not Easy	2.33	1.55	3.51	2.09	1.45	3.01
**Quality of relationship with partner (Reference: Excellent)**						
Good	1.54	0.93	2.55	1.88	1.22	2.89
Average to very poor	4.39	2.26	8.53	4.99	2.72	9.14
**Total household size (Reference: Less than four)**						
Four or Five	1.01	0.63	1.62	0.64	0.36	1.14
Six or more	1.13	0.58	2.20	0.61	0.34	1.07
**Frequency of physical activity (Reference: Every day)**						
Never	3.90	1.82	8.36	2.50	1.40	4.45
1 or 2 days a week	1.52	0.88	2.63	1.61	0.97	2.70
Most days a week	1.45	0.90	2.34	1.86	1.09	3.18

For peer review only

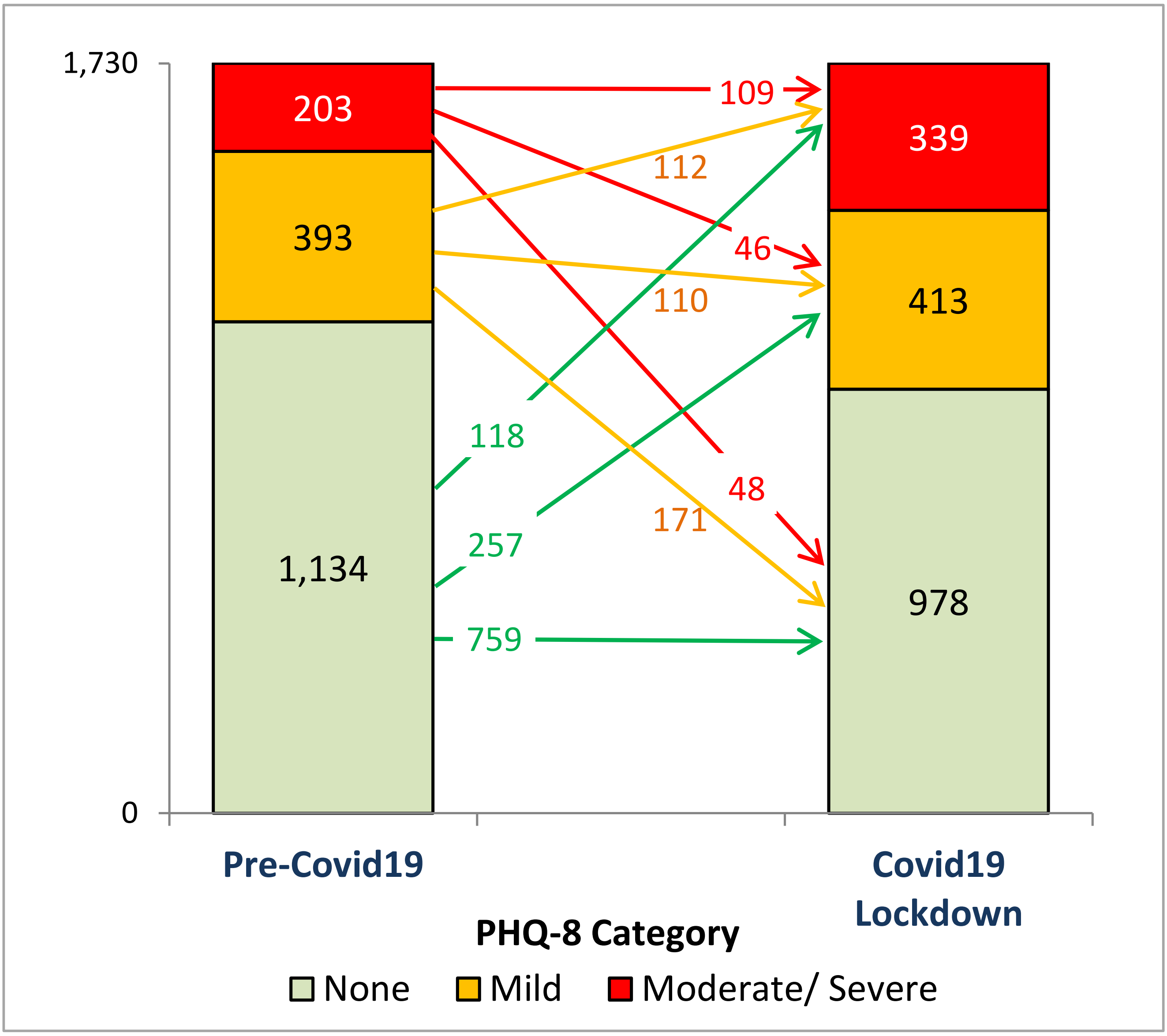
1,730
0
203
393
1,134
339
413
978
109
112
46
110
118
48
171
257
759
Pre-Covid19
Covid19 Lockdown
PHQ-8 Category
None
Mild
Moderate/ Severe

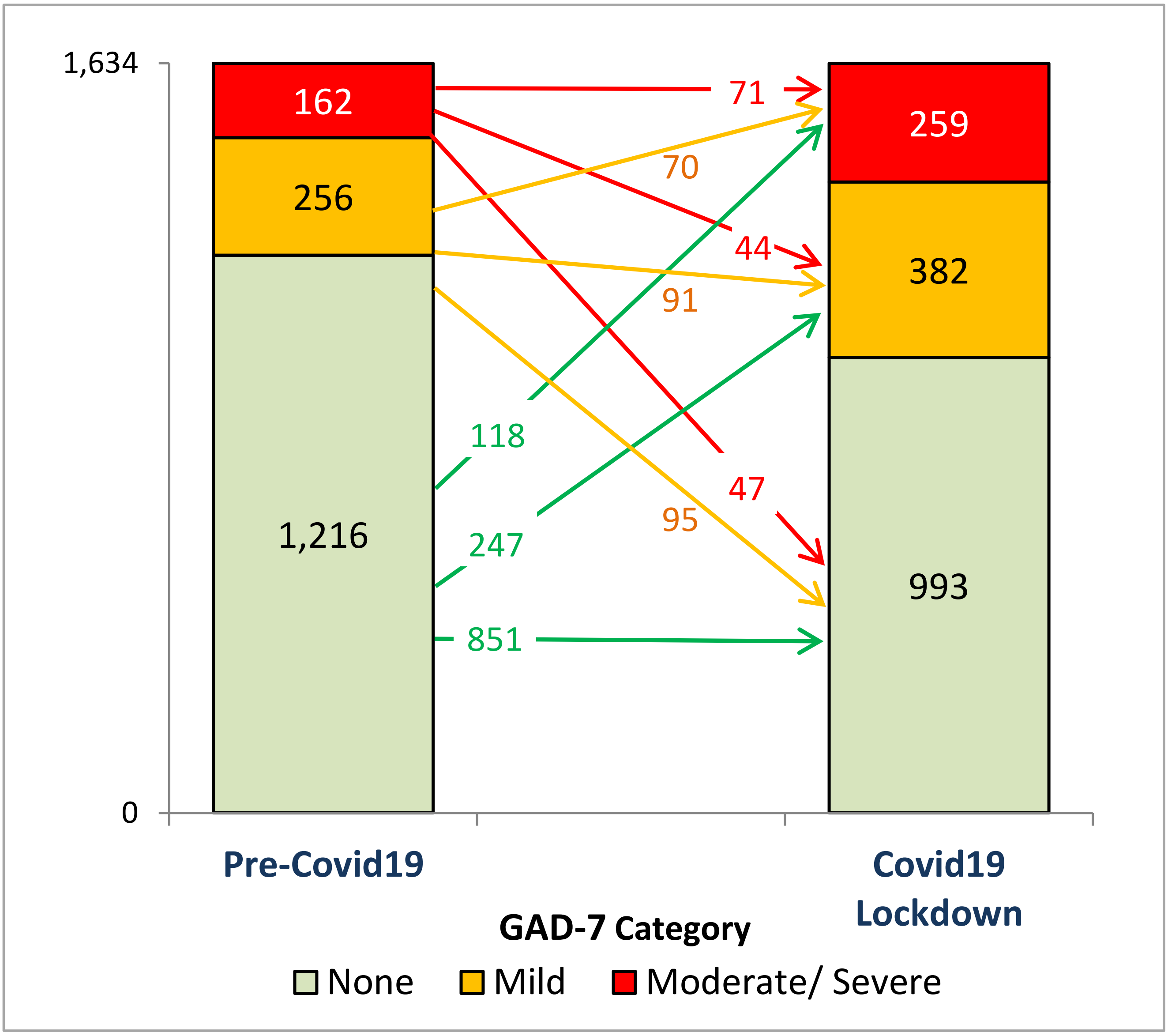
1,634
0
162
256
1,216
259
382
993
71
70
44
91
118
47
95
247
851
Pre-Covid19
Covid19 Lockdown
GAD-7 Category
None
Mild
Moderate/ Severe

Supplementary Table 1: The sample characteristics of those invited to complete the Covid-19 survey by survey completion status.

	Returned Survey (n=2144)			
**Age**	Complete survey (n=1860)	Incomplete / not linked (n=284)	Not returned survey (n=4722)	Total eligible (n=6866)
Under 30 yrs.	224 (12%)	4 (2%)	751 (16%)	963 (14%)
30 to 34 yrs.	396 (21%)	22 (10%)	1182 (25%)	1600 (24%)
35 to 39 yrs.	516 (28%)	66 (29%)	1367 (29%)	1939 (29%)
40 to 44 yrs.	423 (23%)	73 (32%)	892 (19%)	1395 (21%)
45 yrs. plus	301 (16%)	61 (27%)	495 (11%)	887 (13%)
Missing	-	58	35	82
**Ethnicity***				
White British	613 (34%)	102 (47%)	821 (18%)	1527 (23%)
Pakistani Heritage	877 (48%)	80 (37%)	2921 (64%)	3843 (59%)
Other	320 (18%)	36 (17%)	803 (18%)	1148 (18%)
Missing	50	66	177	348
**Depression (PHQ8)**				
None	1187 (66%)	-	2594 (64%)	3774 (64%)
Mild	414 (23%)	-	945 (23%)	1345 (23%)
Moderate	135 (7%)	-	346 (8%)	475 (8%)
Moderately severe	58 (3%)	-	134 (3%)	193 (3%)
Severe	19 (1%)	-	64 (2%)	83 (1%)
Missing	47	284	639	996
**Anxiety (GAD7)**				
None	1280 (75%)	-	2720 (72%)	3991 (73%)
Mild	270 (16%)	-	646 (17%)	908 (17%)
Moderate	100 (6%)	-	224 (6%)	322 (6%)
Severe	67 (4%)	-	166 (4%)	234 (4%)
Missing	143	284	966	1,411

**Note: The ethnicity representativeness is skewed by the BiBBS participants who are ~70% South Asian and had a comparatively low response rate to this survey – see reference 15 for explanation*

For peer review only

For peer review only
